# A spectrophotometric analysis of extracted water-soluble phenolic metabolites of lichens

**DOI:** 10.1007/s00425-024-04474-3

**Published:** 2024-07-02

**Authors:** Łukasz Furmanek, Paweł Czarnota, Agata Tekiela, Ireneusz Kapusta, Mark R. D. Seaward

**Affiliations:** 1 Department of Ecology and Environmental Protection, University of Rzeszów, Zelwerowicza 4 Street, 35-601 Rzeszów, Poland; 2 Present Address: Unit for Assessment of Chemical, Pharmaceutical and Biological Documentation, Department for Assessment of Medicinal Products Documentation, The Office for Registration of Medicinal Products, Medical Devices and Biocidal Products, Al. Jerozolimskie 181C, Warsaw, Poland; 3 Department of Agroecology and Forest Utilization, University of Rzeszów, Ćwiklińskiej 1A Street, 35–601 Rzeszów, Poland; 4 Department of Food Technology and Nutrition, University of Rzeszów, Ćwiklińskiej 1 Street, 35–601 Rzeszów, Poland; 5 School of Archaeological and Forensic Sciences, University of Bradford, Bradford, BD7 1DP UK

**Keywords:** Ecological approach, Lichen phenols, Lichen substances extracted with water, ‘Light bulb’ extraction method, Phenol solubility, Tea-extraction’ method

## Abstract

**Main conclusion:**

Rainwater most probably constitutes a relatively effective solvent for lichen substances in nature which have the potential to provide for human and environmental needs in the future.

**Abstract:**

The aims were (i) to test the hypothesis on the potential solubility of lichen phenolic compounds using rainwater under conditions that partly reflect the natural environment and (ii) to propose new and effective methods for the water extraction of lichen substances. The results of spectrophotometric analyses of total phenolic metabolites in rainwater-based extracts from epigeic and epiphytic lichens, employing the Folin–Ciocalteu (F.–C.) method, are presented. The water solvent was tested at three pH levels: natural, 3, and 9. Extraction methods were undertaken from two perspectives: the partial imitation of natural environmental conditions and the potential use of extraction for economic purposes. From an ecological perspective, room-temperature water extraction (‘cold’ method) was used for 10-, 60-, and 120-min extraction periods. A variant of water extraction at analogous time intervals was an ‘insolation’ with a 100W light bulb to simulate the heat energy of the sun. For economic purposes, the water extraction method used the Soxhlet apparatus and its modified version, the ‘tea-extraction’ method (‘hot’ ones). The results showed that those extractions without an external heat source were almost ineffective, but insolation over 60- and 120-min periods proved to be more effective. Both tested ‘hot’ methods also proved to be effective, especially the ‘tea-extraction’ one. Generally, an increase in the concentration of phenolic compounds in water extracts resulted from an increasing solvent pH. The results show the probable involvement of lichen substances in biogeochemical processes in nature and their promising use for a variety of human necessities.

**Supplementary Information:**

The online version contains supplementary material available at 10.1007/s00425-024-04474-3.

## Introduction

Belonging to the Fungal Kingdom, lichenized fungi (lichens) are the result of a symbiotic interaction between a structurally external fungal component (mycobiont) and a non-dominant photosynthesizing algal and/or cyanobacterial partner. The mycobiont mostly belongs to the division Ascomycota, and much less frequently to the Basidiomycota (Lücking et al. 2017). Symbiotic interactions are most commonly established by algae from division Chlorophyta (Muggia et al. 2022) as in the case of those species tested in this study, less frequently by cyanobacteria (Masumoto and Sanders 2022), and rarely by a mycobiont and two photobionts (Henskens et al. 2012; Spribille et al. 2022). However, the lichen symbiosis is considered to be an ecological complex (Hawksworth and Grube 2020) consisting of the increasingly laboratory-confirmed additional components of yeast (Spribille et al. 2016) and lichenicolous fungi (Khadhri et al. 2019), probably endolichenic fungi (Wethalawe et al. 2021 and literature cited therein), bacteria (Almendras et al. 2018), and viruses (Ponsero et al. 2021).

The unique combination of organisms from different kingdoms determines not only the unique morphology of the resulting lichen thalli, but also the specific secondary metabolism of the biosynthesized compounds, arising from the transformation of sugars by mycobionts, as well as those supplied by algae (polyols: ribitol, erythritol and sorbitol) and cyanobacteria (glucose) via photosynthesis (Wang et al. 2014; Furmanek et al. 2022a).

Primary metabolites are the basic substances of organisms, serving as building blocks (proteins), regulating organismal homeostasis in the form of vitamins and pigments (e.g., chlorophyll and carotenoids) and energy (sugars and fats) (Elix and Stocker-Wörgötter 2008; Furmanek et al. 2022a). Most of them can be dissolved when subjected to water (Elix and Stocker-Wörgötter 2008), especially hot water (Ranković and Kosanić 2019) or organic solvents (Boustie et al. 2011).

Similar sets of carotenoid pigments, such as α-carotene, β-carotene, or rubixanthin (Czeczuga et al. 1989; Czeczuga 1994), have been found. Their concentration in the thalli is dependent on external factors, e.g., the level of insolation (Czeczuga 1994), as is the case for a provitamin, and D2 and D3 vitamins in *Cladonia arbuscula* and *C. rangiferina* species (Wang et al. 2001). Additionally, the concentration of β-carotene may depend on the presence of endophytic bacteria (Pankratov et al. 2019). When water is used as a solvent, higher aliphatic fatty acids (Elix 2014) are of marginal importance, but a special approach is required to consider the solubility of sugars.

Polysaccharides biosynthesized in lichens cement the thallial structure (Spribille et al. 2020). Their presence may result from the successive biosynthesis of a ‘green’ or fungal component (Elix and Stocker-Wörgötter 2008), one of the components (Cordeiro et al. 2004, 2005; Ruthes et al. 2008) or the result of a symbiotic interaction between them (a cold-water-soluble isolichenan: Cordeiro et al. 2004, 2011). The polysaccharides represent a rather homogeneous set of sugar compounds but are specific to the lichen species in terms of quantity and monosaccharide composition (β-D-glucans for *Cladonia* spp.: Carbonero et al. 2001), independent of the overgrown substrate (*Ramalina* spp.: Cordeiro et al. 2003). Chemically, polysaccharides mainly belong to the α- and β-glucans and gallactomannans (Carbonero et al. 2001; Olafsdottir and Ingólfsdottir 2001; Cordeiro et al. 2005), and their solubility in the polar solvent–water is determined by the bond ratio (1→3) to (1→4)-α-D-glucan (Carbonero et al. 2001; Olafsdottir and Ingólfsdottir 2001 and literature cited therein), e.g., soluble isolichenin (isolichenan) (3:1 linkage ratio) (Cordeiro et al. 2004, 2005).

Potentially, due to their relatively better solubility in water than secondary metabolites (Furmanek et al. 2022a), the concentration of sugars together with polyols in the extract can account for up to nearly 60% of dissolved lichen substances (57%: Akbulut and Yildiz 2010; Boustie et al. 2011). As a result, it is reasonable to assume that sugars will, to some, albeit unknown extent, determine biological interactions between them and other substances (Furmanek et al. 2022a), thereby influencing effectiveness of an extract, suggested by the long-term use of lichens in folk medicine (Akbulut and Yildiz 2010). An example of this is the polysaccharide lichenin present in the tested (see below) epigeic *Cetraria islandica* or epiphytic *Evernia prunastri* species, with their effective extraction from ca. 45 to 60 °C (Honegger and Haisch 2001; Surayot et al. 2019), but it is not soluble in cold water (implied: lower temperature), unlike its isomer, isolichenin (Akbulut and Yildiz 2010).

Quantitatively, however, most of the carbohydrates available in dry lichen thalli are not readily soluble, and thus do not transfer to the water-based extract; the percentage of carbohydrates in the extract, compared with the overall concentration which might be biosynthesized in the thallus, is usually about 0.3–1.9% (Akbulut and Yildiz 2010). This seems to be reflected in the potential extraction of polyols (poly-OH sugars) from the lichens resulting from natural environmental conditions (Dudley and Lechowicz 1987). Lichenin constitutes the major part of the dissolved carbohydrates of *Cetraria islandica*, *C. nivalis,* and *Alectoria ochroleuca* (c. 17–34% of dry weight thalli) in contrast to species of the genus *Cladonia* (e.g., *C. arbuscula* and *C. rangiferina*), for which the lichenin fraction was only a trace amount in the water extract (Svihus and Holand 2000). Lichenin is deposited mostly in the outer parts of *Cetraria islandica* thalli (in the extracellular matrix of the peripheral cortex and in a thick outer wall layer of medullary hyphae); this may be important for its successful extraction and is probably more easily extracted carbohydrate from this species than from *Evernia prunastri* (Honegger and Haisch 2001). Extraction of polysaccharides in hot water may be especially effective (cf. methodological variants: Ullah et al. 2019).

The ecological role of biosynthesized sugars in lichens has not yet been recognized, although it seems that they might provide a carbon source for enzymatically decomposing organisms, apart from the biochemical and physiological functions as it has been recognized in plants based on the non-structural carbohydrates (Tomasella et al. 2019; Signori-Müller et al. 2021).

The possible presence in the extract of cyclic heptapeptides (i.e., microcystins) with hepatotoxic properties, biosynthesized by the cyanobacterium component of a *Nostoc* sp. (Oksanen et al. 2004), which, however, plays no role in the context of the lichen sets tested herewith, should be taken into consideration.

In the complex of water-extracted lichen substances, secondary metabolites categorized as phenolic compounds (acetone-extracted metabolites: Norouzi et al. 2020) may constitute the main part of extracts analyzed. Their biosynthesis (qualitative-quantitative) may be determined by the presence of a lichenicolous fungus in the lichen structure (e.g., *Heterocephalacria bachmannii*; Khadhri et al. 2019) or indirectly through changes in the concentration of carbohydrates (as a carbon source) biosynthesized by algae for the synthesis of polyketides (secondary metabolites of the acetate–polymalonate pathway) by the mycobiont (e.g., in *Cladonia rangiferina*; Elshobary et al. 2016).

To date, about 1000 secondary metabolites of lichens have been identified (Elix 2014; Furmanek et al. 2019, 2022a). Phenolic compounds are characterized by the presence of an aromatic ring with at least one –OH hydroxyl group attached to the ring, and, if at least two of these groups are present, they are called polyphenols (Belščak-Cvitanović et al. 2018). These polar hydroxyl groups contribute to the higher level of solubility of these compounds in water (Rundel 1978), in addition to the use of an organic solvent (Jin et al. 2013; acetone: Aoussar et al. 2018), increased temperature (usnic acid: Jin et al. 2013), and at least a pH close to alkaline (Honegger 2009). In the same light, subjecting them to a particular organic solvent, solvent pH (atranorin: Vos et al. 2018), temperature (usnic acid: Antropova et al. 2021), exposure to light (usnic acid: Begora and Fahselt 2001) and the duration of the extraction process (usnic acid: Antropova et al. 2021) may cause their structure to break down, so this becomes an important part of the extraction. A higher level of structure stability (at least for atranorin) may result from using of whole lichen thallus in the extraction process (Vos et al. 2018). The stability of the chemical structure of lichen phenolic compounds extracted with water awaits experimental testing.

The total content of phenolic compounds in lichens can be ca. 11–14% of the dry weight of the thalli (Latkowska et al. 2019), and the concentration of secondary metabolites depends, among others, on the thallus size (*Lobaria pulmonaria*: Asplund and Gauslaa 2007) and can reach even 30% (Huneck 1973). In the secondary metabolism of lichens, about 45 classes of secondary compounds (Elix 2014), and phenolic ones are biosynthesized mainly in the acetate–polymalonate pathway (Prokop’ev et al. 2018)—these include the predominant depsides, depsidones or dibenzofurans/usnic acid derivatives. Also of importance are anthraquinones or higher aliphatic fatty acids synthesized by the same route, the latter being biosynthesized in the tricarboxylic acid pathway (Elix 2014; Goga et al. 2018; Furmanek et al. 2019, 2022b, c).

With regards to the extraction of phenolic compounds—the mevalonic and the shikimic acid pathways appear to be of less importance. The former mainly includes branched terpenes, steroids and carotenoids, while the latter is dominated by fulvic acid derivatives (Elix 2014; Goga et al. 2018; Furmanek et al. 2019, 2022b, c). It is interesting to note that axenic cultures of the fungal component and its lichenized one may differ in the biosynthesis of metabolites (cf. Molina et al. 2003).

Generally, lichen secondary compounds are considered to be barely soluble in water (usnic acid: Popovici et al. 2022), but this should take into consideration in the nature of the data analyzed (cf. Kumar et al. 2014). Most of them are non-toxic (exceptions: vulpinic and secalonic acid derivatives: Elix and Stocker-Wörgötter 2008), and their individual toxicity is relative to the pH level (Gardner and Mueller 1981). It should be noted that some metabolites may show undesirable effects, such as hepatotoxicity or photosensitization (Araújo et al. 2015 and literature cited therein), which necessitate further basic research.

The ecological role of secondary compounds is often recognized as a protective function of the lichen thalli against both biotic (i.e., bacteria and fungi) (Molnár and Farkas 2010 and literature cited therein) and abiotic (sunscreen) factors (Phinney et al. 2019), due to allelochemical substances (plant-growth inhibition and stimulation effects: Pereira Peres et al. 2015; ultrastructural changes: Tigre et al. 2015) and probably relevant in optimal defense theory (Hyvärinen et al. 2000). Their concentration is dependent on environmental factors (Bjerke et al. 2005a, b; Aoussar et al. 2018; pH level and substrate of wood bark: Latkowska et al. 2019; ecotypes: Norouzi et al. 2020) and probably on the presence of lichen bacteria (Blanco et al. 2002).

Although the possibility of precipitation of lichen secondary metabolites from the thallus into the soil has been proved experimentally (Malicki 1965; Dawson et al. 1984; Garcia-Junceda and Filho 1986; Zagoskina et al. 2013), their allelopathic potential, although established (Malicki 1967, 1970), is questioned in terms of the extracted metabolite concentrations (Asplund and Wardle 2017) and their lack of antimicrobial activity in soil (Stark and Hyvärinen 2003; Stark et al. 2007). The latest hypothesis is highlighted in the light of mycelium growth dynamics in macromycetes influenced by the stimulating effect of lichen-extracted substances (Furmanek et al. 2022a).

Taking into account the above-described issues related to the low solubility of lichen substances, especially secondary compounds, the following research hypotheses were adopted for the aim of this research work: (i) rainwater can dissolve lichen substances from the thalli of various epigeic and epiphytic lichen species under favorable conditions, (ii) factors determining the solubility level of lichen substance complexes are duration of extraction time, temperature and water pH, and (iii) under natural conditions, low concentrations of lichen substances extracted from lichen thalli are expected.

The presented results contribute to the understanding of the role of the extracted substance complexes by rainfall and their contribution to the elemental (biogeochemical) cycle (see: Zavarzina et al. 2019; Furmanek et al. 2022a), as well as to revealing new application possibilities of lichen substances dissolved in a water extract.

## Materials and methods

### Lichens examined

Eighteen species were investigated, namely 10 epigeics (terricolous) (*Cetraria aculeata*, *C. islandica*, *Cladonia arbuscula*, *C. digitata*, *C. furcata*, *C. gracilis*, *C. phyllophora*, *C. rangiferina*, *C. subulata*, *C. uncialis*) and 8 epiphytes (corticolous) (*Evernia prunastri*, *Hypogymnia physodes*, *Nephromopsis chlorophylla*, *Parmelia serrana*, *Platismatia glauca*, *Pseudevernia furfuracea*, *Ramalina farinacea*, *Usnea dasopoga*). All of these were collected from the Lasy Janowskie Forest (E Poland), the Gorce National Park in the Western Carpathians (S Poland) and the Pogórze Przemyskie Foothills (SE Poland) (Table S1).

The main criterion for the selection of lichen species was their qualitative chemical profile of secondary metabolites described in the literature (Smith et al. 2009; Kosanić et al. 2013; Wirth et al. 2013; Ossowska et al. 2014; Voicu et al. 2019) (Table S1). The preliminary separation of the secondary substances extracted by acetone was performed by thin-layer chromatography (TLC) of 14 species (*Cetraria islandica*, *Cladonia arbuscula*, *C. furcata*, *C. gracilis*, *C. rangiferina*, *C. uncialis*, *E. prunastri*, *Hypogymnia physodes*, *Nephromopsis chlorophylla*, *Parmelia serrana, Platismatia glauca*, *Pseudevernia furfuracea*, *Ramalina farinacea* and *Usnea dasopoga*) in solvent system C (toluene: acetic acid, 200:30, v/v) according to the method described by Orange et al. (2001) (Fig. S1).

### Experimental procedures

In all the water and acetone extraction methods tested, weights of lichen thalli were used in an intact (unpowdered) form, being slightly crushed for practical reasons (collection, cleaning and preparation). A uniform methodology for preparing the weights for all variants of the extraction methods tested was used.

Extractions were carried out using dry and uncrushed lichen species with a weight of 1 g/100 ml of rainwater with three pH variants, i.e., natural (unmodified), acidified to pH 3 with hydrochloric acid and alkalized to pH 9 with sodium carbonate (CZDA, Chempur, Poland). Before the extraction process, the pH of rainwater was measured or modified using an Elmetron CP-401 pH-meter with a combination electrode.

The concentration of phenolic compounds extracted with water or acetone was determined spectrophotometrically (UV–Vis) using the F.–C. method (POCH, Gliwice, Poland). A UV–Vis Evolution 300 spectrophotometer (Thermo) was employed, and data interpreted using VISION software (ver. 4.20).

The experiments were conducted in two stages: (1) preliminary stage; from the extracts obtained, single biological samples were examined in three spectrophotometric replicates; (2) specific stage; for those species from which the extracts obtained in the different methodological variants reacted in the given pH environment with the F.–C. reagent. A repeated extraction procedure was carried out using the method for which the extraction (positive for total phenols in the extract) proved to be effective. At this stage, three biological samples were taken for each extract with different pH variants (three cuvettes each) and the concentration of total phenols was measured spectrophotometrically in triplicate for each cuvette. An analogous procedure for the determination of total phenols was followed for control samples of ‘clean’ rainwater at all pH levels and methodological variants of the extraction process tested.

The total phenol concentration in the extracts was expressed as gallic acid equivalent based on the calibration curve. For preparation a blank sample in a tube used 0.25 ml of rainwater (pH 3, 9, or unmodified pH) or acetone, 0.25 ml F.–C. reagent, 0.5 ml sodium carbonate (Na_2_CO_3_) and 4 ml of distilled water. The test sample, with 0.25 ml of the extract, was prepared analogously. The absorbance value of the test sample was measured at 765 nm.

The solubility process of lichen substances was determined according to two different perspectives, i.e., ecological and economic, for which different procedures were used to extract them.

### Extraction methods from an ecological point of view

For all water extracts with different pH variants, the extraction efficiency of the lichen substances was tested by two different methods.

‘Cold’ extraction was undertaken by immersing the thallus into a beaker of water at room temperature (± 21ºC) over 10 min, 60 min, and 120 min (Fig. S2). *Cetraria islandica*, *Cladonia arbuscula,* and *Hypogymnia physodes* were tested only in extraction periods of 60 and 120 min. Exceptionally, for the initial measurements (the preliminary stage) for extractions over 10-min and 60-min periods, a single spectrophotometer measurement of the phenol compounds concentration was used; for each subsequent extraction process and measurement, 3 cuvettes each measured in triplicate were performed in the analysis. In order to compare the results obtained by the ‘cold’ extraction method and serving additionally as a control test, a 10-min acetone-based extraction analogous method was carried out for 14 lichens: *Cetraria aculeata*, *C. islandica*, *Cladonia arbuscula*, *C. digitata*, *C. furcata*, *C. gracilis*, *C. rangiferina*, *C. subulata*, *C. uncialis* (epigeic species), and *Evernia prunastri*, *Hypogymnia physodes*, *Parmelia serrana*, *Platismatia glauca,* and *Pseudevernia furfuracea* (epiphytic ones).

‘Light bulb’ extraction was undertaken by heating rainwater in a beaker with a relevant lichen biomass immersed into the solvent by a 100W light bulb suspended just above the rainwater surface (Fig. S3), as proposed by Furmanek et al. (2022a). For extractions of 10- and 60-min periods, three species (*Cetraria islandica*, *Cladonia arbuscula,* and *Hypogymnia physodes*) were used, while for a 120-min period, 11 species (*Cetraria aculeata*, *C. islandica*, *Cladonia arbuscula*, *C. digitata*, *C. furcata*, *C. gracilis*, *C. phyllophora*, *C. rangiferina*, *C. subulata*, *C. uncialis,* and *Hypogymnia physodes*) were used. The temperature of rainwater in the beaker was measured once with a water thermometer just before the start of the solvent heating process (extraction) and immediately after the end of the insolation (Fig. S4). The ‘light bulb’ extraction method was conceived as a procedure to partly reflect natural environmental conditions (Furmanek et al. 2022a). Such conditions occur in areas with strong exposure to sunlight as in young *Cladonio*-*Pinetum* coniferous forests, dunes and heathlands.

### Lichen traps

**Fig. 1:**
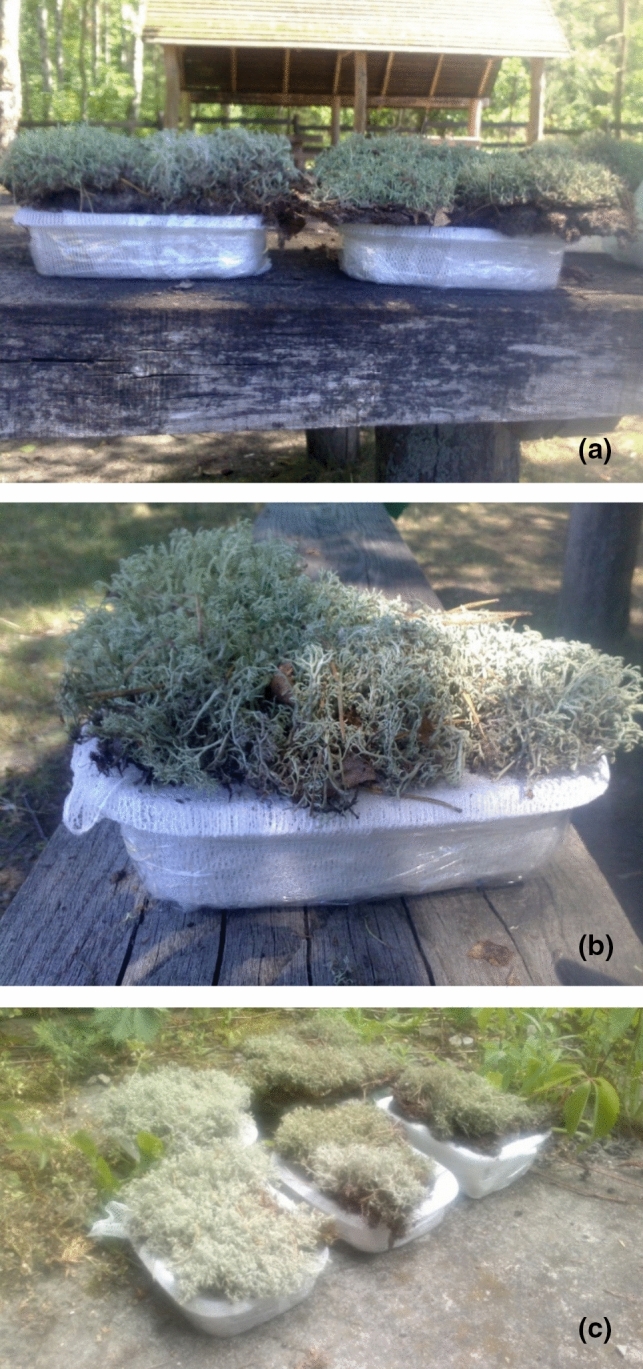
**A**–**C** ‘Lichen traps’ on a soil profile with and without various lichen species exposed to natural atmospheric conditions to obtain a water natural lichen seepage. Photos by Łukasz Furmanek

The possibility of solubility and passage of lichen substances from the various epigeic lichen species into the collected rainfall seepage under natural conditions passing through a 2–3 cm humus horizon of podzolic soil overgrown by a mat of lichens was assessed (Fig. 1A–C) for four lichen communities: (1) *Cladonia uncialis* (ca. 90% of area share) + *C. rangiferina* + *C. pyxidata* (L.) Hoffm. (the two species combined: ca. 10%), (2) *Cladonia uncialis* (ca. 90%) + *C. arbuscula* (ca. 9%) + *C. rangiferina* + *C. coniocraea* (Flörke) Spreng. + *C. pyxidata* (the three species together: ca. 1%), (3) *Cladonia uncialis* (99%) + *C. furcata* (1%), and (4) *Cetraria islandica* (100%).

These ‘lichen traps’ were prepared as follows: a layer of forest soil was dug up with a shovel together with the lichens growing on it and then placed on a ‘bandage’ which was wrapped around a plastic container and allowed rainfall to seep through the lichen and soil layer. The containers were displayed in a sunny place. As control samples, three rainfall filtrates by soil-separated thalli were used: *Cetraria islandica*, *Cladonia arbuscula,* and *C. arbuscula* + *C. gracilis*. To prevent the lichen thalli from being blown away by the wind, the container and lichens were lightly wrapped in an additional bandage (external). The filtrate was collected after each rainfall into jars to prevent evaporation.

Depending on the methodological variant and weather conditions, the collection of a minimum of 100 ml of filtrate took about one to four months. The pH of each filtrate was determined (in a measured volume of 100 ml), from which three test samples (0.25 ml of filtrate) were taken for spectrophotometric analysis to determine the concentration of phenols present. The samples were obtained after filtration as follows: (1) at room temperature, (2) heated sample in a flask over a heating coat over 30 min, and (3) heated sample with addition of 20 ml of acetone in a flask over a heating coat over 30 min.

The acetone was intended to dissolve lichen substances that had not been dissolved by rainfall but could have been potentially washed from the lichen thalli into the rainwater filtrate. Each biological sample was analyzed three times for the concentration of leached/dissolved lichen substances.

### Extraction methods from a potential economic point of view

To obtain the concentrations for economic purposes, an extraction procedure using ‘hot’ methods which included two water extraction methods: (1) the Soxhlet apparatus with a single flow of solvent through an extractor (500 ml) with lichens in a cellulose thimble for ca. 2 h (Fig. S5), and (2) a modified version of the Soxhlet apparatus—bathing rainwater-immersed thalli in the flask with a condenser therein (without the extractor and cellulose thimble parts) for an extraction period of 30 min consisting of a ± 15-min water heating period and a ± 15-min boiling period. The obtained lichen extracts were filtered independently through a gauze (‘tea-extraction’ method) (Fig. S6).

### Statistics

Statistical analysis to assess the significance of differences in the obtained total phenol compounds concentrations in the extracts was carried out to evaluate the tested extraction methods efficiency divided into two variants: (1) using pairs of the same lichen species extracted with different methods at the same rainwater pH level (unmodified, acidic, and alkaline), and (2) using rainwater pH levels (unmodified, acidic and alkaline) for the same lichen species groups investigated with a specific extraction method.

Due to the different species number extracted by the various methods, the sets compared with each other in the statistical analysis differed. The different pH values of the rainwater, ranging from 3.8 to 7.7, resulted from the use of precipitation, so that all were included in the group of rainwater with a natural pH in the statistical analyses. The acetone pH was determined as 5 (Chempur Co., Piekary Śląskie, Poland). The results which achieved zero concentration in all samples for the two tested extraction methods were not compared and therefore excluded in Tables S8–S11.

The distribution normality (for the groups being compared) was tested using the Shapiro–Wilk test for small sample sizes (*n* < 30) and the *X*^2^ and λ Kolmogorov–Smirnov tests for large ones (*n* ≥ 30). As the two sample distributions were abnormal in the tested group pairs representing different extraction methods, the non-parametric U Mann–Whitney (for a group size of *n* < 30) and the λ Kolmogorov–Smirnov tests (for a group size of *n* ≥ 30) were used to assess the statistical significance of the differences between the two compared groups. Similarly, as the samples representing the extraction effects (substance concentrations) by different methods at the three pH variants did not meet the normality requirement of the distributions, which was also checked by the tests mentioned above, the non-parametric Kruskal–Wallis test was used to analyze the significance of the differences. The significance level of α = 0.05 was adopted.

The influence of heating with a 100W light bulb for 10, 60, and 120 min on the extraction efficiency (concentration) of the substances contained in lichen thallus, was analyzed using Spearman ranks correlation, after an unsuccessful attempt to normalize the distribution of the data using rooting and logarithmic procedures. The distribution normality was checked with the Shapiro–Wilk and Kolmogorov–Smirnov tests (for small *n* < 30, and large *n* ≥ 30 samples, respectively) at the significance level of α = 0.05. The relationship was determined for *Cladonia arbuscula*, *Cetraria islandica,* and *Hypogymnia physodes* at three pH levels. All statistical analyses were performed with STATISTICA ver. 13.1. software.

## Results

### Extraction methods from an ecological viewpoint

#### ‘Cold’ extraction methods

The preliminary positive result for the presence of phenols extracted with water from lichens within 10 min using the ‘cold’ extraction method was obtained only for the mixed extract of *Cladonia arbuscula* + *C. rangiferina* + *Cetraria islandica*. The concentration was detectable in every water pH tested (Table S2). Spectrophotometric results from all other samples and the control one were negative in every rainwater pH variant. The analytical replication (for verification purposes) of the 10-min ‘cold’ extraction for the three-species mixture using natural, acidified, and alkalized rainwater did not confirm the presence of phenols. Additionally, the result for the *Cetraria islandica* extract was negative (Table S2).

The presence of phenol for most of the species tested was confirmed by the 10-min acetone extraction, performed simultaneously (Table S2). In extracts from epigeics, the phenol concentrations were relatively low, and undetectable for *Cetraria aculeata*, *C. islandica*, *Cladonia gracilis,* and *C. subulata*. The highest mean concentrations of phenols in selected epigeics were measured in the extract from *C. digitata*, which biosynthesizes thamnolic acid, and from *C. uncialis* and therein usnic acid. The results were negative for the species in which only fumarprotocetraric acid was reported, namely *Cladonia gracilis* and *C. subulata* (Smith et al. 2009), and in the case of the *C. furcata* extract, only a trace amount of this compound could be determined (Table S2).

In the extract from the mixture of *Cladonia arbuscula* + *C. rangiferina* + *Cetraria islandica*, the lichen substance concentrations were noticeably higher than in the extracts obtained from the individual lichen species. Significantly higher concentrations were found in extracts from epiphytes, especially *Hypogymnia physodes*, *Pseudevernia furfuracea,* and *Evernia prunastri*. In the first two species, at least atranorin and physodic acid may constitute the main determining substances, and for *E. prunastri* extract they were atranorin, usnic acid and evernic acid (Smith et al. 2009; acetone-TLC analysis by first author: Fig. S1).

The ‘cold’ extractions with extended time periods of up to 60 min were limited to three common European lichen species, *Cetraria islandica*, *Cladonia arbuscula,* and *Hypogymnia physodes*. Sample analyses of all extracts from *C. arbuscula* were negative. The presence of phenols was detected only in the unmodified water extract from *C. islandica* and in the alkalized one from *H. physodes*. The efficiency verification of the extraction for the latter two species showed low concentrations of phenols in only the alkalized sample obtained from *C. islandica* (Table S3).

In view of results obtained, the extraction of 120-min period was carried out only in alkaline water for several species and the thallus mixture of *Cladonia arbuscula* + *C. rangiferina* + *Cetraria islandica* showed low phenol concentrations for this three-species combination and for the extract from *Pseudevernia furfuracea* (Table S3).

#### ‘Light bulb’ extraction method

The 10-min extraction process by heating a lichen thallus immersed in rainwater with a 100W light bulb was negative for *Cetraria islandica*, *Cladonia arbuscula,* and *Hypogymnia physodes* for the three pH variants (Table S4). The mean value of rainwater temperature for all pH levels tested before insolation was 26.78 ± 1.20 °C and after heating was 40.89 ± 2.09 °C; an increment of 14.11 °C. Extending the extraction and heating time of the rainwater to 60 min increased its temperature to 49.44 ± 1.51 °C; an increment of 23.00 °C. As a result, phenols were still not detected in any of the extracts obtained from *Cladonia arbuscula*, while the result was positive for extracts from the *C. islandica* and *H. physodes*, except for *Cetraria islandica* extract at pH 9 level (Table S4).

Further extension of the experiment and heating time to 120 min did not result in a significant increase in water temperature, which reached a maximum of 51 °C (mean increment of 24.33 °C). Of the 33 tested samples obtained from the extracts from 11 lichen species (+ mixture of three lichen species), for the three pH variants, there is lack of positive results for variant at pH 3 level for extracts from 7 species (*Cetraria islandica*, *Cladonia digitata*, *C. furcata*, *C. gracilis*, *C. phyllophora*, *C. rangiferina*, *C. subulata*). Negative spectrophotometric measurements were also obtained for extracts from *Cetraria aculeata* in alkaline water and from *Cladonia uncialis* in natural and alkaline water. However, low concentrations of phenols were found in most samples, but significantly higher values when compared with terrestrial lichens were found in extracts from *Hypogymnia physodes*.

#### ‘Lichen trap’ method

No lichen compounds were detected in the rainwater seepage for all species and method combinations tested (Table S5).

### Extraction methods from a potential economic viewpoint

#### ‘Hot’ extraction: Soxhlet apparatus

A positive result from one test sample was obtained using Soxhlet apparatus for all three pH values for *Cetraria islandica*, *Cladonia digitata*, *C. furcata*, *C. gracilis*, *C. phyllophora*, a mixed extract of *Cladonia arbuscula* + *Cladonia rangiferina* + *Cetraria islandica*, *Evernia prunastri*, *Hypogymnia physodes*, *Parmelia serrana*, *Pseudevernia furfuracea,* and *Nephromopsis chlorophylla*, for natural and acidic pH extracts from *Platismatia glauca*, for acidic and alkaline pH extracts from *Usnea dasopoga* and for the acidic pH extract from *Cladonia arbuscula* (Table S6). No phenols were detected for *Cetraria aculeata*, *Cladonia rangiferina,* and *C. uncialis* extracts.

Verification based on three test samples was carried out for 12 lichen species that had positive extraction results in the pre-test extraction, and additionally for the thalli of *Cladonia subulata* (Table S6). Only samples from *C. phyllophora* immersed in natural rainwater (pH 6.9) did not show a positive result as well as samples from the extract obtained from *Platismatia glauca* in alkalized water gave negative results. The highest concentrations of phenols from a single species were found in samples from *Cladonia digitata*. Even higher ones were determined in extracts obtained from a mixture of three common species often found together in boreal habitats (*C. arbuscula*, *C. rangiferina,* and *Cetraria islandica*), despite the fact that in preliminary spectrophotometric extract analyses for single species of this composition, substances were only detected in *C. islandica*. The results for the control samples of ‘pure’ rainwater, both in the preliminary testing and in the verification phases, were negative.

The concentrations of phenols extracted from epiphytic species (*Evernia prunastri*, *Hypogymnia physodes*, *Parmelia serrana*, *Platismatia glauca*, *Pseudevernia furfuracea*, *Nephromopsis chlorophylla*) were generally significantly higher than those from terricolous species (*Cetraria islandica*, *Cladonia digitata*, *C. furcata*, *C. gracilis*, *C. phyllophora*, *C. subulata*) and from mixed three species. Overall, concentrations of phenolic compounds in the epiphytic species were more than twice as high (38.49 ± 23.40 µg ml^−1^) compared to the epigeic species (16.12 ± 14.42 µg ml^−1^) (Table S6). The highest concentration was found for extracts obtained from the cosmopolitan species *Hypogymnia physodes*. Comparing the positive results, irregularities can be observed between water pH level and the extracted phenol concentrations.

#### ‘Hot’ extraction: ‘tea-extraction’ method

Of the 17 lichen species subjected to the ‘hot’ extraction process by heating the thallus immersed in rainwater for 30 min, no phenols were found in the natural and acidic pH variants of extracts from *Cetraria aculeata*, the acidic pH extract from *Cladonia arbuscula*, and the natural pH extract from *C. uncialis* (Table S7). The control samples showed negative results.

The phenol concentrations were many times higher than those obtained by extraction using a Soxhlet apparatus and extraction by immersion of the thallus in rainwater at room temperature; an extraction over 10 min proved to be almost ineffective. Phenols were even obtained from *Cetraria aculeata*, *Cladonia rangiferina,* and *C. uncialis*. The highest concentrations of phenols in extracts from single epigeic species were found in samples from *Cetraria islandica*, and slightly lower in ones from *Cladonia digitata*. The substance concentrations from the three mixed terricolous lichens reached equally high levels. The relationships between water pH and phenol concentrations were clear, indicating an increase in the solubility of these compounds in the case of the alkalization of the rainwater-solvent.

The extraction process from epiphytic lichens was even more efficient, the highest phenol concentrations being detected in extracts from *Evernia prunastri*, *Hypogymnia physodes,* and *Pseudevernia furfuracea* (see Table S7).

### Statistical issues

There was no statistically significant difference in the efficiency of the ‘cold’ extraction between the time period variants of 10, 60, and 120 min at room temperature and between the extraction method over 60 min for the thallus immersed in water and the method with heating the solvent with a light bulb for 10 min (Table S8). Heating the water with a light bulb over 60 min resulted in significantly higher substance concentrations compared to 10- and 60-min ‘cold’ extractions and light bulb extraction over 10 min. Similarly, extending the ‘heat’ extraction time period to 120 min resulted in significantly higher substance concentrations compared to the 10-, 60-, and 120-min variants of the ‘cold’ extraction method and the light bulb method over 10 min. The light bulb extraction over 120 min proved to be less efficient than ‘hot’ extraction with the Soxhlet apparatus and the ‘tea method’. The extraction with the Soxhlet apparatus, on the other hand, was statistically more efficient compared to the method by water heating with a light bulb over 10 min and 60 min; however, compared to the variant with water heated over 120 min, no significant difference was detected. The most efficient water extraction method was the ‘tea method’, for which the level of extracted substances was significantly higher than those obtained by ‘cold’ extraction over 10 min and 60 min and compared to the Soxhlet apparatus method and light bulb for 10-, 60-, and 120-min extractions (Table 1). Higher substance concentrations from the same lichen species were only obtained in most cases for extractions with acetone (cf. Tables S2 and S8).

**Table 1:** Efficiencies of the tested extraction methods from the lichen thallus based on an analysis of the significance of differences in extracted lichen phenol concentrations for method pairs using the same species and extracts with three pH variants (unmodified, acidic, and alkalized)

Methods compared	‘Cold’ extraction 60 min (verification)	‘Cold’ extraction 120 min	‘Light bulb’ extraction 100w 10 min	‘Light bulb’ extraction 100w 60 min	‘Light bulb’ extraction 100w 120 min	‘Hot’ extraction soxhlet apparatus (verification)	‘Hot’ extraction ‘tea’ method
‘Cold’ extraction 10 min (verification)	†	‡	‡	**↑**	**↑**	**↑**	**↑**
‘Cold’ extraction 60 min (verification)		†	†	**↑**	**↑**	**↑**	**↑**
‘Cold’ extraction 120 min			‡	†	**↑**	‡	‡
‘Light bulb’ extraction 100W 10 min				↑	**↑**	↑	**↑**
‘Light bulb’ extraction 100W 60 min					†	↑	**↑**
‘Light bulb’ extraction 100W 120 min						†	**↑**
‘Hot’ extraction Soxhlet apparatus (verification)							**↑**

^†^, no statistically significant differences; ↑, statistically significant difference in favor of the indicated extraction method; ‡, methods not compared. See Table S8 for the statistical tests used

The phenol extraction performed by the ‘tea method’ was statistically significantly more effective than extraction with the Soxhlet apparatus for both epigeic and epiphytic lichens, and more effective than extraction performed over 120 min of heated water in the case of epigeic species (Table S9). The epiphytic species tested had significantly higher phenol concentrations in the extracts than the terricolous species in all comparable extraction methods (Soxhlet apparatus, ‘tea method’ and especially in acetone extraction) (Table S10). The impact of pH level upon the phenolics efficiency extraction depended on the species composition of the compared lichen groups and the extraction methods used (Table S11).

The phenol concentrations in *Cetraria islandica* and *Hypogymnia physodes* obtained from six extraction methods (Soxhlet apparatus, ‘tea method’, thallus immersion in water over 60 min and by water heating with a light bulb over 10, 60, and 120 min) showed no statistically significant differences (Table S11, group 1).

For the extracts derived from 11 lichens (*Cetraria islandica*, *Cladonia digitata*, *C. furcata*, *C. gracilis*, *C. phyllophora*, *Evernia prunastri*, *Hypogymnia physodes*, *Parmelia serrana*, *Platismatia glauca*, *Pseudevernia furfuracea* and *Nephromopsis chlorophylla*, and the mixture of *Cladonia arbuscula* + *C. rangiferina* + *Cetraria islandica*) obtained by the Soxhlet apparatus and ‘tea-extraction’ method no statistically significant differences were found between the concentrations in extracts at natural and acidic pHs and natural and alkaline pHs, but significant differences were found in concentrations between water extracts at pH 3 and pH 9 variants (Table S11, group 2).

For phenols derived from seven species (*Cetraria islandica*, *Cladonia digitata*, *C. furcata*, *C. gracilis*, *C. phyllophora*, *C. subulata,* and *Hypogymnia physodes*) and the mixture of *Cladonia arbuscula* + *C. rangiferina* + *Cetraria islandica* extracted by Soxhlet apparatus and ‘light bulb’ extraction over 120 min, statistically significant differences in concentrations occurred between extracts with natural and acidic pH variants and between acidic and alkaline ones (Table S11, group 3).

The concentrations of phenols extracted from 10 species (*Cetraria aculeata*, *C. islandica*, *Cladonia arbuscula*, *C. digitata*, *C. furcata*, *C. gracilis*, *C. phyllophora*, *C. rangiferina*, *C. uncialis,* and *Hypogymnia physodes*) and the mixture of *Cladonia arbuscula* + *C. rangiferina* + *Cetraria islandica* using the ‘tea’ method and by heating for 120 min with a light bulb showed statistically significant differences between the extracts of natural and acidic pH variants and between acidic and alkaline ones (Table S11, group 4).

A significant effect of the solvent pH on the concentration of phenols in extracts from *Cladonia arbuscula*, *Cetraria islandica* and *Hypogymnia physodes* was obtained in three extraction methods by immersing the thallus in water for 10, 60, and 120 min of heating with a light bulb; although this was demonstrated by the Kruskal–Wallis test, it was not supported by the Dunn’s post hoc test (Table S11, group 5). In cases where significant differences occurred, higher phenol concentrations were detected during extraction at a higher pH of the water.

The Spearman rank correlation used to evaluate the extraction efficiency supported by heating with a light bulb as a function of the time periods, pH variant and water temperature showed that these factors had positive but different effects, depending on the lichen species tested (Table 2). For the tested extracts from *Cladonia arbuscula*, the only significantly and strongly correlated factor was the extraction time period. For *Cetraria islandica* species, all three variables were significant, with the temperature factor having the greatest effect on the substance concentration (r_s_ = 0.73). For the epiphyte *Hypogymnia physodes,* extraction efficiency was highly significantly dependent on temperature (r_s_ = 0.82) and time period (r_s_ = 0.85).

**Table 2:** The Spearman’s rank correlation analysis (r_s_) results between the phenols contained in the water extracts from *Cladonia arbuscula*, *Cetraria islandica* and *Hypogymnia physodes* thalli by solvent heating with a light bulb over 10 min, 60 min, and 120 min, and the three factors: rainwater pH variant, extract temperature after heating and extraction time period

Factors	*Cladonia arbuscular n* = 27	*Cetraria islandica* *n* = 27	*Hypogymnia physodes* *n* = 27
Rainwater ph variant	r_s_ = 0.148013 *P* = 0.461258	r_s_ = 0.611334 *P* = 0.000705	r_s_ = 0.320461 *P* = 0.103169
Temperature	r_s_ = 0.257547 *P* = 0.194652	r_s_ = 0.734287 *P* = 0.000013	r_s_ = 0.821571 *P* = 0.000000
Extraction time period (heating)	r_s_ = 0.508710 *P* = 0.006736	r_s_ = 0.626816 *P* = 0.000468	r_s_ = 0.854423 *P* = 0.000000

The distribution normality was checked by the Shapiro–Wilk test (S–W; *P* < 0.01). The significant statistical correlation between the compared variables is shown in red. The significance level was α = 0.05 for all of the statistical tests

In all combinations tested, time strongly influenced extraction supported by heating water with a light bulb. The temperature was clearly significant for two of the three species tested. The least important factor was the solvent pH variant with a significant correlation for *Cetraria islandica* (Table 2). Based on the Spearman rank correlation test, no statistically significant effect of the extract temperature obtained after insolation on the phenols dissolved in the water was detected. However, a significant dependence of the compound concentrations on the water pH level (r_s_ ≈ 0.5) was found (Table 3).

**Table 3:** The Spearman’s rank correlation analysis (r_s_) results between the total lichen phenols obtained from the epigeic lichen species group (*Cetraria islandica*, *C. aculeata*, *Cladonia arbuscula*, *C. rangiferina*, *C. uncialis*, *C. furcata*, *C. digitata*, *C. phyllophora*, *C. gracilis*, *C. Subulata,* and a mixture of *Cladonia arbuscula* + *C. rangiferina* + *Cetraria islandica*) and from the group of all tested species combinations (epigeic species group + *Hypogymnia physodes*) and the rainwater pH variant and the extract temperature by heating with a light bulb over 120 min

Factors	Phenol concentrations obtained from all tested species combinations *n* = 108	Phenol concentrations obtained from all tested species combinations excluding *Hypogymnia physodes n* = 99
Rainwater ph variant	r_s_ = 0.492256 *P* = 0.000000	r_s_ = 0.539302 *P* = 0.000000 r_s_ = -0.119829 *P* = 0.237444
Temperature	r_s_ = -0.065746 *P* = 0.499016	

The distribution normality was checked by λ Kolmogorov–Smirnov test (K–S: *P* < 0.01). The significant correlation between the compared variables is shown in red. The significance level was α = 0.05 for the all statistical tests

## Discussion

The methods tested in the experiments for the extraction of lichen phenols can be divided into two categories: (1) possible under natural environmental conditions and (2) under laboratory conditions (including ‘hot’ extraction with the Soxhlet apparatus and the ‘tea method’).

### The issues of extraction methods from an ecological viewpoint

Amongst the extraction methods tested, both the ‘cold’ and the ‘light bulb’ methods used for shorter or longer times can be classified as those that might reflect the lichen substances extraction under natural conditions, modified by environmental influences, namely rainfall and solar insolation. Thallus immersion over 10 min in water at room temperature was practically ineffective; in a preliminary test, the presence of phenols was only detected in the extract from the mixed three terricolous species. This result indicates a slow extraction process, in a situation where the lichens are washed out by precipitation at moderate temperatures. The lack of a positive result during subsequent extraction attempts using the same method indicates that the dissolution by rainwater may depend upon other factors. Extending the water bath time to 60 min only marginally improved the results, revealing the possibility of extracting lichen compounds from *Cetraria islandica* and *Hypogymnia physodes* but not from *Cladonia arbuscula*, which was probably dependent on the solvent pH.

The ‘cold’ extraction over 120 min conducted on a larger number of species in alkalized water only confirmed that under conditions of prolonged rainfall, at higher pH values substances biosynthesized by species such as the epiphytic *Pseudevernia furfuracea* or epigeic lichens often growing in multi-species clusters, can be leached from lichen thalli. This phenomenon is confirmed by the findings of Latkowska et al. (2015) regarding compounds such as physodalic, 3-hydroxyphysodic acids or atranorin which were found in the bark of spruce trees abundantly overgrown by *Hypogymnia physodes* thalli.

Taking into account the potential environmental conditions favoring the solubility of phenols, rather small amounts of dissolved compounds originating from lichens in the soil or tree bark should be expected. However, on the basis of the analyzed data from the light bulb extraction methods, it is impossible to assess their combined allelopathic effect in ecological processes in the light of synergistic or antagonistic interactions. The results provided by the methods involving the immersion of the thallus into water only testify the possible leakage of phenols from the thallus, and indirectly their influence on ecological processes, including soil-forming ones, as suggested by experiment results of other studies (soil bacteria: Malicki 1967, 1970; general suggestion: Dawson et al. 1984)—provided favorable environmental conditions are present.

In the method by water heating with a light bulb, a temperature factor was included to simulate the heating of lichen thalli in the natural environment by means of sunlight. As shown by spectrophotometric analysis, the time period of 10 min was insufficient in terms of the solubility of phenols in *Cladonia arbuscula*, *Cetraria islandica,* and *Hypogymnia physodes*, despite increasing the water temperature by more than 14 degrees to a level of ca. 41 °C, which supports the results regarding water maceration, namely the presence of only traces of usnic acid and phenols extracted from *Usnea barbata* (positive identification: usnic, *p*-coumaric, chlorogenic and gallic acids) in the experiment by Popovici et al. (2022).

Extending the heating process for 60 min and warming the rainwater by 23 °C (from about 26 to 49 °C) resulted in the transition of phenols from thalli of *Cetraria islandica* and *Hypogymnia physodes* to the solvent in a concentration detectable by the method used, which may be due to the lichenin fraction dissolution that corresponds with the results obtained by Svihus and Holand (2000), at least for *C. islandica*. For both these species, the phenomenon of increasing phenol concentrations in the extract with increasing water pH was observed. The positive effect of prolonging the extraction time period for water heating was the reason for testing more species during 120-min insolation.

Doubling the heating time did not result in a significant increase in water temperature (+ 1.33 °C compared to 60-min insolation), and despite this, low concentrations of phenols (which may be consistent with the *Cladonia* spp. results obtained by Svihus and Holand 2000) in *C. arbuscula* extracts at all three solvent pH variants were recorded (Table S4). Above all, the extraction conditions allowed the substances included in all 12 species combinations tested to be dissolved in water. However, the solvent pH effect on phenol concentrations extracted from *C. arbuscula*, *Cetraria islandica* and *Hypogymnia physodes* during heating (for three time periods) was not statistically confirmed (see group 5, Table S11).

The additional factor of solar radiation imitation indicates that the temperature is important in the extraction of phenols from the lichen thalli. Water at ca. 50 °C has the potential to dissolve certain lichen substances, such as physodic acid and atranorin (Latkowska et al. 2015) and usnic acid (Malicki 1965) when suitable environmental conditions are maintained for about one hour. An extension of favorable conditions increases the rainwater potential as a solvent. It is likely that suitable conditions for this occur during the heavy rain that soaks into the lichen thalli and the subsequent solar insolation following the rainfall. Certain ecological niches, such as bryophyte–lichen mats in dry and fresh coniferous forests, favor water accumulation (Asplund and Wardle 2017; Klamerus-Iwan et al. 2020), which may determine the infiltration of lichen substances from the lichen thalli into the soil substrate (Malicki 1965). Extracts from lichen species that share the same chemistry as *Cladonia phyllophora*, *C. subulata,* and *C. gracilis* by the presence of fumarprotocetraric acid, showed various phenol concentrations which might be determined by external factors.

The results obtained from the water extraction of *Cladonia arbuscula*, *Cetraria islandica,* and *Hypogymnia physodes* assisted by a light bulb insolation over three time periods cannot be used directly to develop general solubility of lichen compounds patterns in heated rainwater of different pH levels in relation to the potential compound number biosynthesized in the lichen thallus (Elix 2014). However, the possibility of extracting substances from these three lichen species can presumably be compared to many species producing the same metabolites or compounds representing the same biochemical groups, with the currently proven solubility of secondary metabolites in water referring to a residual number of substances (usnic acid: Malicki 1965, 1967, 1970; other metabolites: Dawson et al. 1984). However, the detectability of these compounds may depend on their concentration in the thalli, conditioned, for example, by individual variability (Prokop'ev et al. 2018) and the influence of the substrate (Brunauer et al. 2007). More lichen species were subjected to the more efficient ‘hot’ extraction methods (with the Soxhlet apparatus and the ‘tea method’) and the method which involved heating water with a light bulb over 120 min; the solubility of phenols increased with the pH level of the rainwater-solvent.

It is possible that substances other than phenolic compounds (e.g., polysaccharides) were determined spectrophotometrically. The reason for this is the reactivity of the F.–C. reagent with other electron donor substances. The concentrations obtained cannot be interpreted as specific to phenols (Plaza et al. 2018), and their proportions relative to the other substances present in the extracts are unknown.

The potential for lichen substances leaching under natural conditions may be determined not only by duration of hydration, but also by thallus color (Geiger et al. 1995). In temperate climates, the upper surface of an epigeic lichen thallus can reach 70 °C in the summer and drop to − 20 °C in winter (Belnap and Lange 2017). According to Geiger et al. (1995), the temperature of *Cladonia furcata* thalli growing in the Black Forest in Freiburg at an altitude of 350 m reached 42 °C at 4 cm and 46 °C at 1 cm above ground level, and 66 °C for the under surface of the lichen thallus directly covering the ground. However, Kershaw (1985) reported that *C. rangiformis* warmed to 54 °C on sand heaths in Germany. The heating of *Peltigera rufescens*, growing on sandy soil, reached 60 °C, while that of *P. praetextata*, growing under favorable light conditions in a forest complex, reached c. 30 °C. The high temperature of *P. rufescens* thalli could be maintained for c. 3 h during the day, while the temperature of *P. praetextata* at ≥ 20 °C could be maintained for up to ca. 8 h per day. High temperatures can also be reached by epilithic lichens. The sun-heated, crustaceous thallus of *Circinaria calcarea* reached a temperature of 38 °C, while *Rhizocarpon superficiale*, growing on rocks at an altitude of 2500 m above sea level, reached 44 °C under full sunlight in low wind conditions.

The rainwater temperature measured in the experiments, in which lichen substances were extracted using a light bulb, reached values ranging from about 40 °C to 48 °C, depending on the heating time period. The lowest threshold of this temperature range occurred after 10 min of water heated by a light bulb. The values obtained correspond to those which are likely to occur under natural conditions.

The results obtained for the lichen substance concentrations by ‘cold’ extraction and by heating the water with a light bulb, show that the duration, temperature and pH level play a role in the extraction process. To answer the question of which of these factors is most important in this process, Spearman’s rank correlation analysis is used (Tables 2 and 3). In the case of the sparingly water-soluble lichen compounds present in *Cladonia arbuscula*, the only significant factor appeared to be the extraction time. This factor was also significantly correlated with extraction efficiency for *Cetraria islandica* and *Hypogymnia physodes*, but also significantly increased the extracted phenol concentrations in the other combinations (compare Tables S2 and S3 with Table S4).

It can be hypothesized that better water solubility of the lichen phenols diminishes the solvent pH level importance in the extraction process, as indicated by the results of the extract spectrophotometric analysis for *Hypogymnia physodes*. It can be assumed that the extraction efficiency is significantly influenced as follows: extraction time > temperature > rainwater pH level. However, due to the small amount of data obtained, it is premature to make generalizations, and presenting conclusions requires verification of the effectiveness of ‘cold’ and ‘light bulb’ extractions for a larger number of lichen species.

However, prolonging the extraction process assisted by heating the water with a light bulb over 120 min and extending the list of tested combinations to 11 species and a mixture of three epigeic species, revealed that the temperature factor had relatively little influence on the extraction result. The water pH level was statistically significant for the epigeic lichen group (7.52 ± 9.00 µg ml^−1^), and also with the inclusion of the epiphyte *Hypogymnia physodes* (9.25 ± 11.46 µg ml^−1^). This result may indicate that once the solvent temperature, and therefore the thallus, is sufficiently high, the water pH level may play an important role in their extraction. In particular, this may apply to epigeic species in which the concentrations of detectable lichen phenols are relatively low compared to epiphytes. This hypothesis needs to be verified.

Overall, the results on the solubility of lichen substances in rainwater seem to be consistent with those obtained by Hauck and Jürgens (2008) and Hauck et al. (2009a, b) on the ecological role of lichen secondary metabolites on the association of secondary compound-metal ion complexes and adaptation to changes in the pH of the external environment. The measured negligible concentrations of dissolved lichen substance complexes in acidic pH water should support the growth of most lichen species on nutrient-poor soil due to the low loss of bound metal ions in rainfall seepage.

The water solubility of phenolic compounds biosynthesized by lichens was investigated under laboratory conditions by Zagoskina et al. (2013). Mixing the thallus in distilled water over 60 min at 30 °C, these compounds were demonstrated, among others, in water extracts from *Cladonia arbuscula*, *C. gracilis*, *C. rangiferina*, *C. uncialis*, *Cetraria aculeata,* and *C. islandica*, i.e., those species in which the presence of phenols was also detected in the research described herewith, using extraction assisted by heating with a light bulb, with a Soxhlet apparatus and the ‘tea method’. According to Zagoskina et al. (2013), for these six species, water-extracted phenolic compounds accounted for 48–80% of those dissolved in 96% ethanol.

Clearly, if water was a highly efficient solvent for lichen compounds, including secondary metabolites that perform a protective role against abiotic and biotic factors, the biosynthesis of them might be unnecessary. Interestingly, in the experiments of Zagoskina et al. (2013) and Zavarzina et al. (2019) division of the tested species at the taxonomic order level in the case of Lecanorales *versus* Peltigerales showed an overall two to five times higher capacity to extract phenolic compounds in the latter, which should be partly consistent with the results of phenol compound concentrations in water extracts obtained in the case of lichens selected on an ecological basis, with, for example, lower extract concentrations in epigeics than in epiphytes.

For both quantitative and qualitative analysis of water-extracted lichen compounds, the time period of extraction is important in terms of the stability of their chemical structure; unfortunately, there are no relevant experimental data in the literature stating such a relationship. However, the stability of the atranorin structure, one of the secondary metabolites of lichens, is known to be affected by the solvent pH level; a strong acid or base added to methanol, ethanol, acetonitrile, acetone, diethyl ether and chloroform, atranorin may perform a chemical break down. The type of organic solvent used is also important for the stability of the compound (Vos et al. 2018). It is not known if the acidification and alkalization of the water affect the chemical structure stability of lichen secondary metabolites, but as Vos et al. (2018) demonstrate in the case of atranorin, a metabolite included in the complex of substances present in a lichen thallus might protect it from chemical degradation.

The temperature effect on the chemical structure stability of lichen compounds is also unknown. This is of particular importance for the interpretation of the results obtained by the ‘light bulb’, ‘tea’, and Soxhlet apparatus methods. It is not known if the temperature has any significance in nature for this phenomenon.

### ‘Lichen traps’ issues

The negative results of seepage indicate a more complicated mechanism for the solubility of lichen substances in nature (cf. Stark et al. 2007), compared to laboratory results. This process is, however, possible as demonstrated by Malicki (1965) for usnic acid, Dudley and Lechowicz (1987) for polyols, Dawson (1984) for usnic, rangiformic and psoromic acids and atranorin, as well as by Latkowska et al. (2015) in the case of several secondary metabolites derived from *Hypogymnia physodes*. It was also possible to extract atranorin and fumarprotocetraric acid from *Cladonia sprucei* with imitation rain, and to identify these compounds in soil (García-Junceda and Filho 1986).

According to Honegger (2009), lichen secondary metabolites are usually insoluble in acidic and neutral environments. The observation of the opposite phenomenon for the lichen species tested herewith indicates that the high temperature may be a factor that allows lichen substances to pass into an extract under such circumstances. The ‘light bulb’ extraction over 120 min, resulting in a temperature at 48 °C, was not very effective in acidic conditions, but under the same conditions, extraction in natural water gave positive results for 11 of the 12 species combinations tested (Table S4). Considering the possible temperature of the thallus in the natural environment and the experimental results, it can be assumed that at least some lichen substances enter the soil, but their low concentrations in lichen thalli are usually undetectable. This hypothesis is strengthened by the evidence provided by Burkin et al. (2013), Tolpysheva (2014) and Kononenko and Burkin (2015), who showed the presence of usnic acid, even in trace amounts, in all of the lichen species they tested.

### Extraction methods for potential economic issues

In the two ‘hot’ methods tested (Soxhlet apparatus and ‘tea-extraction’ method), extracts obtained from epigeic lichens were less concentrated than from epiphytic ones, which may be due to qualitative and quantitative variability in the compounds present in the thalli of different lichen species (Table S12).

The ‘tea method’ extraction process allows a ca.15-min boiling phase. In the Soxhlet apparatus, the boiled water was in the form of condensed high temperature vapor. The difference in the extraction process may be the main reason for the conditions arising in the ‘tea method’ to achieve a higher average phenol concentration in the extract for all species extracted, even taking into account the much longer extraction process by the Soxhlet apparatus (cf. phenol concentrations: Popovici et al. 2022). This corresponds with the results obtained by Antropova et al. (2021) for usnic acid solubility when the highest concentration in the water extract was achieved after boiling *Cetraria islandica* for one hour (at 100 °C). It can be assumed that the usnic acid proportion in the ‘tea extracts’ was higher when compared with the water extracts obtained from the other methods performed. However, it is interesting that the use of the Soxhlet apparatus in the water extraction for 8 h in the study by Popovici et al. (2022) did not confirm the presence of usnic acid in the total phenolic compounds from *Usnea barbata*, which could be due to the chemical structure breakdown under the influence of temperature/extraction time period and may not necessarily be a better method for extracting phenols than maceration (cf. water extracts of maceration and Soxhlet apparatus: Popovici et al. 2022, and cf. extraction methodological variants: Antropova et al. 2021 and Popovici et al. 2022); it seems to be partly consistent with the results of total phenolics concentrations presented herewith.

For both ‘hot’ extraction methods, it was most difficult to obtain substances from *Cetraria aculeata*, *Cladonia arbuscula,* and *C. uncialis*, possibly due to the low phenol concentrations in their thalli and the excessive dilution in the extract obtained. It is possible that this was to some extent influenced by the diffusion of phenols and the uneven distribution of secondary metabolites within the thalli. The solubility of lichen secondary metabolites may depend on their hydrophobic properties. Further details are planned to be discussed on this subject in a forthcoming paper.

### Other issues regarding extraction methods

The differences in the obtained concentrations in the extracts also depend on the presence of compounds with functional groups (OH, CHO, and COOH), which enhance the secondary metabolites solubility process (Iskandar and Syers 1971; Rundel 1978), conditioning a better solubility degree of metabolites with a higher molecular mass (Rundel 1978). The secondary metabolite concentration in lichen thalli also depends on the variability of meteorological conditions (temperature, solar radiation) in the different seasons, as shown for the generally higher usnic acid concentration in *Nephromopsis nivalis* in spring and summer (Bjerke et al. 2005a, b). The varying degrees of solubility for lichen secondary metabolites are also influenced by the anatomical–morphological structure of lichen thalli (Solhaug and Gauslaa 2001), which might influence the hydration time for the thallus.

The water-extracted concentrations of lichen substances indicate that the ‘tea method’ proved to be the most effective method for extracting compounds of all those species tested; among the insolation-assisted methods, that with a light bulb for 120-min extraction proved to be the most efficient. The alkaline pH of the water enhanced the extracted substance concentrations. The acetone extraction performed for the control was in most cases even more effective (cf. usnic acid concentrations: Popovici et al. 2022), but for several species of *Cetraria* and *Cladonia*, it is likely that the excessive dilution of the extracted phenols and their low concentration in the thallus, contributed to the negative results (Table S2).

In experiments using a light bulb, only its heat energy effect on the extraction efficiency was assessed, without considering the visible radiation emitted. Although a light bulb itself emits only electromagnetic radiation in the visible spectrum, solar radiation simultaneously emits spectra from other electromagnetic wavelengths. The photolability effects of UV-A and UV-B radiation on lichen secondary compounds, including those biosynthesized by *Cladonia rangiferina* and *C. uncialis* tested, are known from the experiments carried out by Begora and Fahselt (2001) which showed that usnic acid is particularly susceptible to degradation in humidified thalli. On the other hand, the synthesis of the same metabolites, such as usnic acid and atranorin, with the combined visible (white) light and UV range, can also be photo-stimulated or at least their chemical breakdown level is reduced (Begora and Fahselt 2001). In an experiment on *Cladonia cristatella*, the temperature effect rather than light appeared to be a more important factor in determining the lichen substance concentration, while the profile of the secondary metabolites was related to the age of the thalli (Culberson et al. 1983). The decomposition of extracted substances in water under the influence of both visible and different ultraviolet wavelengths remains a mystery.

In conclusion, rainwater is a solvent for lichen substances and the water extraction efficiency of these compounds depends on temperature, extraction time and rainfall pH. The higher these factor values are, the higher the lichen phenols are in the extracts, although the quality profile of phenolics was not recognized.

### Limitations and future prospects

Experiments carried out using different extraction variants with and without the use of a light bulb showed new possibilities for obtaining water extracts of lichens and their use indirectly in an ecological context (Furmanek et al. 2022a). The use of lichen thalli in an intact form was a deliberate procedure, bringing the extraction conditions closer to natural ones. The results, however, do not answer the question of whether, from the viewpoint of the rainfall effectiveness as a natural solvent of lichen substances, the heating factor by solar energy preceding rainfall is more important or the reverse. However, it can be assumed that, irrespective of the occurrence order of favorable weather factors, smaller or larger, and highly likely, trace amounts of dissolved lichen substances get from the lichen thalli into the soil or tree bark, as suggested by the negative (undetectable/diluted) results of the analysis for phenolic content of extracts from ‘lichen traps’ (Table S5).

An additional issue is the melting point depression phenomenon of individual lichen secondary metabolites. Considering the obtained water temperature in extraction using solvent heating with a light bulb and the secondary compounds melting temperature degree (Huneck and Yoshimura 1996), this is a ‘harmless’ temperature for most secondary metabolites. From this viewpoint, the analyzed dissolved and water-extracted complexes of lichen substances may, at least in part, be the result of their interaction within the thalli, enhancing their solubility process by lowering their melting point threshold, even by several tens of degrees (Allen 1942; Valderrama and Arce 2013; Nichols 2017). This phenomenon is indicated by the results obtained in the one and two hour ‘cold’ extraction periods (Table S3) and with the aid of a light bulb (Table S4).

There is a possibility of indirectly using rainwater as a solvent to impact upon the solubility of lichen substances. The reason for this seems to be the presence of different dissolved compounds contained in rainwater being absorbed from the atmosphere and their indirect determinant of substances solubility level (potentially better) than the possibility of a ‘non-ecological’ distilled water (Kristmundsdottir et al. 2005; cf. with methodology used by Stark et al. 2007). This hypothesis requires experimental verification.

The recommendation of water as a solvent for lichen substances from an ecological viewpoint is emphasized in review papers on the genera *Aspergillus* (Furmanek et al. 2022b) and *Fusarium* (Furmanek et al. 2022c), yeasts (Furmanek and Seaward 2023) and macromycetes (Furmanek et al. 2022a). The results of the present study strongly support these recommendations. Due to the relatively low concentrations of phenolic compounds in the extracts (or probably undetectable spectrophotometrically), one is unlikely to expect their dominant growth-limiting potential for fungal mycelia in nature, but rather their more frequent growth-stimulating properties. This suggestion is emphasized in respect of fungal macromycetes as intermediate organisms participating in nutrient cycling and plant succession (Furmanek et al. 2022a).

The susceptibility of fungi to the toxicity of complexes of extracted substances from lichens may be a result of their taxonomic membership in the Ascomycota and Basidiomycota divisions. Fungal species belonging to the latter have a greater capacity to enzymatically biodegrade sugar polymers (cf. *Fusarium* species—Ascomycota: Tekiela et al. 2021; Furmanek et al. 2022a), and thus are potentially more able to use them as a carbon source, as indirectly demonstrated (although methodologically different from those adopted by the authors herewith) by the results obtained by Stark and Hyvärinen (2003) and Stark et al. (2007) for *Cladonia stellaris*, an epigeic representative. When compared to the results of Stark et al. (2007), on the basis of the data obtained herewith, one would not expect a significant concentration of extracted phenolic compounds in general, and especially a higher proportion of secondary metabolites. In view of this, the negative result of soil organisms inhibition (no change in their respiration rate) is not surprising, even in the case of their successful leaching into soils; although it is evident that other factors are involved due to the presence of multi-species lichen biota found in nature, rather than a single species (cf. potential of extracts obtained from mixed lichen species: Tekiela et al. 2021; Furmanek et al. 2022a).

It is evident that in the light of the effect of lichen substances on soil organisms, it is necessary to consider their interaction as a substance complex (primary + secondary metabolites) (Furmanek et al. 2022a) and not as single compounds. Experimental water extraction methods and the extracts obtained have not yet been tested on fungi and bacteria, so that a new research phase should be initiated in the relationships of ecological processes dynamics.

Lichen extracts, especially those obtained by ‘hot’ extraction methods due to the relatively higher concentrations of phenolic compounds determined can be considered in the future as natural agents used in various industrial branches, including forestry, fungal cultivation (anti- and pro-microbial agents), food preservatives, cosmetics (such as preservatives and sunscreens), phytopharmacology (dietary supplements) and medicine (drugs and antibiotics). Numerous of these suggestions have been considered in respect of *Aspergillus*, *Fusarium*, dermatophytes (Furmanek et al. 2019; 2022b, c) and yeasts (Furmanek and Seaward 2023) and for their phytopharmaceutical value based on substances which could be obtained from *Cetraria islandica* (Antropova et al. 2021).

The natural origin of lichen substances should not burden the environment. This is crucial in an era of increasing policies to reduce artificial agents used in industry and increasing microbial resistance to currently used antibiotics and pesticides, as well as in the time of searching for new drugs. Additionally, the mycotoxins present in the lichens (Burkin and Kononenko 2014, 2015) are mostly sparingly soluble in water (Santos et al. 2022), thus reinforcing the health safety of potential water-based applications (cf. non-toxic water extracts: Kumar et al. 2014).Many attempts in the laboratory to obtain lichen water extracts against fungi or bacteria have proved unsuccessful (e.g., Sargsyan et al. 2021); although some of these provide a rationale for further research (macromycetes, genus *Aspergillus* and *Fusarium*: Furmanek et al. 2022a, b, c and literature cited therein; yeast-like fungi: Furmanek and Seaward 2023), this requires qualification. From this perspective, the results of the present study show a promising direction for further laboratory research using extracted water-soluble phenolic metabolites.

### Supplementary Information

Below is the link to the electronic supplementary material.

Supplementary file1 (DOCX 1102 KB)

Supplementary file2 (DOCX 3525 KB)

Supplementary file3 (DOCX 70 KB)

Supplementary file4 (DOCX 136 KB)

## Data Availability

Not applicable.

## Abbreviation

F.–C.: Folin–Ciocalteu method/reagent

## References

[CR1] Akbulut G, Yildiz A (2010) An overview to lichens: the nutrient composition of some species. Kafk Üniver Fen Bilim Enstit Derg 3(2):79–86

[CR2] Allen E (1942) The melting point of impure organic compounds. J Chemical Educ 19(6):278. 10.1021/ed019p278 10.1021/ed019p278

[CR3] Almendras K, García J, Carú M, Orlando J (2018) Nitrogen-fixing bacteria associated with *Peltigera* cyanolichens and *Cladonia* chlorolichens. Molecules 23:3077. 10.3390/molecules23123077 30477264 10.3390/molecules23123077 PMC6320784

[CR4] Antropova GA, Zheznyakovskaya LF, Egorova ES, Okonenko TI, Proshina LG (2021) Possibilities of using biologically active substances of Iceland Moss. IOP Conf Ser Earth Environ Sci 852:012008. 10.1088/1755-1315/852/1/012008 10.1088/1755-1315/852/1/012008

[CR5] Aoussar N, Rhallabi N, Mhand RA, Manzali R, Bouksaim M, Douira A, Mellouki F (2018) Seasonal variation of antioxidant activity and phenolic content of *Pseudevernia furfuracea*, *Evernia prunastri* and *Ramalina farinacea* from Morocco. J Saudi Soc Agric Sci 19(1):1–6. 10.1016/j.jssas.2018.03.004 10.1016/j.jssas.2018.03.004

[CR6] Araújo AAS, de Melo MGD, Rabelo TK, Nunes PS, Santos SL, Serafini MR, Santos MRV, Quintans-Júnior LJ, Gelain DP (2015) Review of the biological properties and toxicity of usnic acid. Nat Prod Res 29(23):2167–2180. 10.1080/14786419.2015.1007455 25707417 10.1080/14786419.2015.1007455

[CR7] Asplund J, Gauslaa Y (2007) Content of secondary compounds depends on thallus size in the foliose lichen *Lobaria pulmonaria*. Lichenologist 39(3):273–278. 10.1017/S0024282907006718 10.1017/S0024282907006718

[CR8] Asplund J, Wardle DA (2017) How lichens impact on terrestrial community and ecosystem properties. Biol Rev 92:1720–1738 27730713 10.1111/brv.12305

[CR9] Begora M, Fahselt D (2001) Photolability of secondary compounds in some lichen species. Symbiosis 31:3–22

[CR10] Belnap J, Lange OL (2017) Lichens and microfungi in biological soil crusts. In: Dighton J, White JF (eds) The fungal community. CRC Press, Boca Raton, Its organization and role in the ecosystem, pp 137–157

[CR11] Belščak-Cvitanović A, Durgo K, Huđek A, Bačun-Družina V, Komes D (2018) Overview of polyphenols and their properties. In: Galanakis CM (ed) Polyphenols: properties, recovery, and applications. Woodhead Publishing, Duxford, pp 3–44

[CR12] Bjerke JW, Elvebakk A, Dominguez E, Dahlback A (2005a) Seasonal trends in usnic acid concentrations of Arctic, alpine and Patagonian populations of the lichen *Flavocetraria nivalis*. Phytochemistry 66:337–344 15680990 10.1016/j.phytochem.2004.12.007

[CR13] Bjerke JW, Gwynn-Jones D, Callaghan TV (2005b) Effects of enhanced UV-B radiation in the field on the concentration of phenolics and chlorophyll fluorescence in two boreal and arctic–alpine lichens. Environ Exp Bot 53:139–149. 10.1016/j.envexpbot.2004.03.009 10.1016/j.envexpbot.2004.03.009

[CR14] Blanco Y, Blanch M, Fontaniella B, Legaz M-E et al (2002) Bioproduction of lichen phenolics by immobilized lichen cells with emphasis on the role of epiphytic bacteria. J Hattori Bot Lab 92:245–260

[CR15] Boustie J, Tomasi S, Grube M (2011) Bioactive lichens metabolites: alpine habitats as an untapped source. Phytochem Rev 10:287–307 10.1007/s11101-010-9201-1

[CR16] Brunauer G, Hager A, Grube M, Türk R, Stocker-Wörgötter E (2007) Alterations in secondary metabolism of aposymbiotically grown mycobionts of *Xanthoria elegans* and cultured resynthesis stages. Plant Physiol Biochem 45:146–151 17344057 10.1016/j.plaphy.2007.01.004

[CR17] Burkin AA, Kononenko GP (2014) Secondary fungal metabolites (mycotoxins) in lichens of different taxonomic groups. Biol Bull 41(3):216–222. 10.1134/S1062359014030030 10.1134/S1062359014030030 25731032

[CR18] Burkin AA, Kononenko GP (2015) Metabolites of toxigenic fungi in lichens of genera *Alectoria*, *Bryoria*, *Evernia*, *Pseudeverni*a, and *Usnea*. Biol Bull 42(4):296–301. 10.1134/S1062359015040032 10.1134/S1062359015040032 26415276

[CR19] Burkin AA, Kononenko GP, Tolpysheva TY (2013) Enzyme immunoassay of usnic acid in lichens. Appl Biochem Microbiol 49(3):315–321. 10.1134/S000368381303006X 10.1134/S000368381303006X 23882952

[CR20] Carbonero ER, Sassaki GL, Stuelp PM, Gorin PAJ, Woranovicz-Barreira SM, Iacomini M (2001) Comparative studies of the polysaccharides isolated from lichenized fungi of the genus *Cladonia*: significance as chemotypes. FEMS Microbiol Lett 194:65–69 11150667 10.1111/j.1574-6968.2001.tb09447.x

[CR21] Cordeiro LMC, Stocker-Wörgötter E, Gorin PAJ, Iacomini M (2003) Comparative studies of the polysaccharides from species of the genus *Ramalina*—lichenized fungi—of three distinct habitats. Phytochemistry 63:967–975. 10.1016/S0031-9422(03)00336-4 12895548 10.1016/S0031-9422(03)00336-4

[CR22] Cordeiro LMC, Stocker-Wörgötter E, Gorin PAJ, Iacomini M (2004) Elucidation of polysaccharide origin in *Ramalina peruviana* symbiosis. FEMS Microbiol Lett 238:79–84 15336406 10.1016/j.femsle.2004.07.020

[CR23] Cordeiro LMC, Carbonero ER, Sassaki GL, Reis RA, Stocker-Wörgötter E, Gorin PAJ, Iacomini M (2005) A fungus-type β-galactofuranan in the cultivated *Trebouxia* photobiont of the lichen *Ramalina gracilis*. FEMS Microbiol Lett 244:193–198 15727840 10.1016/j.femsle.2005.01.040

[CR24] Cordeiro LMC, Messias D, Sassaki GL, Gorin PAJ, Iacomini M (2011) Does aposymbiotically cultivated fungus *Ramalina* produce isolichenan? FEMS Microbiol Lett 321:50–57. 10.1111/j.1574-6968.2011.02309.x 21585515 10.1111/j.1574-6968.2011.02309.x

[CR25] Culberson CF, Culberson WL, Johnson A (1983) Genetic and environmental effects on growth and production of secondary compounds in *Cladonia cristatella*. Biochem Syst Ecol 11(2):77–84 10.1016/0305-1978(83)90003-0

[CR26] Czeczuga B (1994) Carotenoids in certain lichens of Białowieża Forest. Acta Soc Bot Pol 63(1):21–24 10.5586/asbp.1994.003

[CR27] Czeczuga B, Skirina IP, Maximov OB, Stepanenko LS (1989) Investigations on carotenoids in lichens. XIX. Carotenoids in lichens of the tundra of Kamchatka region (Far East). Phyton (austria) 29(1):7–13

[CR28] Dawson HJ, Hrutfiord BF, Ugolini FC (1984) Mobility of lichen compounds from *Cladonia mitis* in arctic soils. Soil Sci 138:40–45 10.1097/00010694-198407000-00007

[CR29] Dudley SA, Lechowicz MJ (1987) Losses of polyol through leaching in subarctic lichens. Plant Physiol 83:813–815 16665344 10.1104/pp.83.4.813 PMC1056455

[CR30] Elix JA (2014) A catalogue of standardized chromatographic data and biosynthetic relationships for lichen substances, 3rd edn. Published by the author, Canberra

[CR31] Elix JA, Stocker-Wörgötter E (2008) Biochemistry and secondary metabolites. In: Nash TH (ed) Lichen biology, 2nd edn. Cambridge University Press, Cambridge, pp 104–133

[CR32] Elshobary ME, Osman ME, Abo-Shady AM, Komatsu E et al (2016) Algal carbohydrates affect polyketide synthesis of the lichen-forming fungus *Cladonia rangiferina*. Mycologia 108(4):646–656. 10.3852/15-263 27091386 10.3852/15-263

[CR33] Furmanek Ł, Seaward MRD (2023) Anti-yeast potential of lichen-extracted substances: an analytical review. S Afr J Bot 161:720–779. 10.1016/j.sajb.2023.08.018 10.1016/j.sajb.2023.08.018

[CR34] Furmanek Ł, Czarnota P, Seaward MRD (2019) Antifungal activity of lichen compounds against dermatophytes: a review. J Appl Microbiol 127(2):308–325. 10.1111/jam.14209 30664814 10.1111/jam.14209

[CR35] Furmanek Ł, Czarnota P, Seaward MRD (2022a) Effects of lichen homogenates, mixtures of extracted substances and secondary metabolites on macromycetes: a critical review. S Afr J Bot 149:559–571. 10.1016/j.sajb.2022.06.048 10.1016/j.sajb.2022.06.048

[CR36] Furmanek Ł, Czarnota P, Seaward MRD (2022b) The effect of lichen secondary metabolites on *Aspergillus* fungi. Arch Microbiol 204:100. 10.1007/s00203-021-02649-0 10.1007/s00203-021-02649-0 PMC8716355 34964912

[CR37] Furmanek Ł, Czarnota P, Seaward MRD (2022c) A review of the potential of lichen substances as antifungal agents: the effects of extracts and lichen secondary metabolites on *Fusarium* fungi. Arch Microbiol 204:523. 10.1007/s00203-022-03104-4 35881248 10.1007/s00203-022-03104-4 PMC9325835

[CR38] Garcia-Junceda E, Filho LX (1986) Solubilization of lichen phenolics from *Cladonia sprucei* by simulated rainfall. Lichen Physiol Biochem 1:61–69

[CR39] Gardner CR, Mueller DMJ (1981) Factors affecting the toxicity of several lichen acids: effect of pH and lichen acid concentration. Am J Bot 68(1):87–95 10.1002/j.1537-2197.1981.tb06359.x

[CR40] Geiger R, Aron RH, Todhunter P (1995) The climate near the ground. Harvard University Press, Cambridge

[CR41] Goga M, Elečko J, Marcinčinová M, Ručová D, Bačkorová M, Bačkor M (2018) Lichen metabolites: an overview of some secondary metabolites and their biological potential In: Merillon JM, Ramawat K (eds) Co-evolution of secondary metabolites. Reference series in phytochemistry. Springer, Cham, pp 1–36. 10.1007/978-3-319-76887-8_57-1

[CR42] Hauck M, Jürgens SR (2008) Usnic acid controls the acidity tolerance of lichens. Environ Pollut 156(1):115–122. 10.1016/j.envpol.2007.12.033 18262699 10.1016/j.envpol.2007.12.033

[CR43] Hauck M, Jürgens SR, Willenbruch K, Huneck S, Leuschner C (2009a) Dissociation and metal-binding characteristics of yellow lichen substances suggest a relationship with site preferences of lichens. Ann Bot 103(1):13–22. 10.1093/aob/mcn202 18977765 10.1093/aob/mcn202 PMC2707280

[CR44] Hauck M, Jürgens SR, Huneck S, Leuschner C (2009b) High acidity tolerance in lichens with fumarprotocetraric, perlatolic or thamnolic acids is correlated with low pKa1 values of these lichen substances. Environ Pollut 157(10):2776–2780. 10.1016/j.envpol.2009.04.022 19464777 10.1016/j.envpol.2009.04.022

[CR45] Hawksworth D, Grube M (2020) Lichens redefined as complex ecosystems. New Phytol 227:1281–1283. 10.1111/nph.16630 32484275 10.1111/nph.16630 PMC7497170

[CR46] Henskens FL, Green TGA, Wilkins A (2012) Cyanolichens can have both cyanobacteria and green algae in a common layer as major contributors to photosynthesis. Ann Bot 110(3):555–563. 10.1093/aob/mcs108 22648879 10.1093/aob/mcs108 PMC3400443

[CR47] Honegger R, Haisch A (2001) Immunocytochemical location of the (1→3)(1→4)-β-glucan lichenin in the lichen-forming ascomycete Cetraria islandica (Icelandic moss). New Phytol 150:739–746. 10.1046/j.1469-8137.2001.00122.x 10.1046/j.1469-8137.2001.00122.x

[CR48] Honegger R (2009) Lichen-forming fungi and their photobionts In: Esser K, Deising HB (eds) The mycota. A comprehensive treatise on fungi as experimental systems for basic and applied research. Springer, Heildelberg, pp 307–333

[CR49] Huneck S (1973) Nature of lichen substances. In: Ahmadjian V, Hale ME (eds) The lichens. Academic Press, New York, London, pp 495–522

[CR50] Huneck S, Yoshimura I (1996) Identification of lichen substances. Springer-Verlag, Berlin, Heidelberg

[CR51] Hyvärinen M, Koopmann R, Hormi O, Tuomi J (2000) Phenols in reproductive and somatic structures of lichens: a case of optimal defence? Oikos 91:371–375 10.1034/j.1600-0706.2000.910217.x

[CR52] Iskandar IK, Syers JK (1971) Solubility of lichen compounds in water: pedogenetic implications. Lichenologist 5:45–50 10.1017/S0024282971000082

[CR53] Jin J-Q, Rao Y, Bian X-L, Zeng A-G (2013) Solubility of (+)-usnic acid in water, ethanol, acetone, ethyl acetate and n-hexane. J Solution Chem 42(5):1018–1027. 10.1007/s10953-013-0010-1 10.1007/s10953-013-0010-1

[CR54] Kershaw KA (1985) Physiological ecology of lichens. Cambridge University Press, Cambridge

[CR55] Khadhri A, Mendili M, Araújo MEM, Seaward MRD (2019) Comparative study of secondary metabolites and bioactive properties of the lichen *Cladonia foliacea* with and without the lichenicolous fungus *Heterocephalacria bachmannii*. Symbiosis 79:25–31. 10.1007/s13199-019-00630-6 10.1007/s13199-019-00630-6

[CR56] Klamerus-Iwan A, Kozłowski R, Przybylska J, Solarz W, Sikora W (2020) Variability of water storage capacity in three lichen species. Biologia 75:899–906 10.2478/s11756-020-00437-7

[CR57] Kononenko GP, Burkin AA (2015) Distribution of mycotoxins and usnic acid in the thalli of epigeous lichens. Biol Bull 42(3):213–219. 10.1134/S1062359015030036 10.1134/S1062359015030036 26349231

[CR58] Kosanić M, Manojlović N, Janković S, Stanojković T, Ranković B (2013) *Evernia prunastri* and *Pseudevernia furfuracea* lichens and their major metabolites as antioxidant, antimicrobial and anticancer agents. Food Chem Toxicol 53:112–118 23220145 10.1016/j.fct.2012.11.034

[CR59] Kristmundsdottir T, Jonsdottir E, Ogmundsdottir HM, Ingólfsdóttir K (2005) Solubilization of poorly soluble lichen metabolites for biological testing on cell lines. Eur J Pharm Sci 24:539–543 15784343 10.1016/j.ejps.2005.01.011

[CR60] Kumar J, Dhar P, Tayade AB, Gupta D, Chaurasia OP, Upreti DK, Arora R, Srivastava RB (2014) Antioxidant capacities, phenolic profile and cytotoxic effects of saxicolous lichens from trans-Himalayan cold desert of Ladakh. PLoS ONE. 10.1371/journal.pone.0098696 24937759 10.1371/journal.pone.0098696 PMC4061001

[CR61] Latkowska E, Bober B, Chrapusta E, Adamski M, Kaminski A, Bialczyk J (2015) Secondary metabolites of the lichens *Hypogymnia physodes* (L.) Nyl. and their presence in spruce (*Picea abies* (L.) H. Karst.) bark. Phytochemistry 118:116–123 26342621 10.1016/j.phytochem.2015.08.016

[CR62] Latkowska E, Bialczyk J, Węgrzyn M, Erychleb U (2019) Host species affects the phenolic compounds content in *Hypogymnia physodes* (L.) Nyl. thalli. Allelopathy J 47(2):221–232 10.26651/allelo.j/2019-47-2-1233

[CR63] Lücking R, Hodkinson BP, Leavitt SD (2017) The 2016 classification of lichenized fungi in the Ascomycota and Basidiomycota - approaching one thousand genera. Bryologist 119:361–416 10.1639/0007-2745-119.4.361

[CR64] Malicki J (1965) The effect of lichen acids on the soil microorganism. Part I. The washing down of the acids into the soil. Ann Univ Mariae Curie-Skłodowska Lublin-Polonia Sec C 20:239–248 (**(in Polish)**)

[CR65] Malicki J (1967) The effect of lichens acids on soil microbes. Part II. The influence of aqueous extracts from *Cladonia* species on soil bacteria. Ann Univ Mariae Curie-Skłodowska Lublin-Polonia Sec C 22(14):159–163 (**(in Polish)**)

[CR66] Malicki J (1970) The effect of lichens acids on soil microbes. Part III. The influence of the species of Cladonia genus on bacterial relation in the soil of Peucedano-Pinetum Cladonietosum association. Ann Univ Mariae Curie-Skłodowska Lublin-Polonia Sec C 25(11):76–80 (**(in Polish)**)

[CR67] Masumoto H, Sanders WB (2022) The lichen photobiont genus *Rhizonema* (cyanobacteria) exhibits diverse modes of branching, both false and true. J Phycol 58(4):612–625. 10.1111/jpy.13256 35567534 10.1111/jpy.13256

[CR68] Molina MC, Crespo A, Vicente C, Elix JA (2003) Differences in the composition of phenolics and fatty acids of cultured mycobiont and thallus of *Physconia distorta*. Plant Physiol Biochem 41:175–180 10.1016/S0981-9428(02)00017-7

[CR69] Molnár K, Farkas E (2010) Current results on biological activities of lichen secondary metabolites: a review. Z Nat Forsch 65c:157–173 10.1515/znc-2010-3-401 20469633

[CR70] Muggia L, Nelsen MP, Kirika PM, Barreno E, Beck A, Lindgren H, Lumbsch HT, Leavitt SD, Trebouxia Working Group (2022) Formally described species woefully underrepresent phylogenetic diversity in the common lichen photobiont genus *Trebouxia* (Trebouxiophyceae, Chlorophyta): an impetus for developing an integrated taxonomy. Mol Phylogenet Evol. 10.1016/j.ympev.2020.106821 10.1016/j.ympev.2020.106821 32294545

[CR71] Nichols L (2017) Organic chemistry lab techniques. LibreTexts

[CR72] Norouzi H, Azizi A, Gholami M, Sohrabi M, Boustie J (2020) Chemotype variations among lichen ecotypes of *Umbilicaria aprina* as revealed by LC-ESI-MS/MS: a survey of antioxidant phenolics. Environ Sci Poll Res 27:40296–40308. 10.1007/s11356-020-10053-2 10.1007/s11356-020-10053-2 32661964

[CR73] Oksanen I, Jokela J, Fewer DP, Wahlsten M, Rikkinen J, Sivonen K (2004) Discovery of rare and highly toxic microcystins from lichen-associated cyanobacterium *Nostoc* sp. strain IO-102-I. Appl Environ Microbiol 70:5756–5763. 10.1128/AEM.70.10.5756-5763.2004 15466511 10.1128/AEM.70.10.5756-5763.2004 PMC522101

[CR74] Olafsdottir ES, Ingólfsdottir K (2001) Polysaccharides from lichens: structural characteristics and biological activity. Planta Med 67:199–208 11345688 10.1055/s-2001-12012

[CR75] Orange A, James PW, White FJ (2001) Microchemical methods for the identification of lichens. British Lichen Society, London

[CR76] Ossowska E, Szymczyk R, Bohdan A, Kukwa M (2014) The lichen family Parmeliaceae in Poland. III. *Parmelia serrana*, new to Poland. Acta Soc Bot Pol 83(1):81–84 10.5586/asbp.2014.006

[CR77] Pankratov TA, Grouzdev DS, Patutina EO, Kolganova TV, Suzina NE, Berestovskaya JJ (2019) *Lichenibacterium ramalinae* gen. nov, sp. nov., *Lichenibacterium minor* sp. nov., the first endophytic, *beta*-carotene producing bacterial representatives from lichen thalli and the proposal of the new family Lichenibacteriaceae within the order Rhizobiales. Antonie Van Leeuwenhoek 113:477–489. 10.1007/s10482-019-01357-6 31741189 10.1007/s10482-019-01357-6

[CR78] Pereira Peres MTL, Mapeli AM, Faccenda O, Honda NK (2015) Herbicidal and plant-growth stimulating effects of phenolic compounds isolated from lichens. Orbital Electron J Chem 7(3):275–281 10.17807/orbital.v7i3.756

[CR79] Phinney NH, Gauslaa Y, Solhaug KA (2019) Why chartreuse? The pigment vulpinic acid screens blue light in the lichen *Letharia vulpina*. Planta 249:709–718. 10.1007/s00425-018-3034-3 30374913 10.1007/s00425-018-3034-3

[CR80] Plaza M, Dominguez-Rodriguez G, Castro-Puyana M, Marina ML (2018) Polyphenols analysis and related challenges. In: Galanakis CM (ed) Polyphenols: properties, recovery, and applications. Woodhead Publishing, Duxford, pp 177–232

[CR81] Ponsero AJ, Hurwitz BL, Magain N, Miadlikowska J, Lutzoni F, U’Ren JM (2021) Cyanolichen microbiome contains novel viruses that encode genes to promote microbial metabolism. ISME Commun 1:56. 10.1038/s43705-021-00060-w 37938275 10.1038/s43705-021-00060-w PMC9723557

[CR82] Popovici V, Bucur L, Gîrd CE, Popescu A, Matei E, Cozaru GC, Schröder V, Ozon EA, Fita AC, Lupuliasa D, Aschie M, Caraiane A, Botnarciuc M, Badea V (2022) Phenolic secondary metabolites and antiradical and antibacterial activities of different extracts of *Usnea barbata* (L.) Weber ex F.H. Wigg from Calimani Mountains Romania. Pharmaceuticals 15:829. 10.3390/ph15070829 35890128 10.3390/ph15070829 PMC9322614

[CR83] Prokop’ev IA, Yatsyna AP, Poryadina LN, Filippova GV, Shavarda AL (2018) Phenolic metabolites of lichens in the genus *Cladonia* growing in Belarus and Yakutia. Chem Nat Compd 54(2):362–364 10.1007/s10600-018-2347-6

[CR84] Ranković B, Kosanić M (2019) Lichens as a potential source of bioactive secondary metabolites In: Ranković B ed) Lichen secondary metabolites. Bioactive properties and pharmaceutical potential. Springer Nature, Cham, pp 1–30

[CR85] Rundel P (1978) The ecological role of secondary lichen substances. Biochem Syst Ecol 6(3):157–170 10.1016/0305-1978(78)90002-9

[CR86] Ruthes AC, Komura DL, Carbonero ER, Cordeiro LMC, Reis RA, Sassaki GL, Gorin PAJ, Iacomini M (2008) Polysaccharides present in cultivated *Teloschistes flavicans* symbiosis: comparison with those of the thallus. Plant Physiol Biochem 46:500–505. 10.1016/j.plaphy.2007.10.018 18191406 10.1016/j.plaphy.2007.10.018

[CR87] Santos AR, Carreiró F, Freitas A, Barros S, Brites C, Ramos F, Silva AS (2022) Mycotoxins contamination in rice: analytical methods, occurrence and detoxification strategies. Toxins 14:647. 10.3390/toxins14090647 36136585 10.3390/toxins14090647 PMC9504649

[CR88] Sargsyan R, Gasparyan A, Tadevosyan G, Panosyan H (2021) Antimicrobial and antioxidant potentials of non-cytotoxic extracts of corticolous lichens sampled in Armenia. AMB Express 11:110. 10.1186/s13568-021-01271-z 34324070 10.1186/s13568-021-01271-z PMC8322222

[CR89] Signori-Müller C, Oliveira RS, Barros FDV et al (2021) Non-structural carbohydrates mediate seasonal water stress across Amazon forests. Nat Commun 12:2310. 10.1038/s41467-021-22378-8 33875648 10.1038/s41467-021-22378-8 PMC8055652

[CR90] Smith CW, Aptroot A, Coppins BJ, Fletcher A, Gilbert OL, James PW, Wolseley PA, eds (2009) The lichens of Great Britain and Ireland. The British Lichen Society

[CR91] Solhaug KA, Gauslaa Y (2001) Acetone rinsing: a method for testing ecological and physiological roles of secondary compounds in living lichens. Symbiosis 30:301–315

[CR92] Spribille T, Tuovinen V, Resl P, Vanderpool D, Wolinski H, Aime MC, Schneider K, Stabentheiner E, Toome-Heller M, Thor G, Mayrhofer H, Johannesson H, McCutcheon P (2016) Basidiomycete yeasts in the cortex of ascomycete macrolichens. Science 353(6298):488–492. 10.1126/science.aaf8287 27445309 10.1126/science.aaf8287 PMC5793994

[CR93] Spribille T, Tagirdzhanova G, Goyette S, Tuovinen V, Case R, Zandberg WF (2020) 3D biofilms: in search of the polysaccharides holding together lichen symbioses. FEMS Microbiol Lett 367(5):fnaa023 32037451 10.1093/femsle/fnaa023 PMC7164778

[CR94] Spribille T, Resl P, Stanton DE, Tagirdzhanova G (2022) Evolutionary biology of lichen symbioses. New Phytol 234:1566–1582. 10.1111/nph.18048 35302240 10.1111/nph.18048

[CR95] Stark S, Hyvärinen M (2003) Are phenolics leaching from the lichen *Cladina stellaris* sources of energy rather than allelopathic agents for soil microorganisms? Soil Biol Biochem 35:1381–1385 10.1016/S0038-0717(03)00217-7

[CR96] Stark S, Kytövitta M-M, Neumann AB (2007) The phenolic compounds in *Cladonia* lichens are not antimicrobial in soils. Oecologia 152:299–306 17294219 10.1007/s00442-006-0644-4

[CR97] Surayot U, Yelithao K, Tabarsa M, Lee D-H, Palanisamy S, Prabhu NM, Lee JH, You SG (2019) Structural characterization of a polysaccharide from *Cetraria islandica* and assessment of immunostimulatory activity. Process Biochem 83:214–221. 10.1016/j.procbio.2019.05.022 10.1016/j.procbio.2019.05.022

[CR98] Svihus B, Holand Ø (2000) Lichen polysaccharides and their relation to reindeer/caribou nutrition. J Range Manag 53:642–648 10.2307/4003160

[CR99] Tekiela A, Furmanek Ł, Andrusiewicz M, Bara G, Seaward MRD, Kapusta I, Czarnota P (2021) Can lichen secondary compounds impact upon the pathogenic soil fungi *Fusarium oxysporum* and *F. avenaceum*? Folia Cryptogam Est 58:165–181 10.12697/fce.2021.58.18

[CR100] Tigre RC, Pereira EC, da Silva NH, Vicente C, Legaz ME (2015) Potential phenolic bioherbicides from *Cladonia verticillaris* produce ultrastructural changes in *Lactuca sativa* seedlings. S Afr J Bot 98:16–25. 10.1016/j.sajb.2015.02.002 10.1016/j.sajb.2015.02.002

[CR101] Tolpysheva TYu (2014) Mycotoxins and usnic acid and their distribution in lichens belonging to the genera *Cetraria*, *Flavocetraria*, and *Cladonia*. Moscow Univ Biol Sci Bull 69(3):125–129. 10.3103/S0096392514030092 10.3103/S0096392514030092

[CR102] Tomasella M, Petrussa E, Petruzzellis F, Nardini A, Casolo V (2019) The possible role of non-structural carbohydrates in the regulation of tree hydraulics. Int J Mol Sci 21(1):144. 10.3390/ijms21010144 31878253 10.3390/ijms21010144 PMC6981889

[CR103] Ullah S, Khalil AA, Shaukat F, Song Y (2019) Sources, extraction and biomedical properties of polysacharides. Foods 8:304. 10.3390/foods8080304 31374889 10.3390/foods8080304 PMC6723881

[CR104] Valderrama JO, Arce PF (2013) Melting temperature depression caused by high pressure gases. Effect of the gas on organic substances and on ionic liquids. J Supercrit Fluids 82:151–157. 10.1016/j.supflu.2013.07.007 10.1016/j.supflu.2013.07.007

[CR105] Voicu DMF, Mitoi ME, Gavriloaie C, Helepciuc FE, Toma N (2019) Chemical investigations of lichen biomass in *Usnea barbata*, *Cetraria islandica*, and *Xanthoria parietina* species. ELBA Bioflux 11(1):1–8

[CR106] Vos C, McKinney P, Pearson C, Heiny E, Gunawardena G, Holt AE (2018) The optimal extraction and stability of atranorin from lichens, in relation to solvent and pH. Lichenologist 50(4):499–512 10.1017/S0024282918000075

[CR107] Wang T, Bengtsson G, Kärnefelt I, Björn LO (2001) Provitamins and vitamins D2 and D3 in *Cladina* spp. over a latitudinal gradient: possible correlation with UV levels. J Photochem Photobiol 62(1–2):118–122. 10.1016/S1011-1344(01)00160-9 10.1016/S1011-1344(01)00160-9 11693362

[CR108] Wang Y-Y, Liu B, Zhang X-Y, Zhou Q-M, Zhang T, Li H, Yu Y-F, Zhang X-L, Hao X-Y, Wang M, Wang L, Wei J-C (2014) Genome characteristics reveal the impact of lichenization on lichen-forming fungus *Endocarpon pusillum* Hedwig (Verrucariales, Ascomycota). BMC Genom 15:34. 10.1186/1471-2164-15-34 10.1186/1471-2164-15-34 PMC3897900 24438332

[CR109] Wethalawe AN, Alwis YV, Udukala DN, Paranagama PA (2021) Antimicrobial compounds isolated from endolichenicolous fungi: a review. Molecules 26:3901. 10.3390/molecules26133901 34202392 10.3390/molecules26133901 PMC8271976

[CR110] Wirth V, Hauck M, Schultz M (2013) Die Flechten Deutschlands, Band 1–2. Ulmer Verlag, Stuttgart

[CR111] Zagoskina NV, Nikolaeva TN, Lapshin PV, Zavarzin AA, Zavarzina AG (2013) Water-soluble phenolic compounds in lichens. Microbiology 82(4):445–452 10.1134/S0026261713030132 25509379

[CR112] Zavarzina AG, Nikolaeva TN, Demin VV, Lapshin PV, Makarov MI, Zavarzin AA, Zagoskina NV (2019) Water-soluble phenolic metabolites in lichens and their potential role in soil organic matter formation at the pre-vascular stage. Eur J Soil Sci 70(4):736–750 10.1111/ejss.12822

