# Supplementary material for: A spectrophotometric analysis of extracted water-soluble phenolic metabolites of lichens
Source: Planta. 2024 Jul 2;260(2):40. doi: 10.1007/s00425-024-04474-3 (PMC11219455; doi:10.1007/s00425-024-04474-3)
Supplement: Supplementary file 1 — Supplementary file1 (DOCX 1102 KB) [file 425_2024_4474_MOESM1_ESM.docx]

Fig. S1. TLC chromatogram of the separation of acetone extracts from the lichen species thalli used in the experiment (C eluent; method according to Orange et al. 2001). The selected numbers on the starting line correspond to the following lichen species listed in Table S1: 3 – *Cladonia uncialis*, 4 – *Cladonia furcata*, 5 & 7 – *Nephromopsis chlorophylla*, 6 & 9 *– Usnea dasopoga*, 8 – *Hypogymnia physodes*, 10 – *Evernia prunastri*, 11 – *Platismatia glauca*, 13 – *Ramalina farinacea*, 14 – *Cladonia arbuscula*, 15 – *Pseudevernia furfuracea*, 16 – *Cladonia gracilis*, 17 – *Cetraria islandica*, 18 – *Cladonia rangiferina*, 20 – *Parmelia serrana*. Other numbers correspond to lichen species which was out of the scope of the article.


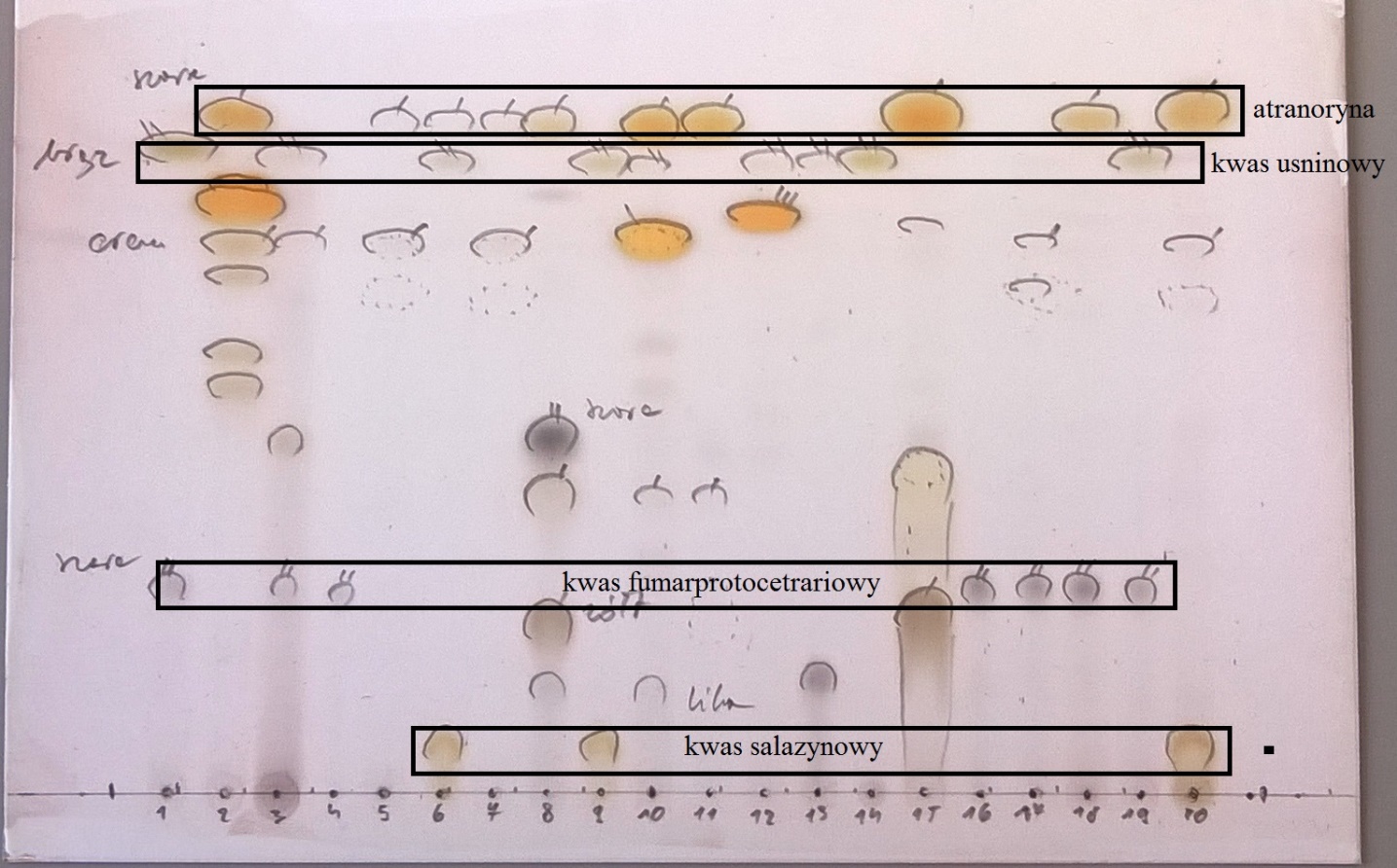


kwas ewerniowy

lichesterinic acid

lichesterinic acid

protolichesterinic acid

protolichesterinic acid

physodic acid

protocetraric acid

physodic acid

evernic acid

usnic acid

atranorin

fumarprotocetraric acid

salazanic acid
