# Supplementary material for: A spectrophotometric analysis of extracted water-soluble phenolic metabolites of lichens
Source: Planta. 2024 Jul 2;260(2):40. doi: 10.1007/s00425-024-04474-3 (PMC11219455; doi:10.1007/s00425-024-04474-3)
Supplement: Supplementary file 2 — Supplementary file2 (DOCX 3525 KB) [file 425_2024_4474_MOESM2_ESM.docx]

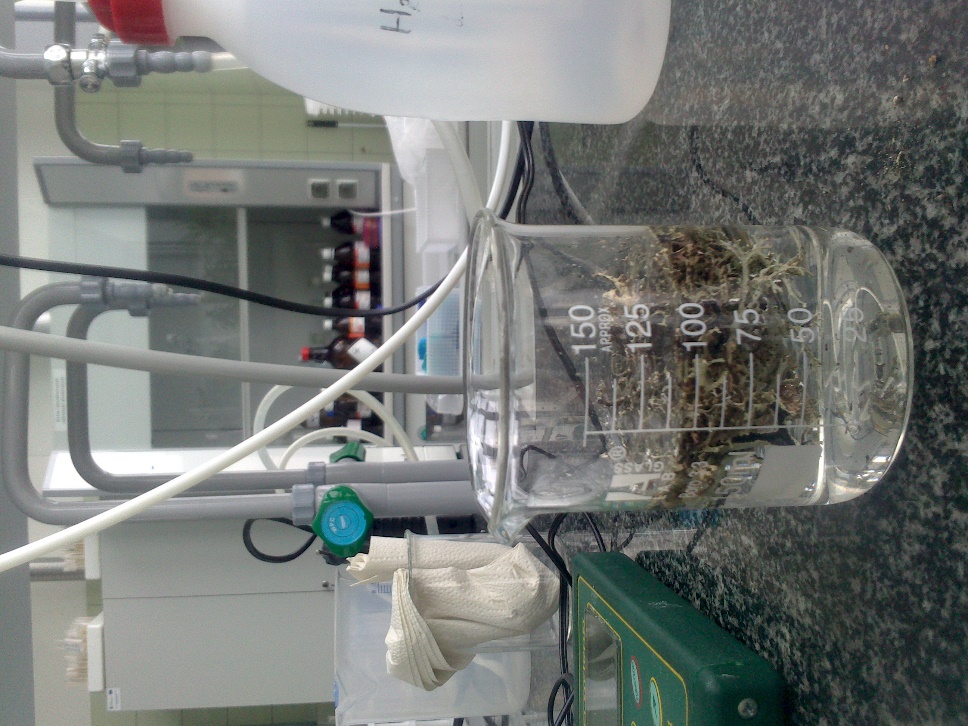


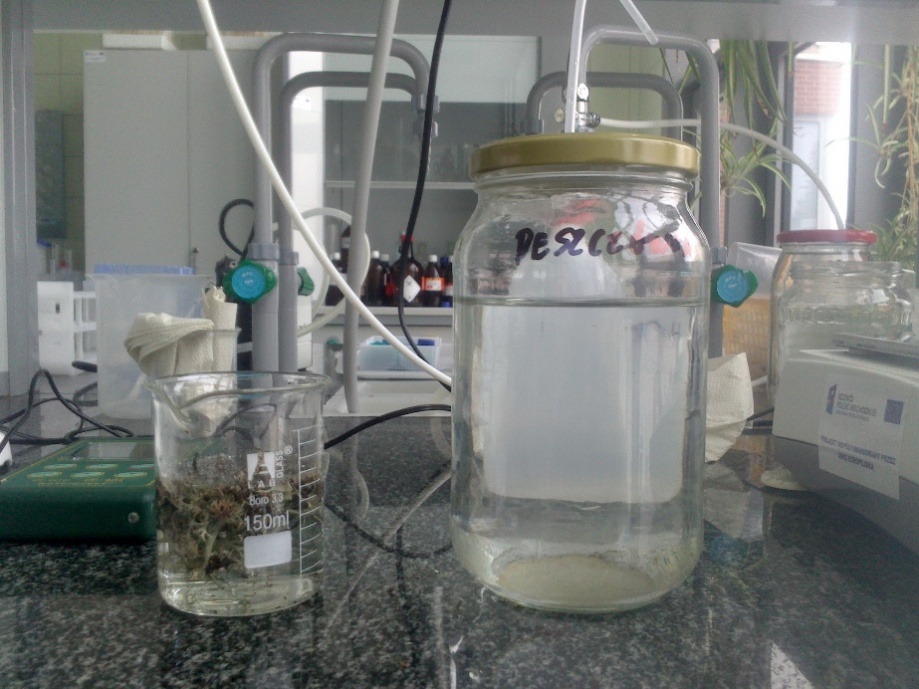


**Fig. S2.** Extraction with rainwater of the lichen substances by the ‘cold extraction’ method at room temperature ±21°C. Photos by Łukasz Furmanek


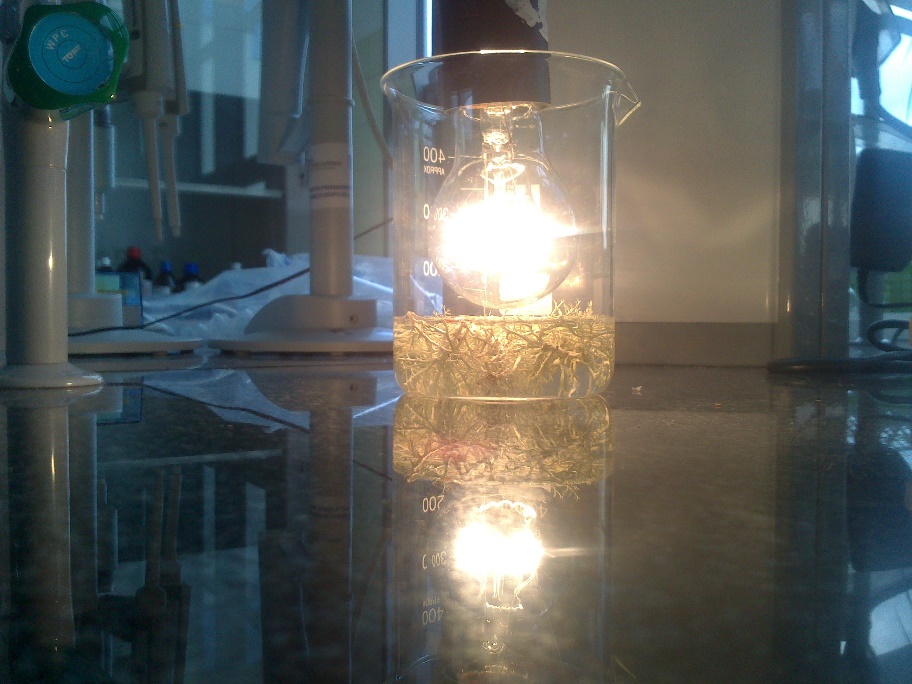


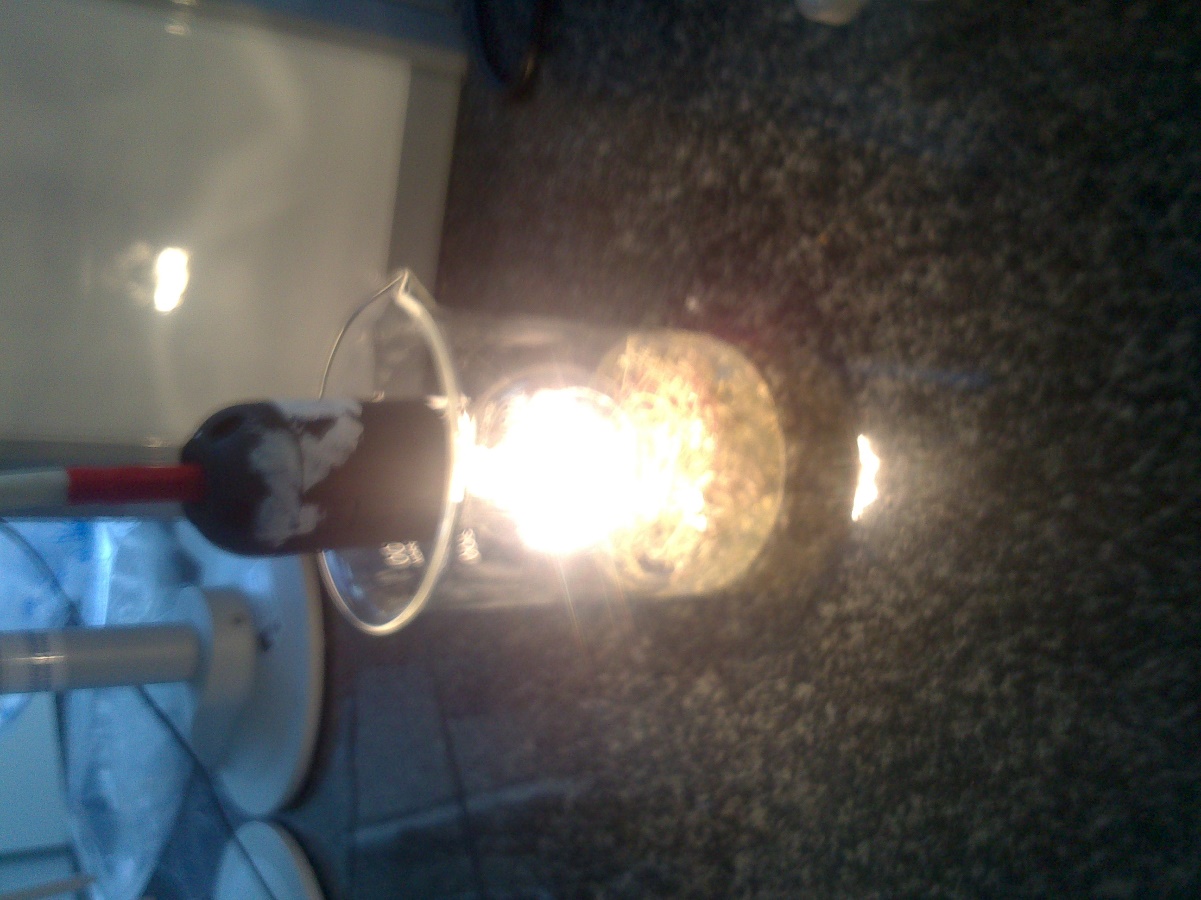


**Fig. S3.** Extraction of lichen substances with rainwater by the ‘light-bulb heating’ method. Photos by Łukasz Furmanek


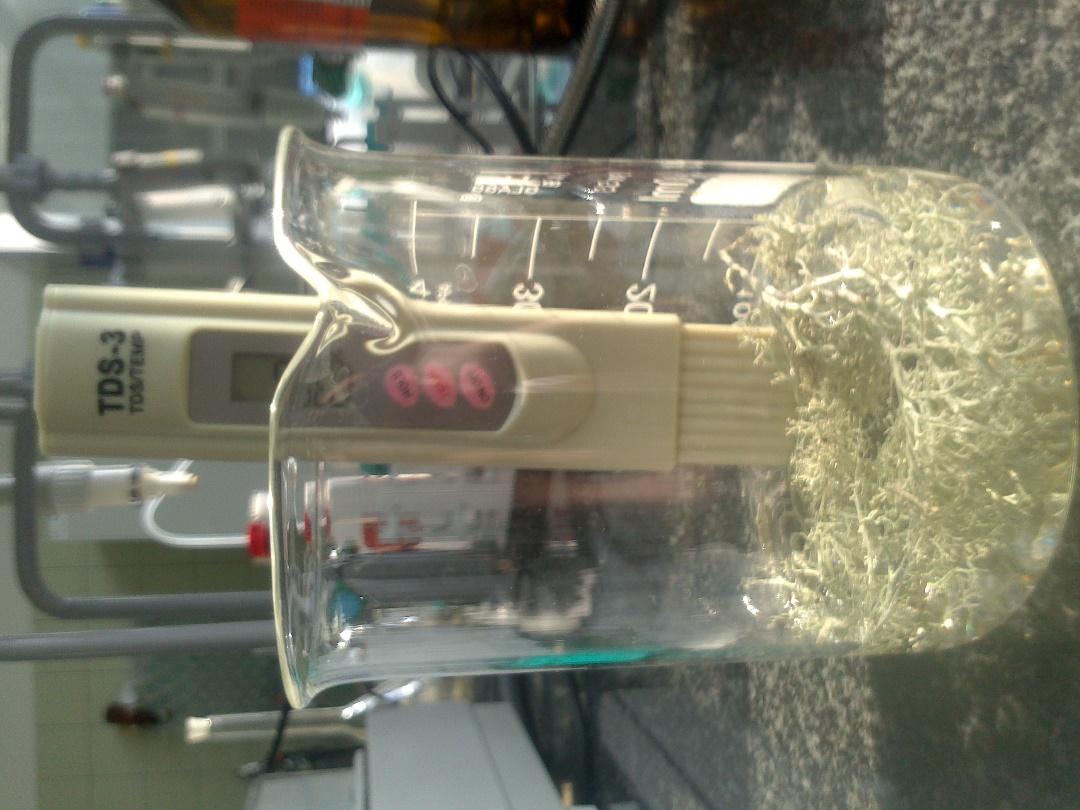

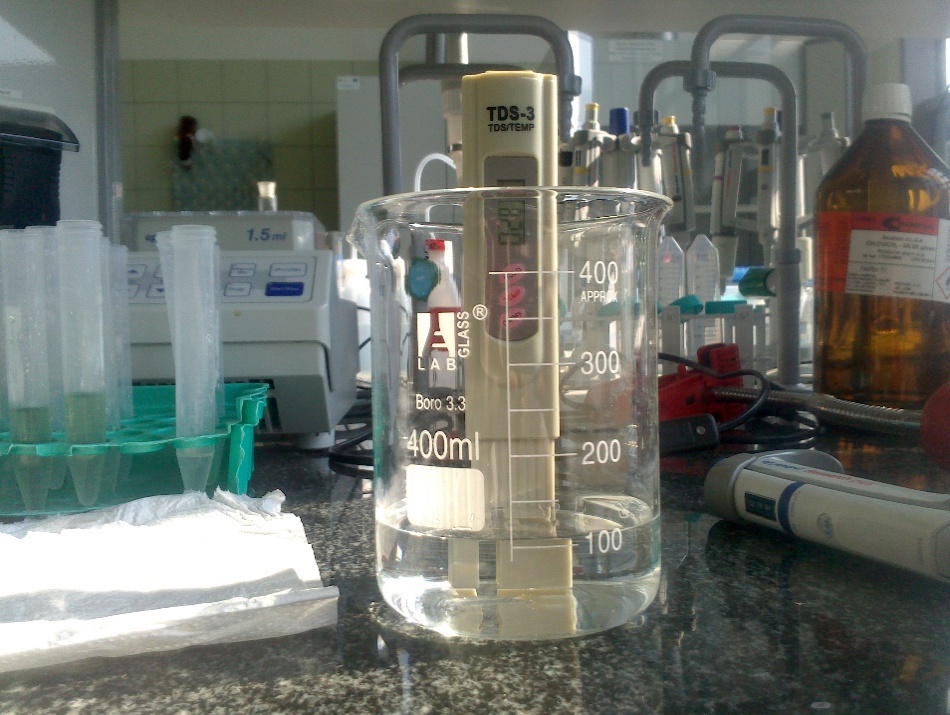


**A**

**B**

**Fig. S4.** Temperature measurement before (**A**) and immediately after (**B**) the extraction proces by using the ‘light-buld extraction’ method. Photos by Łukasz Furmanek


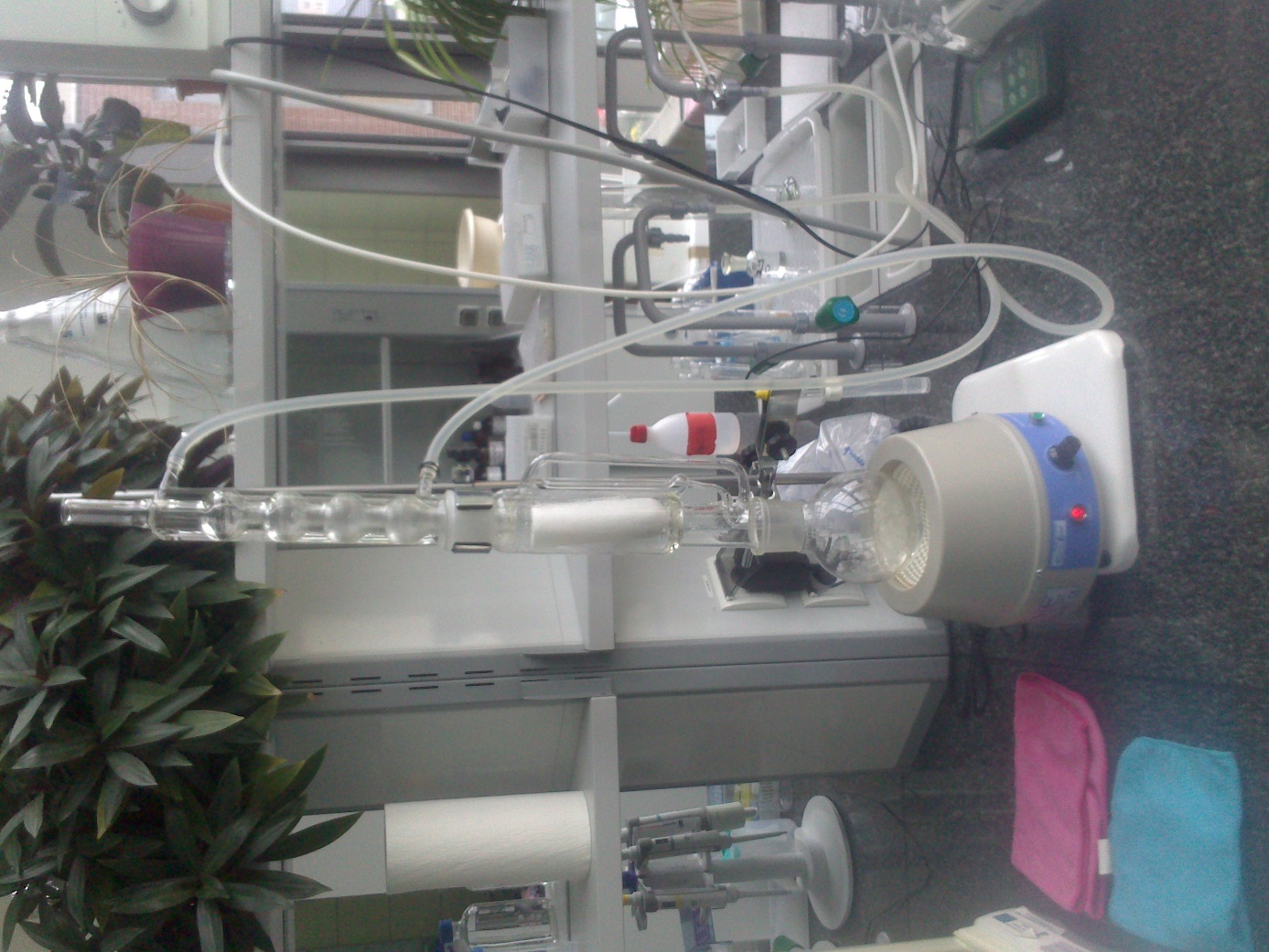


**Fig. S5.** Extraction of lichen substances with rainwater by the ‘hot extraction’ method by using a Soxhlet apparatus. Photo by Łukasz Furmanek


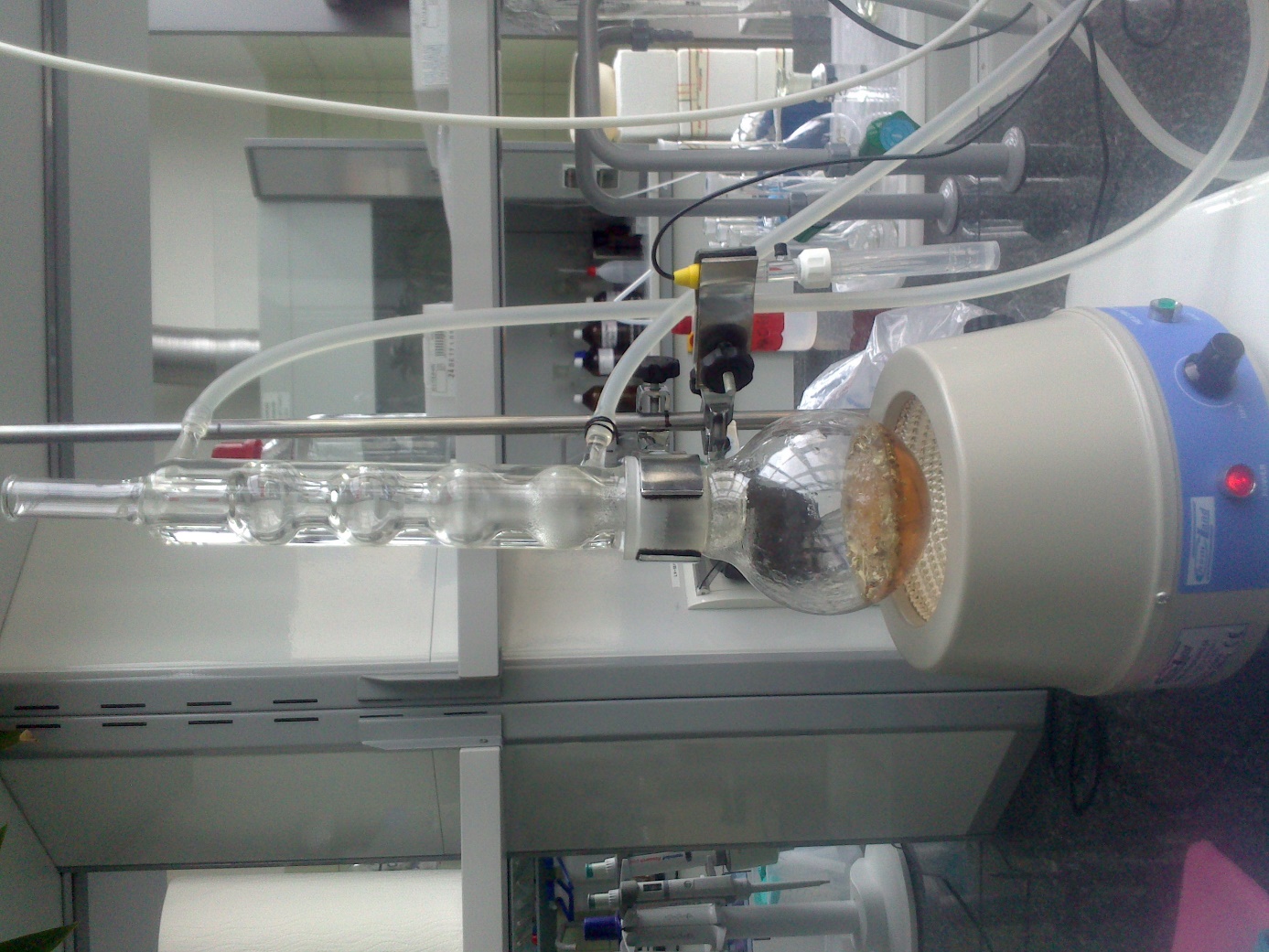


**Fig. S6.** Extraction of lichen substances with rainwater by the ‘hot extraction’ method by heating with a heating coat (‘tea method’). Photo by Łukasz Furmanek
