# Supplementary material for: A spectrophotometric analysis of extracted water-soluble phenolic metabolites of lichens
Source: Planta. 2024 Jul 2;260(2):40. doi: 10.1007/s00425-024-04474-3 (PMC11219455; doi:10.1007/s00425-024-04474-3)
Supplement: Supplementary file 3 — Supplementary file3 (DOCX 70 KB) [file 425_2024_4474_MOESM3_ESM.docx]

Table S1. Lichen species used in water and acetone extraction processes and their chemical profiles established on the basis of literature data: Smith et al. 2009; Wirth et al. 2013; Kosanić et al. 2013; Voicu et al. 2019; Ossowska et al. 2014 and own analyses using the TLC method marked with an asterisk (*).

| **Lichen species** | **Ecological group** | **Secondary metabolites** | **Harvesting place** |
| --- | --- | --- | --- |
| ***Cetraria aculeata*** (Schreber) Ach. | epigeic | lichesterinic acid  protolichesterinic acid | Janowskie Forest |
| ***Cetraria islandica*** (L.) Ach. | epigeic | fumarprotocetraric acid*  ± conprotocetraric acid  ± protocetraric acid  ± protolichesterinic acid*  ± leucotylic acid  ± 4-chlor-3-O-methylnorlicheksantone  ±cryptostictic acid  ± usnic acid | Janowskie Forest |
| ***Cladonia arbuscula*** (Wallr.) Flotow | epigeic | usnic acid*,  psoromic acid | Janowskie Forest |
| ***Cladonia digitata*** (L.) Hoffm. | epigeic  epixylic  epiphytic | thamnolic acid | Gorce Mts |
| ***Cladonia furcata*** (Huds.) Schrader | epigeic | fumarprotocetraric acid* ±atranorin | Janowskie Forest |
| ***Cladonia gracilis*** (L.) Willd. | epigeic  epixylic | fumarprotocetraric acid * | Janowskie Forest |
| ***Cladonia phyllophora*** Hoffm. | epigeic | fumarprotocetraric acid | Janowskie Forest |
| ***Cladonia rangiferina*** (L.) Nyl. | epigeic | fumarprotocetraric acid * atranorin* | Janowskie Forest |
| ***Cladonia subulata*** (L.) Weber ex F. H. Wigg. | epigeic  epixylic | fumarprotocetraric acid | Janowskie Forest |
| ***Hypogymnia physodes*** (L.) Nyl. | epiphytic  epixylic | atranorin*  chloratranorin*  physodic acid*  ± 3-hydroxyphysodic acid  ± 2'-O-metylphysodic acid  physodalic acid*  ± protocetraric acid*  ± isophysodic acid | Janowskie Forest |
| ***Nephromopsis chlorophylla*** (Willd.) Divakar, A. Crespo & Lumbsch | epiphytic,  epixylic | protolichesterinic acid * | Gorce Mts |
| ***Parmelia serrana*** A. Crespo, M. C. Molina & D. Hawksw. | epiphytic | atranorin*  salazinic acid*  consalazinic acid  lichesterinic acid  protolichesterinic acid  ± protocetraric acid | Gorce Mts |
| ***Platismatia glauca*** (L.) W.Culb. & C.Culb. | epiphytic | atranorin*  caperatic acid* | Gorce Mts |
| ***Pseudevernia furfuracea*** (L.) Zopf. | epiphytic  epixylic | atranorin*  physodic acid*  physodalic acid  hydroxyphysodic acid | Gorce Mts |
| ***Ramalina farinacea*** (L.) Ach. | epiphytic | four chemotypes:  1) usnic acid*  protocetraric acid*  2) usnic acid  salazinic acid  ± norstictic acid  3) usnic acid  hypoprotocetraric acid  4) usnic acid | Przemyskie Foothill |
| ***Usnea dasopoga*** (Ach.) Röhl. | epiphyte  epixylic | salazinic acid*  usnic acid* | Przemyskie Foothill |

Table S2. Spectrophotometric analysis of rainwater and acetone extracts from the tested lichen thalli using the Folin and Ciocalteu method for the total phenol concentration; ‘cold’ extraction over 10 min. by immersing the thallus in water at room temperature ±21°C.

Abbreviations: † – negative result; * rainwater of natural pH – no modification; ** acidic rainwater of pH 3 – modified by HCl; *** alkalised rainwater of pH 9 – modified by Na_2_CO_3_; ^‡^ arithmetic mean of three biological replicates with three biological measurements each, ±standard deviation.

| **Lichen species** | **Rainwater extraction** | | | | **Acetone extraction** |
| --- | --- | --- | --- | --- | --- |
|  | **Preliminary results** | | **Verification** | | **Phenol concentration**^‡^  **(µg ml^-1^)** |
|  | **Rainwater pH** | **Phenol concn.**  **(µg ml^-1^)** | **Rainwater pH** | **Phenol concn.**^‡^  **(µg ml^-1^)** |  |
| **epigeic species** | | | | | |
| *Cetraria aculeata* | 4.3^*^ | † |  |  |  |
|  | 3^**^ | † |  |  | † |
|  | 9^***^ | † |  |  |  |
| *Cetraria islandica* | 4.5 | † | 7.5 | † |  |
|  | 3 | † | 3 | † | † |
|  | 9 | † | 9 | † |  |
| *Cladonia arbuscula* | 4.3 | † |  |  |  |
|  | 3 | † |  |  | 6.23 ±1.85 |
|  | 9 | † |  |  |  |
| *Cladonia digitata* | 5.7 | † |  |  |  |
|  | 3 | † |  |  | 30.55 ±0.08 |
|  | 9 | † |  |  |  |
| *Cladonia furcata* | 4.9 | † |  |  |  |
|  | 3 | † |  |  | 0.35 ±0.61 |
|  | 9 | † |  |  |  |
| *Cladonia gracilis* | 5.8 | † |  |  |  |
|  | 3 | † |  |  | † |
|  | 9 | † |  |  |  |
| *Cladonia phyllophora* | 5.3 | † |  |  |  |
|  | 3 | † |  |  |  |
|  | 9 | † |  |  |  |
| *Cladonia rangiferina* | 4.9 | † |  |  |  |
|  | 3 | † |  |  | 19.46 ±6.51 |
|  | 9 | † |  |  |  |
| *Cladonia subulata* | 4.3 | † |  |  |  |
|  | 3 | † |  |  | † |
|  | 9 | † |  |  |  |
| *Cladonia uncialis* | 4.8 | † |  |  |  |
|  | 3 | † |  |  | 29.47 ±0.60 |
|  | 9 | † |  |  |  |
| *Cladonia arbuscula* + *C. rangiferina*  + *Cetraria islandica* | 5.8 | 2.46 | 7.4 | † |  |
|  | 3 | 24.37 | 3 | † | 59.44 ±4.32 |
|  | 9 | 26.09 | 9 | † |  |
| **epiphytic species** | | | | | |
| *Evernia prunastri* | 4.7 | † |  |  |  |
|  | 3 | † |  |  | 264.68 ±8.29 |
|  | 9 | † |  |  |  |
| *Hypogymnia physodes* | 4.9 | † |  |  |  |
|  | 3 | † |  |  | 440.73 ±3.41 |
|  | 9 | † |  |  |  |
| *Parmelia serrana* | 3.8 | † |  |  |  |
|  | 3 | † |  |  | 32.58 ±5.78 |
|  | 9 | † |  |  |  |
| *Platismatia glauca* | 4.2 | † |  |  |  |
|  | 3 | † |  |  | 27.86 ±1.17 |
|  | 9 | † |  |  |  |
| *Pseudevernia furfuracea* | 4.7 | † |  |  |  |
|  | 3 | † |  |  | 355.31 ±5.19 |
|  | 9 | † |  |  |  |
| *Ramalina farinacea* | 4.7 | † |  |  |  |
|  | 3 | † |  |  |  |
|  | 9 | † |  |  |  |
| *Nephromopsis chlorophylla* | 4.9 | † |  |  |  |
|  | 3 | † |  |  |  |
|  | 9 | † |  |  |  |
| *Usnea dasopoga* | 3.8 | † |  |  |  |
|  | 3 | † |  |  |  |
|  | 9 | † |  |  |  |
| Control sample  – rainwater | 5.5 | † | 5.5 | † |  |
|  | 3 | † | 3 | † |  |
|  | 9 | † | 9 | † |  |
|  |  |  |  |  |  |
| Control sample – acetone |  |  |  |  | † |
|  |  |  |  |  |  |

Table S3. Spectrophotometric analysis of rainwater extracts from the tested lichen thalli using the Folin and Ciocalteu method for the total phenol concentration; ‘cold’ extractions over 60 min and 120 min by immersing the thallus in water at room temperature ±21°C.

Abbreviations: † – negative result; * rainwater of natural pH – no modification; ** acidic rainwater of pH 3 – modified by HCl; *** alkalised rainwater of pH 9 – modified by Na_2_CO_3_; ^‡^ arithmetic mean of three biological replicates with three biological measurements each, ±standard deviation.

| **Lichen species** | **Preliminary results** | | **Verification** | |
| --- | --- | --- | --- | --- |
|  | **Rainwater pH** | **Phenol concn.**  **(µg ml^-1^)** | **Rainwater pH** | **Phenol concn.**^‡^  **(µg ml^-1^)** |
| **extraction over 60 min.** | | | | |
| *Cetraria islandica* | 5.7^*^ | 2.13 | 6.4 | † |
|  | 3^**^ | † | 3 | † |
|  | 9^***^ | † | 9 | 0.63 ±1.09 |
| *Cladonia arbuscula* | 5.3 | † |  |  |
|  | 3 | † |  |  |
|  | 9 | † |  |  |
| *Hypogymnia physodes* | 5.9 | † | 7.2 | † |
|  | 3 | † | 3 | † |
|  | 9 | 0.57 | 9 | † |
| Control sample – rainwater | 5.5 | † | 5.5 | † |
|  | 3 | † | 3 | † |
|  | 9 | † | 9 | † |
| **extraction over 120 min.** | | | | |
| *Cetraria islandica* |  |  | 9 | † |
| *Cladonia arbuscula* |  |  | 9 | † |
| *Cladonia digitata* |  |  | 9 | † |
| *Cladonia furcata* |  |  | 9 | † |
| *Cladonia rangiferina* |  |  | 9 | † |
| *Cladonia uncialis* |  |  | 9 | † |
| *Cladonia arbuscula* + *C. rangiferina* + *Cetraria islandica* | | | 9 | 7.65 ±6.73 |
| *Pseudevernia furfuracea* |  |  | 9 | 3.01 ±11.42 |
| Control sample – rainwater |  |  | 9 | † |

Table S4. Spectrophotometric analysis of rainwater extracts from the tested lichen thalli using the Folin and Ciocalteu method for the total phenol concentration; ‘light-bulb extraction’ over 10 min, 60 min and 120 min with immersing the thallus in water and water heating by a 100W bulb.

Abbreviations: † – negative result; * rainwater of natural pH – no modification; ** acidic rainwater of pH 3 – modified by HCl; *** alkalised rainwater of pH 9 – modified by Na_2_CO_3_; ^‡^ arithmetic mean of three biological replicates with three biological measurements each, ±standard deviation.

| **Lichen species** | **Rainwater pH** | **Rainwater temperature (°C) before ‘insolation’** | **Rainwater temperature (°C) after ‘insolation’** | **Phenol concn.**^‡^  **(µg ml^-1^)** | |
| --- | --- | --- | --- | --- | --- |
|  |  | **extracion over 10 min** |  |  | |
| *Cladonia arbuscula* | 6.7^*^ | 26 | 40 | † | |
|  | 3^**^ | 26 | 41 | † | |
|  | 9^***^ | 27 | 40 | † | |
| *Cetraria islandica* | 6.2 | 28 | 43 | † | |
|  | 3 | 26 | 39 | † | |
|  | 9 | 27 | 42 | † | |
| *Hypogymnia physodes* | 6.6 | 29 | 43 | † | |
|  | 3 | 25 | 37 | † | |
|  | 9 | 27 | 43 | † | |
|  | 6.6 | 22 | 22 | † | |
| Control sample – rainwater | 3 | 22 | 22 | † | |
|  | 9 | 22 | 22 | † | |
| **extraction over 60 min.** | | | | |  |
| *Cladonia arbuscula* | 5.8^*^ | 28 | 51 | † | |
|  | 3^**^ | 25 | 49 | † | |
|  | 9^***^ | 27 | 48 | † | |
| *Cetraria islandica* | 6.7 | 28 | 51 | 8.59 ±0.10 | |
|  | 3 | 25 | 48 | † | |
|  | 9 | 26 | 50 | 10.88 ±2.57 | |
| *Hypogymnia physodes* | 5.7 | 27 | 51 | 26.11 ±4.24 | |
|  | 3 | 25 | 47 | 6.82 ±1.44 | |
|  | 9 | 27 | 50 | 24.45 ±0.28 | |
| Control sample – rainwater | 6.6 | 22 | 22 | † | |
|  | 3 | 22 | 22 | † | |
|  | 9 | 22 | 22 | † | |
| **extraction over 120 min.** | | | | |  |
| *Cetraria aculeata* | 4.6^*^ | 22 | 50 | 5.81 ±1.97^‡^ | |
|  | 3^**^ | 22 | 49 | 1.65 ±0.67 | |
|  | 9^***^ | 23 | 50 | † | |
| *Cetraria islandica* | 7.0 | 23 | 49 | 19.44 ±1.90 | |
|  | 3 | 25 | 49 | † | |
|  | 9 | 25 | 51 | 17.30 ±3.51 | |
| *Cladonia arbuscula* | 6.5 | 30 | 51 | 0.79 ±1.180 | |
|  | 3 | 26 | 48 | 0.44 ±0.67 | |
|  | 9 | 23 | 49 | 2.39 ±2.45 | |
| *Cladonia digitata* | 6.1 | 26 | 49 | 21.33 ±4.64 | |
|  | 3 | 25 | 47 | † | |
|  | 9 | 26 | 49 | 30.78 ±1.52 | |
| *Cladonia furcata* | 7.1 | 25 | 48 | 11.60 ±3.41 | |
|  | 3 | 24 | 47 | † | |
|  | 9 | 23 | 49 | 9.40 ±2.22 | |
| *Cladonia gracilis* | 4.7 | 23 | 47 | 4.42 ±5.31 | |
|  | 3 | 23 | 50 | † | |
|  | 9 | 22 | 47 | 13.53 ±0.32 | |
| *Cladonia phyllophora* | 5.9 | 25 | 49 | 16.31 ±1.88 | |
|  | 3 | 25 | 51 | † | |
|  | 9 | 28 | 47 | 19.72 ±1.00 | |
| *Cladonia rangiferina* | 6.7 | 23 | 50 | 5.54 ±2.07 | |
|  | 3 | 25 | 47 | † | |
|  | 9 | 25 | 49 | 12.69 ±2.07 | |
| *Cladonia subulata* | 6.9 | 23 | 48 | 2.63 ±1.47 | |
|  | 3 | 23 | 50 | † | |
|  | 9 | 23 | 51 | 13.20 ±0.79 | |
| *Cladonia uncialis* | 7.1 | 25 | 51 | † | |
|  | 3 | 26 | 51 | 1.35 ±1.44 | |
|  | 9 | 25 | 47 | † | |
| *Cladonia arbuscula* + *C. rangiferina* + *Cetraria islandica* | 6.8 | 23 | 46 | 3.20 ±2.88 | |
|  | 3 | 23 | 49 | 6.95 ±2.40 | |
|  | 9 | 26 | 46 | 27.54 ±1.42 | |
| *Hypogymnia physodes* | 6.9 | 29 | 51 | 43.27 ±1.36 | |
|  | 3 | 28 | 50 | 17.00 ±0.72 | |
|  | 9 | 22 | 47 | 33.13 ±1.72 | |
| Control sample – rainwater | 6.6 | 23 | 22 | † | |
|  | 3 | 23 | 22 | † | |
|  | 9 | 23 | 22 | † | |

Table S5. Spectrophotometric analysis of rainwater seepage through soil covered with a lichen mat using the Folin and Ciocalteu method for the total phenol concentration; the following methodological variants were tested:
1 - seepage at room temperature, 2 - seepage heated over heating coat over 30 min., 3 - seepage heated over
heating coat over 30 min with the acetone addition; † – negative result.

| **Species combinations** | **Seepage pH** | **Extraction methodological variant** | | |
| --- | --- | --- | --- | --- |
|  |  | **1** | **2** | **3** |
| soil profile – thickness c. 2 cm  *Cladonia uncialis* (90%) *+ C. rangiferina  + C. pyxidata* (others 10%) | 5.7 | † | † | † |
| soil profile – thickness c. 2 cm  *Cladonia uncialis* (90%) *+ C. arbuscula* (9%) *+ C. rangiferina + C. coniocrea  + C. pyxidata* (others 1%) | 5.6 | † | † | † |
| soil profile – thickness c. 3 cm  *Cladonia uncialis* (99%) + *C. furcata* (1%) | 5.8 | † | † | † |
| soil profile – thickness c. 3 cm  *Cetraria islandica* (100%) | 5.2 | † | † | † |
| thallus only  *Cladonia arbuscula* (100%) | 5.9 | † | † | † |
| thallus only  *Cladonia arbuscula* (99%) + *C. gracilis* (1%) | 7.5 | † | † | † |
| thallus only  *Cetraria islandica* (100%) | 6.7 | † | † | † |
| Control sample – rainwater | 5.2–6.7 | † | † | † |

Table S6. Spectrophotometric analysis of rainwater extracts from the tested lichen thalli using the Folin and Ciocalteu method for the total phenol concentration; ‘hot extraction’ by Soxhlet apparatus for one extraction cycle.

Abbreviations: † – negative result; * rainwater of natural pH – no modification; ** acidic rainwater of pH 3 – modified by HCl; *** alkalised rainwater of pH 9 – modified by Na_2_CO_3_; ; ^‡^ arithmetic mean of three biological replicates with three biological measurements each, ±standard deviation.

| **Lichen species** | **Preliminary results** | | **Verification** | | |
| --- | --- | --- | --- | --- | --- |
|  | **Rainwater pH** | **Phenol concn.**  **(µg ml^-1^)** | **Rainwater pH** | | **Phenol concn.**  **(µg ml^-1^)** |
| **epigeic lichens** | | | | | |
| *Cetraria aculeata* | 4.2^*^ | † | |  |  |
|  | 3^**^ | † | |  |  |
|  | 9^***^ | † | |  |  |
| *Cetraria islandica* | 4.6 | 7.98 | | 7.4^*^ | 21.50 ±5.73^‡^ |
|  | 3 | 13.41 | | 3^**^ | 14.78 ±2.99 |
|  | 9 | 19.73 | | 9^***^ | 4.26 ±2.75 |
| *Cladonia arbuscula* | 4.2 | † | |  |  |
|  | 3 | 4.42 | |  |  |
|  | 9 | † | |  |  |
| *Cladonia digitata* | 5.7 | 24.33 | | 4.5 | 34.29 ±4.17 |
|  | 3 | 4.87 | | 3 | 14.87 ±2.43 |
|  | 9 | 3.52 | | 9 | 28.84 ±2.14 |
| *Cladonia furcata* | 5.9 | 39.63 | | 7.4 | 12.71 ±9.61 |
|  | 3 | 21.01 | | 3 | 15.47 ±2.60 |
|  | 9 | 18.77 | | 9 | 18.33 ±0.95 |
| *Cladonia gracilis* | 5.7 | 12.14 | | 4.4 | 3.71 ±1.53 |
|  | 3 | 4.78 | | 3 | 0.46 ±0.80 |
|  | 9 | 1.31 | | 9 | 6.65 ±1.67 |
| *Cladonia phyllophora* | 6.5 | 4.97 | | 6.9 | † |
|  | 3 | 8.80 | | 3 | 1.66 ±2.09 |
|  | 9 | 12.99 | | 9 | 0.91 ±1.50 |
| *Cladonia rangiferina* | 4.6 | † | |  |  |
|  | 3 | † | |  |  |
|  | 9 | † | |  |  |
| *Cladonia subulata* |  |  | | 6.7 | 10.86 ±1.11 |
|  |  |  | | 3 | 14.09 ±1.09 |
|  |  |  | | 9 | 9.65 ±2.18 |
| *Cladonia uncialis* | 4.8 | † | |  |  |
|  | 3 | † | |  |  |
|  | 9 | † | |  |  |
| *Cladonia arbuscula* + *C. rangiferina*  + *Cetraria islandica* | 6.3 | 7.10 | | 5.0 | 47.89 ±3.03 |
|  | 3 | 7.53 | | 3 | 29.44 ±1.85 |
|  | 9 | 11.71 | | 9 | 48.19 ±2.40 |
| **epiphytic lichens** | | | | | |
| *Evernia prunastri* | 4.9 | 35.58 | | 7.0 | 51.42 ±6.50 |
|  | 3 | 19.06 | | 3 | 50.93 ±3.04 |
|  | 9 | 32.02 | | 9 | 70.65 ±4.09 |
| *Hypogymnia physodes* | 6.8 | 29.67 | | 7.7 | 72.42 ±3.88 |
|  | 3 | 32.74 | | 3 | 67.76 ±2.44 |
|  | 9 | 13.22 | | 9 | 49.09 ±2.21 |
| *Parmelia serrana* | 5.1 | 32.67 | | 6.9 | 44.87 ±6.59 |
|  | 3 | 21.96 | | 3 | 41.42 ±5.46 |
|  | 9 | 21.07 | | 9 | 31.19 ±3.16 |
| *Platismatia glauca* | 5.8 | 4.83 | | 7.1 | 9.38 ±0.47 |
|  | 3 | 0.55 | | 3 | 9.16 ±1.64 |
|  | 9 | † | | 9 | † |
| *Pseudevernia furfuracea* | 5.6 | 47.87 | | 7.1 | 55.4 ±1.85 |
|  | 3 | 39.57 | | 3 | 53.19 ±3.12 |
|  | 9 | 77.78 | | 9 | 46.33 ±10.51 |
| *Nephromopsis chlorophylla* | 6.1 | 4.49 | | 6.5 | 18.66 ±0.46 |
|  | 3 | 3.94 | | 3 | 13.01 ±1.21 |
|  | 9 | 0.12 | | 9 | 7.95 ±1.53 |
| *Usnea dasopoga* | 5.4 | † | |  |  |
|  | 3 | 6.86 | |  |  |
|  | 9 | 2.36 | |  |  |
| Control sample – rainwater | 5.5 | † | | 5.5 | † |
|  | 3 | † | | 3 | † |
|  | 9 | † | | 9 | † |

Table S7. Spectrophotometric analysis of rainwater extracts from the tested lichen thalli using the Folin and Ciocalteu method for the total phenol concentration; ‘hot extraction’ by ‘tea method’ using the water heating with immersed thalli.

Abbreviations: † – negative result; * rainwater of natural pH – no modification; ** acidic rainwater of pH 3 – modified by HCl; *** alkalised rainwater of pH 9 – modified by Na_2_CO_3_; ; ^‡^ arithmetic mean of three biological replicates with three biological measurements each, ±standard deviation.

| **Lichen species** | **Rainwater pH** | **Phenol concn.**  **(µg ml^-1^)** |
| --- | --- | --- |
|  | **epigeic species** |  |
| *Cetraria aculeata* | 5.9^*^ | † |
|  | 3^**^ | † |
|  | 9^***^ | 6.18 ±2.45^‡^ |
| *Cetraria islandica* | 5.2 | 75.64 ±5.84 |
|  | 3 | 58.36 ±0.37 |
|  | 9 | 136.67 ±4.2 |
| *Cladonia arbuscula* | 5.5 | 1.53 ±1.91 |
|  | 3 | † |
|  | 9 | 33.87 ±1.66 |
| *Cladonia digitata* | 5.6 | 62.44 ±4.39 |
|  | 3 | 18.02 ±1.32 |
|  | 9 | 110.63 ±5.57 |
| *Cladonia furcata* | 5.9 | 38.00 ±0.71 |
|  | 3 | 19.37 ±2.81 |
|  | 9 | 55.52 ±5.24 |
| *Cladonia gracilis* | 6.6 | 27.93 ±2.26 |
|  | 3 | 14.20 ±1.59 |
|  | 9 | 56.48 ±5.45 |
| *Cladonia phyllophora* | 4.9 | 31.51 ±1.54 |
|  | 3 | 20.41 ±4.59 |
|  | 9 | 55.36 ±9.54 |
| *Cladonia rangiferina* | 5.6 | 23.78 ±1.39 |
|  | 3 | 2.60 ±1.4 |
|  | 9 | 48.88 ±0.56 |
| *Cladonia uncialis* | 4.8 | † |
|  | 3 | 4.79 ±5.95 |
|  | 9 | 28.45 ±2.38 |
| *Cladonia arbuscula* + *C. rangiferina*  + *Cetraria islandica* | 4.8 | 95.19 ±11.62 |
|  | 3 | 40.55 ±3.85 |
|  | 9 | 93.76 ±4.49 |
|  | **epiphytic species** |  |
| *Evernia prunastri* | 5.5 | 139.15 ±4.61 |
|  | 3 | 151.53 ±9.67 |
|  | 9 | 210.71 ±3.15 |
| *Hypogymnia physodes* | 5.3 | 116.05 ±11.31 |
|  | 3 | 156.4 ±9.15 |
|  | 9 | 186.68 ±7.28 |
| *Parmelia serrana* | 6.2 | 49.81 ±3.27 |
|  | 3 | 32.18 ±1.35 |
|  | 9 | 73.23 ±5.58 |
| *Platismatia glauca* | 6.2 | 30.56 ±3.06 |
|  | 3 | 24.89 ±0.44 |
|  | 9 | 55.73 ±2.47 |
| *Pseudevernia furfuracea* | 5.2 | 140.04 ±4.18 |
|  | 3 | 113.94 ±8.65 |
|  | 9 | 188.63 ±1.04 |
| *Nephromopsis chlorophylla* | 5.4 | 12.32 ±2.78 |
|  | 3 | 1.23 ±1.84 |
|  | 9 | 5.35 ±0.69 |
| *Usnea dasopoga* | 6.8 | 27.75 ±1.50 |
|  | 3 | 21.14 ±2.37 |
|  | 9 | 36.48 ±3.24 |
|  | 5.5 | † |
| Control sample – rainwater | 3 | † |
|  | 9 | † |
