# Supplementary material for: A spectrophotometric analysis of extracted water-soluble phenolic metabolites of lichens
Source: Planta. 2024 Jul 2;260(2):40. doi: 10.1007/s00425-024-04474-3 (PMC11219455; doi:10.1007/s00425-024-04474-3)
Supplement: Supplementary file 4 — Supplementary file4 (DOCX 136 KB) [file 425_2024_4474_MOESM4_ESM.docx]

Table S8. Significance analysis of differences for the assessment of lichen phenol extraction efficiency between the tested water extraction methods using the non-parametric U Mann-Whitney test (for group *n* <30) and the λ Kolmogorov-Smirnov and *X*^2^ (for group *n* ≥30) ones. The red colour indicates the extraction methods that differed statistically significantly in the concentration of the extracted substances, with the method for which this concentration was significantly higher indicated in bold. The significance level ɑ=0.05 was used to assess the differences significance.

Abbreviations: $\bar{x}$– arithmetic mean, SD - standard deviation, Me - median, † - mean value of the lichen phenol concentrations for all biological measurements from a given extract was zero, [*X*^2^] *X*^2^ test, [λ] Kolmogorov-Smirnov test, [S-W] Shapiro-Wilk test, [U] Mann-Whitney test.

| **Species** | **Variant 1** | | **Variant 2** | | **Group size (***n***)**  **Concn. (µg ml^-1^)** | | **Distribution normality**  **(bold – normal distribution)** | | **Statistical tests ɑ=0.05** |
| --- | --- | --- | --- | --- | --- | --- | --- | --- | --- |
|  | **extraction method** | **solvent pH** | **extraction method** | **solvent pH** | **variant**  **1** | **variant**  **2** | **variant**  **1** | **variant**  **2** |  |
| *C. islandica* | ‘cold’  10 min.  (verification) | 7.5 | **‘hot’**  **Soxhlet**  **(verification)** | 7.4 | *n* =18  $\bar{x}$ = 0.00  SD = 0.00  Me = 0.00 | *n* =18  $\bar{x}$ = 27.68 SD = 17.80  Me = 26.82 | † | **[S-W]**  ***P=*0.1213** | [U]  *P=*0.000000 |
|  |  | 3 |  | 3 |  |  |  |  |  |
|  |  | 9 |  | 9 |  |  |  |  |  |
| *C. arbuscula*  + *C. rangiferina*  + *C. islandica* |  | 7.4 |  | 5 |  |  |  |  |  |
|  |  | 3 |  | 3 |  |  |  |  |  |
|  |  | 9 |  | 9 |  |  |  |  |  |
| *C. islandica* | ‘cold’  10 min.  (verification) | 7.5 | **‘hot’**  **tea method** | 5.2 | *n* =18  $\bar{x}$ = 0.00  SD = 0.00  Me = 0.00 | *n* =18  $\bar{x}$ = 83.36 SD = 33.48  Me = 83.65 | † | **[S-W]**  ***P=*0.3835** | [U]  *P=*0.000000 |
|  |  | 3 |  | 3 |  |  |  |  |  |
|  |  | 9 |  | 9 |  |  |  |  |  |
| *C. arbuscula*  + *C. rangiferina*  + *C. islandica* |  | 7.4 |  | 4.8 |  |  |  |  |  |
|  |  | 3 |  | 3 |  |  |  |  |  |
|  |  | 9 |  | 9 |  |  |  |  |  |
| *C. islandica* | ‘cold’  10 min.  (verification) | 7.5 | ‘cold’  60 min.  (verification) | 6.4 | *n* =9  $\bar{x}$ = 0.00  SD = 0.00  Me = 0.00 | *n* =9  $\bar{x}$ = 0.21  SD = 0.63  Me = 0.00 | † | [S-W]  *P=*0.00000 | [U]  *P=*0.723932 |
|  |  | 3 |  | 3 |  |  |  |  |  |
|  |  | 9 |  | 9 |  |  |  |  |  |
| *C. islandica* | ‘cold’  10 min.  (verification) | 7.5 | **‘light-bulb method’**  **100 W**  **60 min.** | 6.7 | *n* =9  $\bar{x}$ = 0.00  SD = 0.00  Me = 0.00 | *n* =9  $\bar{x}$ = 6.48  SD = 5.19  Me = 8.48 | † | [S-W]  *P=*0.0393 | [U]  *P=*0.019284 |
|  |  | 3 |  | 3 |  |  |  |  |  |
|  |  | 9 |  | 9 |  |  |  |  |  |
| *C. islandica* | ‘cold’  10 min.  (verification) | 7.5 | **‘light-bulb method’100 W**  **120 min.** | 7 | *n* =18  $\bar{x}$ = 0.00  SD = 0.00  Me = 0.00 | *n* =18  $\bar{x}$ = 12.24 SD = 9.51  Me = 17.13 | † | **[S-W]**  ***P=*0.0836** | [U]  *P=*0.000072 |
|  |  | 3 |  | 3 |  |  |  |  |  |
|  |  | 9 |  | 9 |  |  |  |  |  |
| *C. arbuscula*  + *C. rangiferina* + *C. islandica* |  | 7.4 |  | 6.8 |  |  |  |  |  |
|  |  | 3 |  | 3 |  |  |  |  |  |
|  |  | 9 |  | 9 |  |  |  |  |  |
| *C. islandica* | ‘hot’  Soxhlet  (verification) | 7.4 | **‘hot’**  **tea method** | 5.2 | *n* =108  $\bar{x}$ = 27.69  SD = 22.42  Me = 19.97 | *n* =108  $\bar{x}$ = 74.98 SD = 57.38  Me = 58.07 | [*X*^2^] *P=*0.00000  [*λ*]  *P<*0.05 | [*X*^2^]  *P=*0.00000  [*λ*]  *P<*0.01 | [*λ*]  *P<*0.001 |
|  |  | 3 |  | 3 |  |  |  |  |  |
|  |  | 9 |  | 9 |  |  |  |  |  |
| *C. digitata* |  | 4.5 |  | 5.6 |  |  |  |  |  |
|  |  | 3 |  | 3 |  |  |  |  |  |
|  |  | 9 |  | 9 |  |  |  |  |  |
| *C. furcata* |  | 7.4 |  | 5.9 |  |  |  |  |  |
|  |  | 3 |  | 3 |  |  |  |  |  |
|  |  | 9 |  | 9 |  |  |  |  |  |
| *C. gracilis* |  | 4.4 |  | 6.6 |  |  |  |  |  |
|  |  | 3 |  | 3 |  |  |  |  |  |
|  |  | 9 |  | 9 |  |  |  |  |  |
| *C. phyllophora* |  | 6.9 |  | 4.9 |  |  |  |  |  |
|  |  | 3 |  | 3 |  |  |  |  |  |
|  |  | 9 |  | 9 |  |  |  |  |  |
| *C. arbuscula*  + *C. rangiferina*  + *C. islandica* |  | 5 |  | 4.8 |  |  |  |  |  |
|  |  | 3 |  | 3 |  |  |  |  |  |
|  |  | 9 |  | 9 |  |  |  |  |  |
| *E. prunastri* |  | 7 |  | 5.5 |  |  |  |  |  |
|  |  | 3 |  | 3 |  |  |  |  |  |
|  |  | 9 |  | 9 |  |  |  |  |  |
| *H. physodes* |  | 7.7 |  | 5.3 |  |  |  |  |  |
|  |  | 3 |  | 3 |  |  |  |  |  |
|  |  | 9 |  | 9 |  |  |  |  |  |
| *P. serrana* |  | 6.9 |  | 6.2 |  |  |  |  |  |
|  |  | 3 |  | 3 |  |  |  |  |  |
|  |  | 9 |  | 9 |  |  |  |  |  |
| *P. glauca* |  | 7.1 |  | 6.2 |  |  |  |  |  |
|  |  | 3 |  | 3 |  |  |  |  |  |
|  |  | 9 |  | 9 |  |  |  |  |  |
| *P. furfuracea* |  | 7.1 |  | 5.2 |  |  |  |  |  |
|  |  | 3 |  | 3 |  |  |  |  |  |
|  |  | 9 |  | 9 |  |  |  |  |  |
| *N. chlorophylla* |  | 6.5 |  | 5.4 |  |  |  |  |  |
|  |  | 3 |  | 3 |  |  |  |  |  |
|  |  | 9 |  | 9 |  |  |  |  |  |
| *C. islandica* | **‘hot’**  **Soxhlet**  **(verification)** | 7.4 | ‘cold’  60 min.  (verification) | 6.4 | *n* =18  $\bar{x}$ = 38.30  SD = 27.2  Me = 36.37 | *n* =18  $\bar{x}$ = 0.10  SD = 0.45  Me = 0.00 | [S-W]  *P=*0.0247 | [S-W]  *P=*0.00000 | [U]  *P=*0.000000 |
|  |  | 3 |  | 3 |  |  |  |  |  |
|  |  | 9 |  | 9 |  |  |  |  |  |
| *H. physodes* |  | 7.7 |  | 7.2 |  |  |  |  |  |
|  |  | 3 |  | 3 |  |  |  |  |  |
|  |  | 9 |  | 9 |  |  |  |  |  |
| *C. islandica* | **‘hot’**  **Soxhlet**  **(verification)** | 7.4 | ‘light-bulb method’  100 W  10 min. | 6.2 | *n* =18  $\bar{x}$ = 38.30  SD = 27.2  Me = 36.37 | *n* =18  $\bar{x}$ = 0.00  SD = 0.00  Me = 0.00 | [S-W]  *P=*0.0247 | † | [U]  *P=*0.000000 |
|  |  | 3 |  | 3 |  |  |  |  |  |
|  |  | 9 |  | 9 |  |  |  |  |  |
| *H. physodes* |  | 7.7 |  | 6.6 |  |  |  |  |  |
|  |  | 3 |  | 3 |  |  |  |  |  |
|  |  | 9 |  | 9 |  |  |  |  |  |
| *C. islandica* | **„hot”**  **Soxhlet**  **(verification)** | 7.4 | ‘light-bulb method’  100 W  60 min. | 6.7 | *n* =18  $\bar{x}$ = 38.30  SD = 27.2  Me = 36.37 | *n* =18  $\bar{x}$ = 12.81 SD = 9.92  Me = 8.68 | [S-W]  *P=*0.0247 | [S-W]  *P=*0.0364 | [U]  *P=*0.006205 |
|  |  | 3 |  | 3 |  |  |  |  |  |
|  |  | 9 |  | 9 |  |  |  |  |  |
| *H. physodes* |  | 7.7 |  | 5.7 |  |  |  |  |  |
|  |  | 3 |  | 3 |  |  |  |  |  |
|  |  | 9 |  | 9 |  |  |  |  |  |
| *C. islandica* | ‘hot’  Soxhlet  (verification) | 7.4 | ‘light-bulb method’  100 W  120 min. | 7 | *n* = 72 $\bar{x}$ = 21.99  SD = 20.98 Me = 15.43 | *n* = 72 $\bar{x}$ = 12.95 SD = 12.15 Me = 12.63 | [*X*^2^] *P=*0.00003  [*λ*]  *P<*0.05 | [*X*^2^] *P=*0.01748  **[*λ*]**  ***P<*0.15** | [*λ*]  *P<*0.10 |
|  |  | 3 |  | 3 |  |  |  |  |  |
|  |  | 9 |  | 9 |  |  |  |  |  |
| *H. physodes* |  | 7.7 |  | 6.9 |  |  |  |  |  |
|  |  | 3 |  | 3 |  |  |  |  |  |
|  |  | 9 |  | 9 |  |  |  |  |  |
| *C. furcata* |  | 7.4 |  | 7.1 |  |  |  |  |  |
|  |  | 3 |  | 3 |  |  |  |  |  |
|  |  | 9 |  | 9 |  |  |  |  |  |
| *C. arbuscula  + C. rangiferina  + C. islandica* |  | 5 |  | 6.8 |  |  |  |  |  |
|  |  | 3 |  | 3 |  |  |  |  |  |
|  |  | 9 |  | 9 |  |  |  |  |  |
| *C. digitata* |  | 4.5 |  | 6.1 |  |  |  |  |  |
|  |  | 3 |  | 3 |  |  |  |  |  |
|  |  | 9 |  | 9 |  |  |  |  |  |
| *C. phyllophora* |  | 6.9 |  | 5.9 |  |  |  |  |  |
|  |  | 3 |  | 3 |  |  |  |  |  |
|  |  | 9 |  | 9 |  |  |  |  |  |
| *C. gracilis* |  | 4.4 |  | 4.7 |  |  |  |  |  |
|  |  | 3 |  | 3 |  |  |  |  |  |
|  |  | 9 |  | 9 |  |  |  |  |  |
| *C. subulata* |  | 6.7 |  | 6.9 |  |  |  |  |  |
|  |  | 3 |  | 3 |  |  |  |  |  |
|  |  | 9 |  | 9 |  |  |  |  |  |
| *C. islandica* | **‘hot’**  **tea method** | 5.2 | ‘cold’  60 min.  (verification) | 6.4 | *n* =18  $\bar{x}$ = 121.63 SD = 46.17 Me = 130.50 | *n* =18  $\bar{x}$ = 0.10  SD = 0.45 Me = 0.00 | **[S-W]**  ***P=*0.2051** | [S-W]  *P=*0.00000 | [U]  *P=*0.000000 |
|  |  | 3 |  | 3 |  |  |  |  |  |
|  |  | 9 |  | 9 |  |  |  |  |  |
| *H. physodes* |  | 5.3 |  | 7.2 |  |  |  |  |  |
|  |  | 3 |  | 3 |  |  |  |  |  |
|  |  | 9 |  | 9 |  |  |  |  |  |
| *C. islandica* | **‘hot’**  **tea method** | 5.2 | ‘light-bulb method’  100 W  10 min | 6.2 | *n* =27  $\bar{x}$ = 85.02  SD = 65.30 Me = 77.57 | *n* =27  $\bar{x}$ = 0.00  SD = 0.00  Me = 0.00 | [S-W]  *P=*0.0420 | † | [U]  *P=*0.000000 |
|  |  | 3 |  | 3 |  |  |  |  |  |
|  |  | 9 |  | 9 |  |  |  |  |  |
| *C. arbuscula* |  | 5.5 |  | 6.7 |  |  |  |  |  |
|  |  | 3 |  | 3 |  |  |  |  |  |
|  |  | 9 |  | 9 |  |  |  |  |  |
| *H. physodes* |  | 5.3 |  | 6.6 |  |  |  |  |  |
|  |  | 3 |  | 3 |  |  |  |  |  |
|  |  | 9 |  | 9 |  |  |  |  |  |
| *C. islandica* | **‘hot’**  **tea method** | 5.2 | ‘light-bulb method’  100 W  60 min | 6.7 | *n* =27  $\bar{x}$ = 85.02  SD = 65.30 Me = 77.57 | *n* =27  $\bar{x}$ = 8.54  SD = 10.11  Me = 7.31 | [S-W]  *P=*0.0420 | [S-W]  *P=*0.0001 | [U]  *P=*0.000015 |
|  |  | 3 |  | 3 |  |  |  |  |  |
|  |  | 9 |  | 9 |  |  |  |  |  |
| *C. arbuscula* |  | 5.5 |  | 5.8 |  |  |  |  |  |
|  |  | 3 |  | 3 |  |  |  |  |  |
|  |  | 9 |  | 9 |  |  |  |  |  |
| *H. physodes* |  | 5.3 |  | 5.7 |  |  |  |  |  |
|  |  | 3 |  | 3 |  |  |  |  |  |
|  |  | 9 |  | 9 |  |  |  |  |  |
| *C. aculeata* | **‘hot’**  **tea method** | 5.9 | ‘light-bulb method’  100 W  120 min. | 4.6 | *n* =99  $\bar{x}$ = 51.36  SD = 49.70 Me = 35.44 | *n* =99  $\bar{x}$ = 9.87  SD = 11.58 Me = 5.69 | [*X*^2^] *P=*0.00225  [*λ*]  *P<*0.05 | [*X*^2^]  *P=*0.00060  [*λ*]  *P<*0.01 | [*λ*]  *P<*0.001 |
|  |  | 3 |  | 3 |  |  |  |  |  |
|  |  | 9 |  | 9 |  |  |  |  |  |
| *C. islandica* |  | 5.2 |  | 7 |  |  |  |  |  |
|  |  | 3 |  | 3 |  |  |  |  |  |
|  |  | 9 |  | 9 |  |  |  |  |  |
| *C. arbuscula* |  | 5.5 |  | 6.5 |  |  |  |  |  |
|  |  | 3 |  | 3 |  |  |  |  |  |
|  |  | 9 |  | 9 |  |  |  |  |  |
| *C. digitata* |  | 5.6 |  | 6.1 |  |  |  |  |  |
|  |  | 3 |  | 3 |  |  |  |  |  |
|  |  | 9 |  | 9 |  |  |  |  |  |
| *C. furcata* |  | 5.9 |  | 7.1 |  |  |  |  |  |
|  |  | 3 |  | 3 |  |  |  |  |  |
|  |  | 9 |  | 9 |  |  |  |  |  |
| *C. gracilis* |  | 6.6 |  | 4.7 |  |  |  |  |  |
|  |  | 3 |  | 3 |  |  |  |  |  |
|  |  | 9 |  | 9 |  |  |  |  |  |
| *C. phyllophora* |  | 4.9 |  | 5.9 |  |  |  |  |  |
|  |  | 3 |  | 3 |  |  |  |  |  |
|  |  | 9 |  | 9 |  |  |  |  |  |
| *C. rangiferina* |  | 5.6 |  | 6.7 |  |  |  |  |  |
|  |  | 3 |  | 3 |  |  |  |  |  |
|  |  | 9 |  | 9 |  |  |  |  |  |
| *C. uncialis* |  | 4.8 |  | 7.1 |  |  |  |  |  |
|  |  | 3 |  | 3 |  |  |  |  |  |
|  |  | 9 |  | 9 |  |  |  |  |  |
| *C. arbuscula*  + *C. rangiferina* + *C. islandica* |  | 4.8 |  | 6.8 |  |  |  |  |  |
|  |  | 3 |  | 3 |  |  |  |  |  |
|  |  | 9 |  | 9 |  |  |  |  |  |
| *H. physodes* |  | 5.3 |  | 6.9 |  |  |  |  |  |
|  |  | 3 |  | 3 |  |  |  |  |  |
|  |  | 9 |  | 9 |  |  |  |  |  |
| *C. islandica* | ‘cold’  60 min.  (verification) | 6.4 | ‘light-bulb method’  100 W  10 min. | 6.2 | *n* =18  $\bar{x}$ = 0.10  SD = 0.45 Me = 0.00 | *n* =18  $\bar{x}$ = 0.00  SD = 0.00  Me = 0.00 | [S-W]  *P=*0.0000 | † | [U]  *P=*0.787985 |
|  |  | 3 |  | 3 |  |  |  |  |  |
|  |  | 9 |  | 9 |  |  |  |  |  |
| *H. physodes* |  | 7.2 |  | 6.6 |  |  |  |  |  |
|  |  | 3 |  | 3 |  |  |  |  |  |
|  |  | 9 |  | 9 |  |  |  |  |  |
| *C. islandica* | ‘cold’  60 min.  (verification) | 6.4 | **‘light-bulb method’**  **100 W**  **60 min.** | 6.7 | *n* =18  $\bar{x}$ = 0.10  SD = 0.45 Me = 0.00 | *n* =18  $\bar{x}$ = 12.81 SD = 9.92  Me = 8.68 | [S-W]  *P=*0.0000 | [S-W]  *P=*0.0364 | [U]  *P=*0.000026 |
|  |  | 3 |  | 3 |  |  |  |  |  |
|  |  | 9 |  | 9 |  |  |  |  |  |
| *H. physodes* |  | 7.2 |  | 5.7 |  |  |  |  |  |
|  |  | 3 |  | 3 |  |  |  |  |  |
|  |  | 9 |  | 9 |  |  |  |  |  |
| *C. islandica* | ‘cold’  60 min.  (verification) | 6.4 | **‘light-bulb method’**  **100 W**  **120 min.** | 7 | *n* =18  $\bar{x}$ = 0.10  SD = 0.45 Me = 0.00 | *n* =18  $\bar{x}$ = 21.69 SD = 14.14 Me = 18.30 | [S-W]  *P=*0.0000 | **[S-W]**  ***P=*0.1397** | [U]  *P=*0.000026 |
|  |  | 3 |  | 3 |  |  |  |  |  |
|  |  | 9 |  | 9 |  |  |  |  |  |
| *H. physodes* |  | 7.2 |  | 6.9 |  |  |  |  |  |
|  |  | 3 |  | 3 |  |  |  |  |  |
|  |  | 9 |  | 9 |  |  |  |  |  |
| *C. islandica* | ‘light-bulb method’  100 W  10 min. | 6.2 | **‘light-bulb method’**  **100 W**  **60 min.** | 6.7 | *n* =27  $\bar{x}$ = 0.00  SD = 0.00  Me = 0.00 | *n* =27  $\bar{x}$ = 8.54  SD = 10.11  Me = 7.31 | † | [S-W]  *P=*0.0001 | [U]  *P=*0.000475 |
|  |  | 3 |  | 3 |  |  |  |  |  |
|  |  | 9 |  | 9 |  |  |  |  |  |
| *C. arbuscula* |  | 6.7 |  | 5.8 |  |  |  |  |  |
|  |  | 3 |  | 3 |  |  |  |  |  |
|  |  | 9 |  | 9 |  |  |  |  |  |
| *H. physodes* |  | 6.6 |  | 5.7 |  |  |  |  |  |
|  |  | 3 |  | 3 |  |  |  |  |  |
|  |  | 9 |  | 9 |  |  |  |  |  |
| *C. islandica* | ‘light-bulb method’  100 W  10 min. | 6.2 | **‘light-bulb method’**  **100 W**  **120 min.** | 7 | *n* =27  $\bar{x}$ = 0.00  SD = 0.00  Me = 0.00 | *n* =27  $\bar{x}$ = 14.86 SD = 15.12 Me = 16.07 | † | [S-W]  *P=*0.0015 | [U]  *P=*0.000009 |
|  |  | 3 |  | 3 |  |  |  |  |  |
|  |  | 9 |  | 9 |  |  |  |  |  |
| *C. arbuscula* |  | 6.7 |  | 6.5 |  |  |  |  |  |
|  |  | 3 |  | 3 |  |  |  |  |  |
|  |  | 9 |  | 9 |  |  |  |  |  |
| *H. physodes* |  | 6.6 |  | 6.9 |  |  |  |  |  |
|  |  | 3 |  | 3 |  |  |  |  |  |
|  |  | 9 |  | 9 |  |  |  |  |  |
| *C. islandica* | ‘light-bulb method’  100 W  60 min. | 6.7 | ‘light-bulb method’  100 W  120 min. | 7 | *n* =27  $\bar{x}$ = 8.54  SD = 10.11  Me = 7.31 | *n* =27  $\bar{x}$ = 14.86 SD = 15.12 Me = 16.07 | [S-W]  *P=*0.0001 | [S-W]  *P=*0.0015 | [U]  *P=*0.146170 |
|  |  | 3 |  | 3 |  |  |  |  |  |
|  |  | 9 |  | 9 |  |  |  |  |  |
| *C. arbuscula* |  | 5.8 |  | 6.5 |  |  |  |  |  |
|  |  | 3 |  | 3 |  |  |  |  |  |
|  |  | 9 |  | 9 |  |  |  |  |  |
| *H. physodes* |  | 5.7 |  | 6.9 |  |  |  |  |  |
|  |  | 3 |  | 3 |  |  |  |  |  |
|  |  | 9 |  | 9 |  |  |  |  |  |
| *C. islandica* | ‘cold’  120 min. | 9 | ‘cold’  60 min.  (verification) | 9 | *n* =3  $\bar{x}$ = 0.00  SD = 0.00  Me = 0.00 | *n* =3  $\bar{x}$ = 0.63  SD = 1.09 Me = 0.00 | † | [S-W]  *P=*0.0000 | [U]  *P=*0.662521 |
| *C. islandica* | ‘cold’  120 min. | 9 | ‘light-bulb method’  100 W  60 min | 9 | *n* =6  $\bar{x}$ = 0.00  SD = 0.00  Me = 0.00 | *n* =6  $\bar{x}$ = 5.44  SD = 6.25  Me = 4.24 | † | **[S-W]**  ***P=*0.0938** | [U]  *P=*0.173486 |
| *C. arbuscula* |  |  |  |  |  |  |  |  |  |
| *C. islandica* | ‘cold’  120 min. | 9 | **‘light-bulb method’**  **100 W 120 min.** | 9 | *n* =21  $\bar{x}$ = 1.09  SD = 3.47 Me = 0.00 | *n* =21  $\bar{x}$ = 14.30 SD = 11.34 Me = 12.40 | [S-W]  *P=*0.0000 | **[S-W]**  ***P=*0.0526** | [U]  *P=*0.000041 |
| *C. arbuscula* |  |  |  |  |  |  |  |  |  |
| *C. digitata* |  |  |  |  |  |  |  |  |  |
| *C. furcata* |  |  |  |  |  |  |  |  |  |
| *C. rangiferina* |  |  |  |  |  |  |  |  |  |
| *C. uncialis* |  |  |  |  |  |  |  |  |  |
| *C. arbuscula*  + *C. rangiferina* + *C. islandica* |  |  |  |  |  |  |  |  |  |

Table S9. Significance analysis of differences for the assessment of lichen phenol extraction efficiency between the tested water extraction methods for the chosen lichen ecological group (epigeic or epiphytic) by using the non-parametric λ Kolmogorov-Smirnov and *X*^2^ (for group *n* ≥30). The red colour indicates the extraction methods that differed statistically significantly in the concentration of the extracted substances. with the method for which this concentration was significantly higher indicated in bold. The significance level ɑ=0.05 was used to assess the differences significance.

Abbreviations: $\bar{x}$ – arithmetic mean, SD – standard deviation, Me – median, [*X*^2^] *X*^2^ test, [*λ*] Kolmogorow-Smirnow test, n.i. – not significant.

| **Ecological group** | **Species** | **Variant 1** | | **Variant 2** | | **Group size (***n***)**  **Concn. (µg ml^-1^)** | | **Distribution normality**  **(bold – normal distribution)** | | **Statistical test ɑ=0.05** |
| --- | --- | --- | --- | --- | --- | --- | --- | --- | --- | --- |
|  |  | **extraction method** | **solvent pH** | **extraction method** | **Solvent pH** | **variant**  **1** | **variant**  **2** | **variant**  **1** | **variant**  **2** |  |
| Epigeic lichens | *C. islandica* | ‘hot’  Soxhlet  (verification) | 7.4 | **‘hot’**  **tea method** | 5.2 | *n* = 54  $\bar{x}$ = 16.89 SD = 15.44  Me = 15.26 | *n* = 54  $\bar{x}$ = 55.94  SD = 34.48  Me = 52.43 | [***X*^2^] *P=*0.17056**  [λ]  *P=*n.i. | **[*X*^2^] *P=*0.24333**  [λ]  *P=*n.i. | [λ]  *P<*0.001 |
|  |  |  | 3 |  | 3 |  |  |  |  |  |
|  |  |  | 9 |  | 9 |  |  |  |  |  |
|  | *C. digitata* |  | 4.5 |  | 5.6 |  |  |  |  |  |
|  |  |  | 3 |  | 3 |  |  |  |  |  |
|  |  |  | 9 |  | 9 |  |  |  |  |  |
|  | *C. furcata* |  | 7.4 |  | 5.9 |  |  |  |  |  |
|  |  |  | 3 |  | 3 |  |  |  |  |  |
|  |  |  | 9 |  | 9 |  |  |  |  |  |
|  | *C. gracilis* |  | 4.4 |  | 6.6 |  |  |  |  |  |
|  |  |  | 3 |  | 3 |  |  |  |  |  |
|  |  |  | 9 |  | 9 |  |  |  |  |  |
|  | *C. phyllophora* |  | 6.9 |  | 4.9 |  |  |  |  |  |
|  |  |  | 3 |  | 3 |  |  |  |  |  |
|  |  |  | 9 |  | 9 |  |  |  |  |  |
|  | *C. arbuscula*  + *C. rangiferina*  + *C. islandica* |  | 5 |  | 4.8 |  |  |  |  |  |
|  |  |  | 3 |  | 3 |  |  |  |  |  |
|  |  |  | 9 |  | 9 |  |  |  |  |  |
| Epithetic lichens | *E. prunastri* | ‘hot’  Soxhlet  (verification) | 7 | **‘hot’**  **tea method** | 5.5 | *n* = 54  $\bar{x}$ = 38.49 SD = 23.19  Me = 44.57 | *n* = 54  $\bar{x}$ = 93.81  SD = 68.76  Me = 91.40 | [*X*^2^] *P=*0.00109  [λ]  *P=*n.i. | [*X*^2^] *P=*0.01937  [λ]  *P=*n.i. | [λ]  *P<*0.001 |
|  |  |  | 3 |  | 3 |  |  |  |  |  |
|  |  |  | 9 |  | 9 |  |  |  |  |  |
|  | *H. physodes* |  | 7.7 |  | 5.3 |  |  |  |  |  |
|  |  |  | 3 |  | 3 |  |  |  |  |  |
|  |  |  | 9 |  | 9 |  |  |  |  |  |
|  | *P. serrana* |  | 6.9 |  | 6.2 |  |  |  |  |  |
|  |  |  | 3 |  | 3 |  |  |  |  |  |
|  |  |  | 9 |  | 9 |  |  |  |  |  |
|  | *P. glauca* |  | 7.1 |  | 6.2 |  |  |  |  |  |
|  |  |  | 3 |  | 3 |  |  |  |  |  |
|  |  |  | 9 |  | 9 |  |  |  |  |  |
|  | *P. furfuracea* |  | 7.1 |  | 5.2 |  |  |  |  |  |
|  |  |  | 3 |  | 3 |  |  |  |  |  |
|  |  |  | 9 |  | 9 |  |  |  |  |  |
|  | *N. chlorophylla* |  | 6.5 |  | 5.4 |  |  |  |  |  |
|  |  |  | 3 |  | 3 |  |  |  |  |  |
|  |  |  | 9 |  | 9 |  |  |  |  |  |
| Epigeic lichens | *C. islandica* | ‘hot’  Soxhlet  (verification) | 7.4 | ‘light-bulb method’  100 W  120 min. | 7 | *n* = 63  $\bar{x}$ = 16.12 SD = 14.43  Me = 13.35 | *n* = 63  $\bar{x}$ = 10.35  SD = 9.86  Me = 9.50 | [*X*^2^] *P=*0.04827  **[λ]**  ***P<*0.15** | [*X*^2^] *P=*0.00000  **[λ]**  ***P<*0.10** | [λ]  *P>*0.10 |
|  |  |  | 3 |  | 3 |  |  |  |  |  |
|  |  |  | 9 |  | 9 |  |  |  |  |  |
|  | *C. furcata* |  | 7.4 |  | 7.1 |  |  |  |  |  |
|  |  |  | 3 |  | 3 |  |  |  |  |  |
|  |  |  | 9 |  | 9 |  |  |  |  |  |
|  | *C. arbuscula  + C. rangiferina  + C. islandica* |  | 5 |  | 6.8 |  |  |  |  |  |
|  |  |  | 3 |  | 3 |  |  |  |  |  |
|  |  |  | 9 |  | 9 |  |  |  |  |  |
|  | *C. digitata* |  | 4.5 |  | 6.1 |  |  |  |  |  |
|  |  |  | 3 |  | 3 |  |  |  |  |  |
|  |  |  | 9 |  | 9 |  |  |  |  |  |
|  | *C. phyllophora* |  | 6.9 |  | 5.9 |  |  |  |  |  |
|  |  |  | 3 |  | 3 |  |  |  |  |  |
|  |  |  | 9 |  | 9 |  |  |  |  |  |
|  | *C. gracilis* |  | 4.4 |  | 4.7 |  |  |  |  |  |
|  |  |  | 3 |  | 3 |  |  |  |  |  |
|  |  |  | 9 |  | 9 |  |  |  |  |  |
|  | *C. subulata* |  | 6.7 |  | 6.9 |  |  |  |  |  |
|  |  |  | 3 |  | 3 |  |  |  |  |  |
|  |  |  | 9 |  | 9 |  |  |  |  |  |
| Epigeic lichens | *C. aculeata* | **‘hot’**  **tea method** | 5.9 | “ligh-bulb method’  100 W  120 min. | 4.6 | *n* = 90  $\bar{x}$ = 38.57 SD = 35.67  Me = 30.73 | *n* = 90  $\bar{x}$ = 7.74  SD = 9.23  Me = 3.14 | **[***X*^2^] *P=*0.00036  **[λ]**  ***P<*0.10** | **[***X*^2^] *P=*0.00000  [λ]  *P<*0.01 | [λ]  *P<*0.001 |
|  |  |  | 3 |  | 3 |  |  |  |  |  |
|  |  |  | 9 |  | 9 |  |  |  |  |  |
|  | *C. islandica* |  | 5.2 |  | 7 |  |  |  |  |  |
|  |  |  | 3 |  | 3 |  |  |  |  |  |
|  |  |  | 9 |  | 9 |  |  |  |  |  |
|  | *C. arbuscula* |  | 5.5 |  | 6.5 |  |  |  |  |  |
|  |  |  | 3 |  | 3 |  |  |  |  |  |
|  |  |  | 9 |  | 9 |  |  |  |  |  |
|  | *C. digitata* |  | 5.6 |  | 6.1 |  |  |  |  |  |
|  |  |  | 3 |  | 3 |  |  |  |  |  |
|  |  |  | 9 |  | 9 |  |  |  |  |  |
|  | *C. furcata* |  | 5.9 |  | 7.1 |  |  |  |  |  |
|  |  |  | 3 |  | 3 |  |  |  |  |  |
|  |  |  | 9 |  | 9 |  |  |  |  |  |
|  | *C. gracilis* |  | 6.6 |  | 4.7 |  |  |  |  |  |
|  |  |  | 3 |  | 3 |  |  |  |  |  |
|  |  |  | 9 |  | 9 |  |  |  |  |  |
|  | *C. phyllophora* |  | 4.9 |  | 5.9 |  |  |  |  |  |
|  |  |  | 3 |  | 3 |  |  |  |  |  |
|  |  |  | 9 |  | 9 |  |  |  |  |  |
|  | *C. rangiferina* |  | 5.6 |  | 6.7 |  |  |  |  |  |
|  |  |  | 3 |  | 3 |  |  |  |  |  |
|  |  |  | 9 |  | 9 |  |  |  |  |  |
|  | *C. uncialis* |  | 4.8 |  | 7.1 |  |  |  |  |  |
|  |  |  | 3 |  | 3 |  |  |  |  |  |
|  |  |  | 9 |  | 9 |  |  |  |  |  |
|  | *C. arbuscula*  + *C. rangiferina*  + *C. islandica* |  | 4.8 |  | 6.8 |  |  |  |  |  |
|  |  |  | 3 |  | 3 |  |  |  |  |  |
|  |  |  | 9 |  | 9 |  |  |  |  |  |

Table S10. Significance analysis of differences for the assessment of lichen phenol extraction efficiency obtained by the Soxhlet apparatus, ‘tea method’ and acetone extraction between the lichen ecological group (epigeic *vs* epiphytic) tested. The analysis was performed by using the non-parametric U Mann-Whitney (for group *n* <30) and λ Kolmogorov-Smirnov (for group *n* ≥30) tests. The red colour indicates the extraction variants that differed statistically significantly in the concentration of the extracted substances, with the variant for which this concentration was significantly higher indicated in bold. The significance level ɑ=0.05 was used to assess the differences significance.

Abbreviations: $\bar{x}$ – arithmetic mean, SD – standard deviation, Me – median, p = n.i. – not significant, ‡ not applicable, [*X*^2^] *X*^2^ test, [λ] Kolmogorow-Smirnow test, [S-W] Shapiro-Wilk test, [U] Mann-Whitney test.

| **Extraction method** | **Ecological lichen group** | | | | **Group size (***n***)**  **Concn. (µg ml^-1^)** | | **Distribution normality**  **(bold – normal distribution)** | | **Statistical tests**  **ɑ=0.05** |
| --- | --- | --- | --- | --- | --- | --- | --- | --- | --- |
|  | **Epigeic lichens** | | **Epiphytic lichens** | |  |  |  |  |  |
|  | **Variant 1** | **Solvent pH** | **Variant 2** | **Solvent pH** | **Variant**  **1** | **variant**  **2** | **Variant**  **1** | **Variant**  **2** |  |
| ‘hot’  Soxhlet  (verification) | *C. islandica* | 7.4 | ***E. prunastri*** | 7 | *n* = 63  $\bar{x}$ = 16.12  SD = 14.43  Me = 13.35 | *n* = 54  $\bar{x}$ = 39.50  SD = 23.19 Me = 44.57 | [*X*^2^] *P=*0.04827  **[λ]**  ***P<*0.15** | [*X*^2^] *P=*0.00109  [λ]  p =n.i | [λ]  *P<*0.001 |
|  |  | 3 |  | 3 |  |  |  |  |  |
|  |  | 9 |  | 9 |  |  |  |  |  |
|  | *C. digitata* | 4.5 | ***H. physodes*** | 7.7 |  |  |  |  |  |
|  |  | 3 |  | 3 |  |  |  |  |  |
|  |  | 9 |  | 9 |  |  |  |  |  |
|  | *C. furcata* | 7.4 | ***P. serrana*** | 6.9 |  |  |  |  |  |
|  |  | 3 |  | 3 |  |  |  |  |  |
|  |  | 9 |  | 9 |  |  |  |  |  |
|  | *C. gracilis* | 4.4 | ***P. glauca*** | 7.1 |  |  |  |  |  |
|  |  | 3 |  | 3 |  |  |  |  |  |
|  |  | 9 |  | 9 |  |  |  |  |  |
|  | *C. phyllophora* | 6.9 | ***P. furfuracea*** | 7.1 |  |  |  |  |  |
|  |  | 3 |  | 3 |  |  |  |  |  |
|  |  | 9 |  | 9 |  |  |  |  |  |
|  | *C. subulata* | 6.7 | ***N. chlorophylla*** | 6.5 |  |  |  |  |  |
|  |  | 3 |  |  |  |  |  |  |  |
|  |  | 9 |  | 3 |  |  |  |  |  |
|  | *C. arbuscula*  *+ C. rangiferina*  *+ C. islandica* | 5 |  |  |  |  |  |  |  |
|  |  | 3 |  | 9 |  |  |  |  |  |
|  |  | 9 |  |  |  |  |  |  |  |
| ‘hot’  tea method | *C. aculeata* | 5.9 | ***E. prunastri*** | 5.5 | *n* = 90  $\bar{x}$ = 38.57  SD = 35.67 Me = 30.73 | *n* = 63  $\bar{x}$ = 84.47  SD = 67.67 Me = 54.83 | [*X*^2^] *P=*0.00036  **[λ]**  ***P<*0.10** | [*X*^2^] *P=*0.00000  [λ]  *P<*0.05 | [λ]  *P<*0.001 |
|  |  | 3 |  | 3 |  |  |  |  |  |
|  |  | 9 |  | 9 |  |  |  |  |  |
|  | *C. islandica* | 5.2 | ***H. physodes*** | 5.3 |  |  |  |  |  |
|  |  | 3 |  | 3 |  |  |  |  |  |
|  |  | 9 |  | 9 |  |  |  |  |  |
|  | *C. arbuscula* | 5.5 | ***P. serrana*** | 6.2 |  |  |  |  |  |
|  |  | 3 |  | 3 |  |  |  |  |  |
|  |  | 9 |  | 9 |  |  |  |  |  |
|  | *C. digitata* | 5.6 | ***P. glauca*** | 6.2 |  |  |  |  |  |
|  |  | 3 |  | 3 |  |  |  |  |  |
|  |  | 9 |  | 9 |  |  |  |  |  |
|  | *C. furcata* | 5.9 | ***P. furfuracea*** | 5.2 |  |  |  |  |  |
|  |  | 3 |  | 3 |  |  |  |  |  |
|  |  | 9 |  | 9 |  |  |  |  |  |
|  | *C. gracilis* | 6.6 | ***N. chlorophylla*** | 5.4 |  |  |  |  |  |
|  |  | 3 |  | 3 |  |  |  |  |  |
|  |  | 9 |  | 9 |  |  |  |  |  |
|  | *C. phyllophora* | 4.9 | ***U. dasopoga*** | 6.8 |  |  |  |  |  |
|  |  | 3 |  | 3 |  |  |  |  |  |
|  |  | 9 |  | 9 |  |  |  |  |  |
|  | *C. rangiferina* | 5.6 |  |  |  |  |  |  |  |
|  |  | 3 |  |  |  |  |  |  |  |
|  |  | 9 |  |  |  |  |  |  |  |
|  | *C. uncialis* | 4.8 |  |  |  |  |  |  |  |
|  |  | 3 |  |  |  |  |  |  |  |
|  |  | 9 |  |  |  |  |  |  |  |
|  | *C. arbuscula*  *+ C. rangiferina*  *+ C. islandica* | 4.8 |  |  |  |  |  |  |  |
|  |  | 3 |  |  |  |  |  |  |  |
|  |  | 9 |  |  |  |  |  |  |  |
| acetone extraction  10 min. | *C. aculeata* | ‡ | ***E. prunastri*** | ‡ | *n* = 30  $\bar{x}$ = 14.55  SD = 19.51  Me = 2.68 | *n* = 15  $\bar{x}$ = 224.24  SD = 173.87  Me = 261.94 | [*X*^2^] *P=*0.00299  [λ]  *P<*0.05 | [S-W]  *P=*0.0060 | [λ]  *P<*0.001  [U]  *P=*0.000010 |
|  | *C. islandica* |  |  |  |  |  |  |  |  |
|  | *C. arbuscula* |  | ***H. physodes*** |  |  |  |  |  |  |
|  | *C. digitata* |  |  |  |  |  |  |  |  |
|  | *C. furcata* |  | ***P. serrana*** |  |  |  |  |  |  |
|  | *C. gracilis* |  |  |  |  |  |  |  |  |
|  | *C. rangiferina* |  | ***P. glauca*** |  |  |  |  |  |  |
|  | *C. subulata* |  |  |  |  |  |  |  |  |
|  | *C. uncialis* |  | ***P. furfuracea*** |  |  |  |  |  |  |
|  | *C. arbuscula*  *+ C. rangiferina*  *+ C. islandica* |  |  |  |  |  |  |  |  |

Table S11. Significance analysis of differences for the assessment of lichen phenol extraction efficiency between the rainwater pH variants tested. The analysis was performed by using the non-parametric Kruskal-Wallis and post-hoc Dunn tests. The normality distribution was checked by Shapiro-Wilk (for group *n* <30), *X^2^* and
λ Kolmogorow-Smirnow tests (for group *n*$\geq$30). The red colour indicates the rainwater pH variants that differed statistically significantly in the concentration of the extracted substances, with the variant for which this concentration was significantly higher indicated in bold. The significance level ɑ=0.05 was used to assess the differences significance.

Abbreviations: $\bar{x}$ – arithmetic mean, SD – standard deviation, Me – median, [X^2^] *X*^2^ test, [*λ*] Kolmogorow-Smirnow test, [S-W] Shapiro-Wilk test, [Kru-Wall] – Kruskal-Wallis test, n.i – not significant, post-hoc – post-hoc Dunn test.

| **Group** | **Extraction method** | **Lichen species** | **Group size (***n***)**  **Concn. (µg ml^-1^)** | | | **Distribution normality statistics**  **(bold – normal distribution)** | | | **Statistical tests**  **ɑ=0.05** |
| --- | --- | --- | --- | --- | --- | --- | --- | --- | --- |
|  |  |  | **Variant 1** | **Variant 2** | **Variant 3** | **Variant 1** | **Variant 2** | **Variant 3** |  |
| 1 | ‘hot’  Soxhlet (verification) | *C. islandica*  *H. physodes* | **natural pH**$\bar{x}$ pH = 6.52  *n* =36  $\bar{x}$ = 31.92  SD = 36.88  Me = 19.71 | **pH 3**  *n* =36  $\bar{x}$ = 26.76  SD = 45.83  Me = 2.48 | **pH 9**  *n* =36  $\bar{x}$ = 38.59  SD = 58.77  Me = 13.76 | [*X*^2^]  *P=*0.04241  **[*λ*]**  ***P<*0.15** | [*X*^2^] *P=*0.00000  [*λ*]  *P<*0.01 | [*X*^2^] *P=*0.00132  [*λ*]  *P<*0.05 | [Kru-Wall]  *P=*0.2449 |
|  | ‘hot’  tea method |  |  |  |  |  |  |  |  |
|  | ‘cold’  60 min (verification) |  |  |  |  |  |  |  |  |
|  | ‘light-bulb method’ 100 W  10 min |  |  |  |  |  |  |  |  |
|  | ‘light-bulb method’  100 W  60 min |  |  |  |  |  |  |  |  |
|  | ‘light-bulb method’  100 W  120 min |  |  |  |  |  |  |  |  |
| 2 | ‘hot’  Soxhlet (verification) | *C. islandica*  *C. digitata*  *C. furcata*  *C. gracilis*  *C. phyllophora*  *C. arbuscula*  *+ C. rangiferina*  *+ C. islandica*  *E. prunastri*  *H. physodes*  *P. serrana*  *P. glauca*  *P. furfuracea*  *N. chlorophylla* | **natural pH**  $\bar{x}$ pH = 6.03  *n* =72  $\bar{x}$ = 49.62  SD = 39.46  Me = 39.43 | **pH 3**  *n* =72  $\bar{x}$ = 40.13  SD = 42.94  Me = 24.63 | **pH 9**  *n* =72  $\bar{x}$ = 64.21  SD = 60.96  Me = 50.96 | [*X*^2^]  *P=*0.01739  **[*λ*]**  ***P<*0.15** | [*X*^2^] *P=*0.00000  [*λ*]  *P<*0.01 | [*X*^2^] *P=*0.00002  [*λ*]  *P<*0.05 | [Kru-Wall]  *P=*0.0213  post-hoc:  natural pH  *vs* pH 3  *P=*0.139972  natural pH  *vs* pH 9  *P=*1.000000  pH 3 *vs* **pH 9**  *P=*0.022840 |
|  | ‘hot’  tea method |  |  |  |  |  |  |  |  |
| 3 | ‘hot’  Soxhlet (verification) | *C. islandica*  *C. digitata*  *C. furcata*  *C. gracilis*  *C. phyllophora*  *C. subulata*  *C. arbuscula*  *+ C. rangiferina*  *+ C. islandica*  *H. physodes* | **natural pH**  $\bar{x}$ pH = 6.34  *n* =48  $\bar{x}$ = 20.36  SD = 19.85  Me = 15.23 | **pH 3**  *n* =48  $\bar{x}$ = 11.40  SD = 17.18  Me = 4.00 | **pH 9**  *n* =48  $\bar{x}$ = 20.52  SD = 14.07  Me = 17.81 | [*X*^2^]  *P=*0.01883  [*λ*]  ***P<*0.20** | [*X*^2^] *P=*0.00000  [*λ*]  *P<*0.01 | [*X*^2^] *P=*0.03685  [*λ*]  *P=*n.i | [Kru-Wall]  *P=*0.0001  post-hoc:  **natural pH** *vs* pH 3  *P=*0.005083  natural pH  *vs* pH 9  *P=*1.000000  pH 3 *vs* **pH 9**  *P=*0.000156 |
|  | ‘light-bulb method’  100 W  120 min |  |  |  |  |  |  |  |  |
| 4 | ‘hot’  tea method | *C. aculeata*  *C. islandica*  *C. arbuscula*  *C. digitata*  *C. furcata*  *C. gracilis*  *C. phyllophora*  *C. rangiferina*  *C. uncialis*  *C. arbuscula*  *+ C. rangiferina*  *+ C. islandica*  *H. physodes* | **natural pH**  $\bar{x}$ pH = 5.89  *n* =66  $\bar{x}$ = 27.44  SD = 32.54  Me = 16.59 | **pH 3**  *n* =66  $\bar{x}$ = 16.46  SD = 34.24  Me = 1.56 | **pH 9**  *n* =66  $\bar{x}$ = 44.49  SD = 47.52  Me = 29.77 | [*X*^2^]  *P=*0.00000  [λ]  *P<*0.05 | [*X*^2^] *P=*0.00000  [λ]  *P<*0.01 | [*X*^2^] *P=*0.00009  [λ]  *P<*0.01 | [Kru-Wall]  *P=*0.0000  post-hoc:  **natural pH** *vs* pH 3  *P=*0.008326  natural pH *vs* pH 9  *P=*0.058927  pH 3 *vs* **pH 9**  *P=*0.000000 |
|  | ‘light-bulb method’  100 W  120 min |  |  |  |  |  |  |  |  |
| 5 | ‘light-bulb method’  100 W  10 min  ‘light-bulb method’  100 W  60 min  ‘light-bulb method’  100 W  120 min | *C. arbuscula*  *C. islandica*  *H. physodes* | **natural pH**  $\bar{x}$ pH = 6.38  *n* =27  $\bar{x}$ = 10.91  SD = 15.03  Me = 0.00 | **pH 3**  *n* =27  $\bar{x}$ = 2.70  SD = 5.61  Me = 0.00 | **pH 9**  *n* =27  $\bar{x}$ = 9.79  SD = 12.15  Me = 1.65 | [S-W]  *P=*0.00002 | [S-W]  *P=*0.00000 | [S-W]  *P=*0.00008 | [Kru-Wall]  *P=*0.0379  post-hoc:  natural pH *vs* pH 3  *P=*0.151727  natural pH *vs* pH 9  *P=*1.000000  pH 3 *vs* pH 9  *P=*0.130558 |

Table S12. Lichen secondary metabolites ordered according to their biochemical groups (after Elix 2014; unless otherwise indicated) in the species thalli subjected to water extraction.

| **Metabolite biochemical class** | **Secondary metabolites** | **Lichen species** | **Ecological group** |
| --- | --- | --- | --- |
| usnic acid derivatives | usnic acid | *Cladonia arbuscula* | epigeic |
|  |  | *Cladonia mitis* |  |
|  |  | *Cladonia uncialis* |  |
|  |  | *Evernia prunastri* | epiphyte |
|  |  | *Ramalina farinacea* |  |
|  |  | *Usnea dasopoga* |  |
| orcinol depside | evernic acid | *Evernia prunastri* | epiphyte |
| β-orcinol depsides | thamnolic acid | *Cladonia digitata* | epigeic, epiphyte, epixylic |
|  | atranorin | *Cladonia furcata* | epigeic |
|  |  | *Cladonia rangiferina* |  |
|  |  | *Evernia prunastri* | epiphyte |
|  |  | *Hypogymnia physodes* |  |
|  |  | *Parmelia serrana* |  |
|  |  | *Platismatia glauca* |  |
|  |  | *Pseudevernia furfuracea* |  |
|  | chloroatranorin | *Pseudevernia furfuracea* | epiphyte |
|  | squamatic acid | *Cladonia uncialis* | epigeic |
| orcinol depsidones | physodic acid | *Hypogymnia physodes* | epiphyte |
|  |  | *Pseudevernia furfuracea* |  |
|  | 3-hydroksyphysodic acid | *Hypogymnia physodes* | epiphyte |
|  | 2'-O-methylphysodic acid, |  |  |
|  | isophysodic acid (Studzińska-Sroka and Zarabska-Bożejewicz 2016) |  |  |
| β-orcinol depsidones | protocertaric acid | *Cetraria islandica* | epigeic |
|  |  | *Hypogymnia physodes* | epiphyte |
|  |  | *Parmelia serrana* |  |
|  |  | *Ramalina farinacea* |  |
|  | fumarprotocetraric acid | *Cetraria islandica* | epigeic |
|  |  | *Cladonia furcata* |  |
|  |  | *Cladonia gracilis* |  |
|  |  | *Cladonia mitis* |  |
|  |  | *Cladonia phyllophora* |  |
|  |  | *Cladonia rangiferina* |  |
|  |  | *Cladonia subulata* |  |
|  | psoromic acid | *Cladonia arbuscula* | epigeic |
|  | physodalic acid | *Hypogymnia physodes* | epiphyte |
|  | salazinic acid | *Parmelia serrana* | epiphyte |
|  |  | *Ramalina farinacea* |  |
|  |  | *Usnea dasopoga* |  |
|  |  | *Ramalina farinacea* |  |
|  | hypoprotocetraric acid | *Ramalina farinacea* | epiphyte |
| higher aliphatic fatty acids | lichesterinic acid | *Cetraria aculeata* | epigeic |
|  |  | *Parmelia serrana* | epiphyte |
|  | rotolichesterinic acid | *Cetraria aculeata* | epigeic |
|  |  | *Cetraria islandica* |  |
|  |  | *Nephromopsis chlorophylla* | epiphyte |
|  |  | *Parmelia serrana* |  |
|  | rangiformic acid | *Cladonia mitis* | epigeic |
|  | caperatic acid | *Platismatia glauca* | epiphyte |

**Literature:**

J.A. Elix, (2014). A catalogue of standardized chromatographic data and biosynthetic relationships for lichen substances. Third edition. Published by the author, Canberra.

E. Studzińska-Sroka and D. Zarabska-Bożejewicz, (2016). Pustułka pęcherzykowata (*Hypogymnia physodes* (L.) Nyl.) – charakterystyka porostu i jego właściwości biologiczne, *Postępy fitoterapii*, 2016, **17(3)**, 200-207. (in Polish)
